# Engineered probiotic overcomes pathogen defences using signal interference and antibiotic production to treat infection in mice

**DOI:** 10.1038/s41564-023-01583-9

**Published:** 2024-01-16

**Authors:** Hackwon Do, Zhong-Rui Li, Praveen Kumar Tripathi, Sonali Mitra, Stephanie Guerra, Ananya Dash, Dulanthi Weerasekera, Nishanth Makthal, Syed Shams, Shifu Aggarwal, Bharat Bhushan Singh, Di Gu, Yongle Du, Randall J. Olsen, Christopher LaRock, Wenjun Zhang, Muthiah Kumaraswami

**Affiliations:** 1 Center for Molecular and Translational Human Infectious Diseases Research, Houston Methodist Research Institute, Houston, TX USA; 2 Department of Pathology and Genomic Medicine, Houston Methodist Hospital, Houston, TX USA; 3 Research unit of cryogenic novel material, Korea Polar Research Institute, Incheon, South Korea; 4 Department of Chemical and Biomolecular Engineering, University of California, Berkeley, CA USA; 5 Department of Microbiology and Immunology, Emory School of Medicine, Atlanta, GA USA; 6 Department of Chemistry, University of California, Berkeley, CA USA; 7 Department of Pathology and Laboratory Medicine, Weill Medical College of Cornell University, New York, NY USA; 8 Department of Medicine, Division of Infectious Diseases, Emory School of Medicine, Atlanta, GA USA; 9 Emory Antibiotic Resistance Center, Emory School of Medicine, Atlanta, GA USA; 10 Chan Zuckerberg Biohub, San Francisco, CA USA

**Keywords:** Infection, Pathogens, Antibiotics

## Abstract

Probiotic supplements are suggested to promote human health by preventing pathogen colonization. However, the mechanistic bases for their efficacy in vivo are largely uncharacterized. Here using metabolomics and bacterial genetics, we show that the human oral probiotic *Streptococcus salivarius* K12 (SAL) produces salivabactin, an antibiotic that effectively inhibits pathogenic *Streptococcus pyogenes* (GAS) in vitro and in mice. However, prophylactic dosing with SAL enhanced GAS colonization in mice and ex vivo in human saliva. We showed that, on co-colonization, GAS responds to a SAL intercellular peptide signal that controls SAL salivabactin production. GAS produces a secreted protease, SpeB, that targets SAL-derived salivaricins and enhances GAS survival. Using this knowledge, we re-engineered probiotic SAL to prevent signal eavesdropping by GAS and potentiate SAL antimicrobials. This engineered probiotic demonstrated superior efficacy in preventing GAS colonization in vivo. Our findings show that knowledge of interspecies interactions can identify antibiotic- and probiotic-based strategies to combat infection.

## Abstract (web summary)

Characterization of quorum sensing-regulated antibiotic production by *Streptococcus salivarus* and signal eavesdropping by *Streptococcus pyogenes* enables engineering of a probiotic strain to prevent pathogen signal eavesdropping and antibiotic degradation, and inhibit colonization.

## Main

Probiotics are live bacteria that confer health benefits to the host upon consumption. However, with few exceptions^1–7^, evidence for the suggested beneficial effects in vivo and mechanistic basis for protection for the vast majority of probiotics are lacking. Nevertheless, the beneficial health claims of probiotics are widely accepted and their potential to enhance disease pathogenesis via their interactions with pathogenic bacteria is overlooked. Understanding the molecular details of probiotic–pathogenic bacterial interactions in vivo and the impact of those interactions on pathogenesis is critical for patient care and essential to improve probiotic efficacy.

*Streptococcus salivarius* K12 (SAL), an over-the-counter oral probiotic, has bactericidal activity in vitro against several pathogens including *Streptococcus pyogenes*, otherwise known as group A streptococcus (GAS)^8–12^. GAS is an exclusive human pathogen that causes mild, superficial diseases such as pharyngitis and impetigo as well as life-threatening invasive diseases such as necrotizing fasciitis and streptococcal toxic shock syndrome^13,14^. GAS infections are among the top ten infectious causes of human mortality worldwide^13^. Despite the suggested health benefits of SAL, multiple clinical trials demonstrated inconclusive efficacy of SAL for prevention and treatment of GAS pharyngitis^15,16^. We therefore sought to elucidate the molecular details of interplay between probiotic (SAL) and pathogenic (GAS) bacteria and utilize the knowledge towards developing novel translational antimicrobial strategies. In this Article, we discovered that SAL and GAS are engaged in interspecies interactions via a quorum sensing peptide. SAL uses a quorum sensing peptide to control the production of a previously undescribed antibiotic. However, GAS exploits SAL peptide to activate the endogenous quorum sensing pathway and induce protease production, which promotes GAS survival by antimicrobial degradation. We used the knowledge of the interspecies interactions to re-engineer the probiotic strain that prevented cross-activation of GAS quorum sensing pathway and degradation of SAL antimicrobials and resulted in more efficacious probiotic that inhibits pathogen colonization.

## Results

### SAL produces a previously unknown antimicrobial

**Fig. 1:**
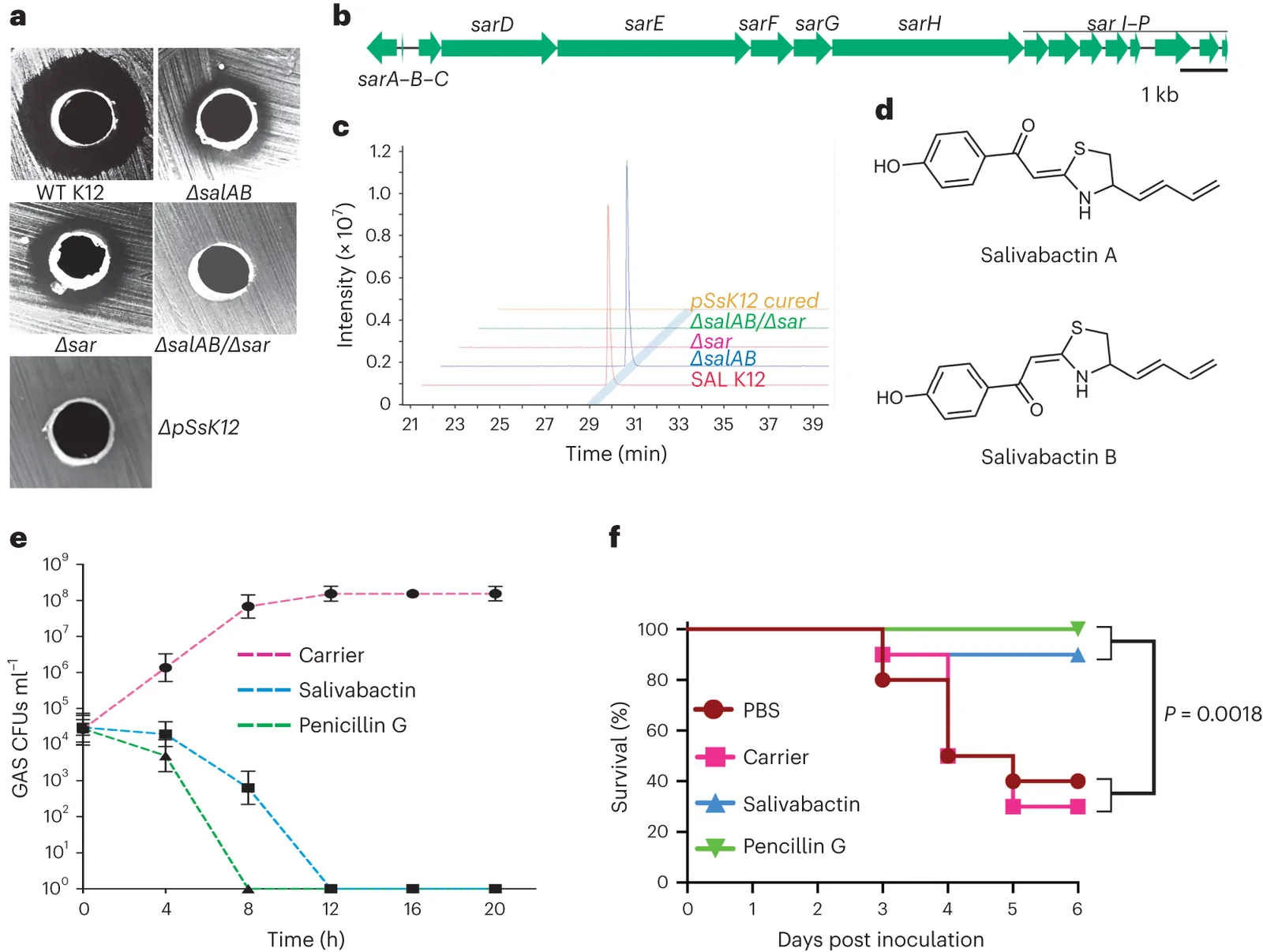
Discovery and biosynthesis of salivabactin. **a**, In vitro anti-GAS activities of various SAL strains. The indicated SAL strains were placed in a well on an agar plate containing GAS lawn. The inhibitory activity was assessed by monitoring the presence or absence of inhibitory zone around SAL growth after 16 h incubation. **b**, Organization of the 16-gene operon encoding *sar* BGC. **c**, Comparison of LC–MS extracted ion chromatogram traces of the metabolic extracts from SAL and its mutant derivatives, showing the production of salivabactin associated with the *sar* BGC. EIC+ = 274.09 ± 0.01 (EIC, extracted-ion chromatogram) corresponds to salivabactin. **d**, Chemical structure of the two salivabactin isomers, salivabactin A and salivabactin B. **e**, Time-dependent killing assay. Incubation of exponential phase GAS growth with 10× MIC of salivabactin or penicillin G caused complete killing. Data graphed represent mean ± s.d. from three biological replicates. **f**, Mice (*n* = 15 per group) were given 10^7^ CFUs of GAS intramuscularly, and antibiotics were given as a single i.m. dose at 1 h post infection. Kaplan–Meier survival curves with *P* values derived by the log-rank test are shown. Source data

SAL was thought to exert antimicrobial activity in vitro via the production of two lantibiotic peptides, salivaricin A2 (SalA) and salivaricin B (SalB)^8^. The biosynthetic gene clusters (BGCs) for *salA* and *salB* reside in a megaplasmid (*pSsK12*) and deletion of megaplasmid (∆*pSsK12*) abolished SAL anti-GAS activity (Fig. 1a and Supplementary Fig. 1). Surprisingly, the ∆*salAB* mutant, in which the production of both lantibiotics was abolished, had attenuated but distinct anti-GAS activity (Fig. 1a and Supplementary Fig. 1), suggesting that SAL produced salivaricin-independent antimicrobials and the BGCs for the previously unknown antimicrobials are also encoded on megaplasmid *pSsK12*. In silico analyses identified a unique polyketide/non-ribosomal peptide (PK/NRP) hybrid BGC (named *sar*) that was not found in any other sequenced microbial strain from public databases (Fig. 1b and Supplementary Fig. 2)^17^. Due to the well-documented role of many PK/NRPs as antibiotics^18^, we hypothesized that the *sar* BGC could be responsible for synthesizing an antimicrobial metabolite with a previously undescribed chemical scaffold. Consistent with this hypothesis, genetic inactivation of *sar* in the ∆*salAB* mutant (∆*salAB*/*∆sar*) resulted in complete loss of anti-GAS activity of SAL in vitro (Fig. 1a and Supplementary Fig. 1).

Untargeted metabolomic comparisons of the wild-type (WT) SAL and its mutant derivatives revealed a major metabolite species that was present only in the culture extracts from *sar*-containing strains (Fig. 1c). The high-resolution mass spectrometry (HRMS) analysis of the metabolite, named salivabactin, revealed a molecular formula of C_15_H_15_NO_2_S (calculated for C_15_H_16_NO_2_S^+^: 274.0896; observed: 274.0903). The ultraviolet spectrum of salivabactin demonstrated a highly conjugated chromophore, with bands at *λ*_max_ 221, 267 and 345 nm (Supplementary Fig. 3). To elucidate the structure of salivabactin, we purified 2.5 mg of salivabactin as a white amorphous powder from a total of ∼30 l of SAL growth. We deduced the molecular connectivity of salivabactin using a series of 1D (^1^H and ^13^C) and 2D (correlated spectroscopy (COSY), heteronuclear single quantum coherence (HSQC) and heteronuclear multiple-bond correlation (HMBC)) nuclear magnetic resonance (NMR) spectra of purified salivabactin (Supplementary Figs. 4–8). Interestingly, salivabactin was revealed to be an approximately equivalent mixture of two geometric isomers that were inseparable by liquid chromatography (LC) and named as salivabactin A and salivabactin B, respectively (Fig. 1d and Supplementary Figs. 4–8). The high-resolution tandem mass spectrometry (MS) analysis further confirmed the structural assignment of salivabactin (Supplementary Fig. 9). The assessment of predicted activities of the *sar*-encoded enzymes indicated the unique modality of and a direct role for *sar BGC* in salivabactin biosynthesis (Supplementary Fig. 10). The proposed biosynthetic pathway of salivabactin was supported by stable isotope feeding results using [1-^13^C_1_]acetate or [1,2-^13^C_2_]acetate, which showed the expected polyketide labelling patterns (Supplementary Fig. 11). Consequently, salivabactin represents a unique family of natural products with a previously unknown chemical scaffold that was not reported before.

### Antimicrobial activity of salivabactin

The purified salivabactin had potent anti-GAS activity with the minimum inhibitory concentration (MIC) of ~2 µg ml^−1^ (Supplementary Fig. 12a), comparable to that of several natural product antibiotics^19–22^. Salivabactin caused complete killing of GAS at 12 h post-inoculation at 10× MIC concentration, which is comparable to clinically effective anti-GAS antibiotic, penicillin G and altered GAS morphology compared with untreated cells (Fig. 1e and Supplementary Fig. 12b). Beyond GAS, salivabactin was also potent against several clinically relevant Gram-positive pathogens including group B streptococcus, *Streptococcus pneumoniae*, *Streptococcus mutans* and *Staphylococcus aureus*, while largely ineffective towards a few tested Gram-negative bacteria (Supplementary Fig. 12a). We further assessed the in vivo efficacy of salivabactin using an intramuscular (i.m.) mouse model of GAS infection that mimics human necrotizing myositis. Mice were infected intramuscularly with a lethal dose of GAS. Subsequently, a single dose of either salivabactin (i.m., 6 mg kg^−1^ of body weight) or penicillin G (i.m., 16 mg kg^−1^ of body weight) was administered at 1 h post-infection and near mortality was assessed. The protection conferred by salivabactin was comparable to penicillin G, indicating the in vivo potency of salivabactin to treat GAS infection (Fig. 1f).

### Probiotic SAL promotes GAS colonization

**Fig. 2:**
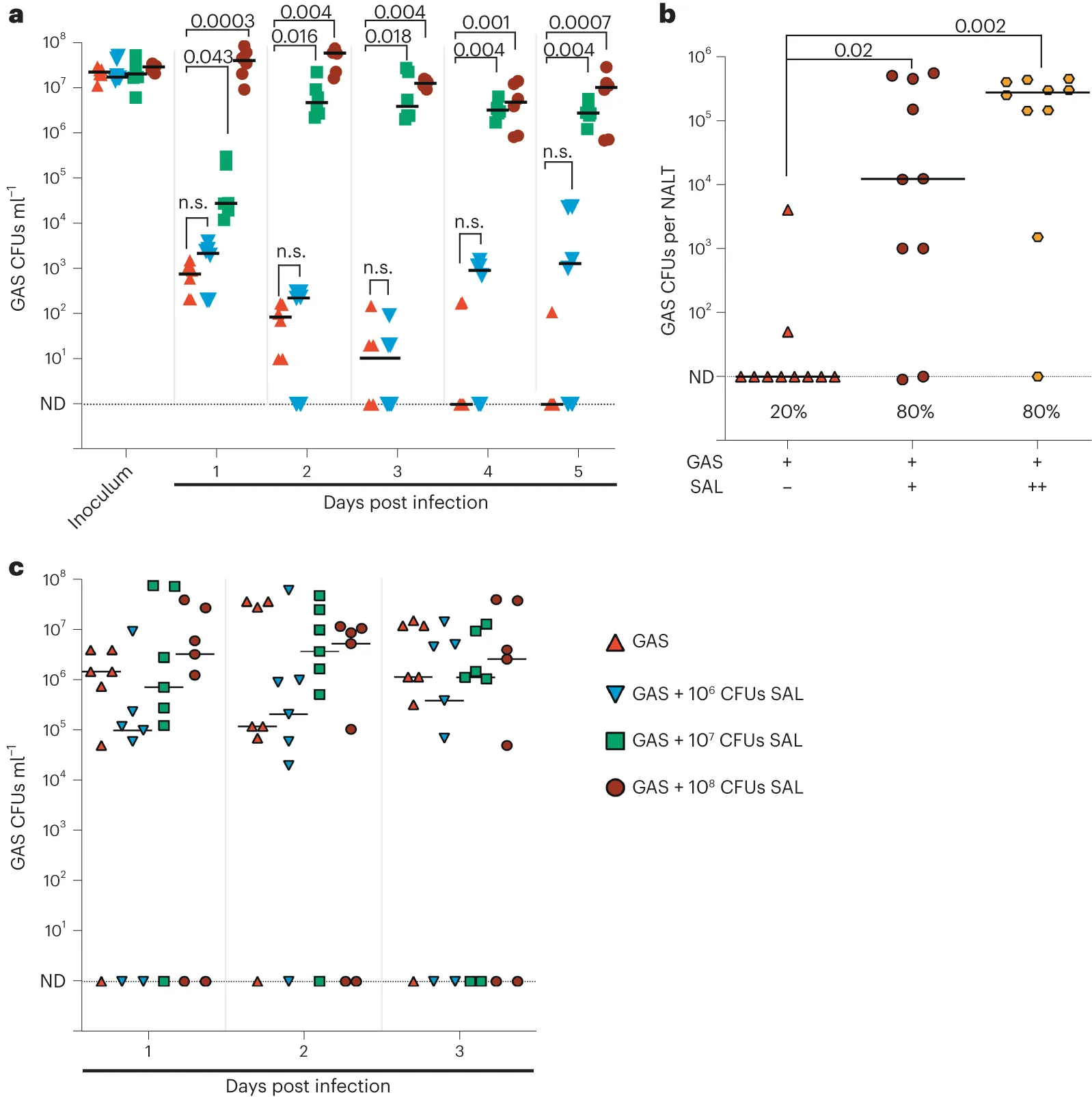
Probiotic promotes pathogen survival in human saliva ex vivo and in mouse models of infection. **a**, GAS was grown in the absence (GAS) or presence of increasing doses of SAL in sterile human saliva ex vivo. Samples were collected at the indicated timepoints and GAS levels were assessed by enumerating CFUs per millilitre of saliva. **b**, C57Bl/6 mice were inoculated intranasally with 10^8^ CFUs of GAS and/or SAL. Samples were collected at 24 h post-infection, and CFUs were enumerated. Data are pooled from two independent experiments and are presented as the median (*n* = 10). *P* values in **a** and **b** were calculated by multiple comparison Kruskal–Wallis test. **c**, CD1 mice (*n* = 5 per group) were injected with indicated doses of SAL intravaginally, and 24 h later, mice were inoculated intravaginally with 10^3^ CFUs of GAS. Samples were collected at the indicated timepoints, and GAS levels were assessed by enumerating CFUs per millilitre of swab eluate. GAS alone (GAS)-infected group was used as a control. A multiple comparison Kruskal–Wallis test was performed to determine statistically significant differences compared with GAS-infected reference group. No statistically significant differences in GAS CFUs between individual groups and reference group were observed. ND, not detected. Source data

**Fig. 3:**
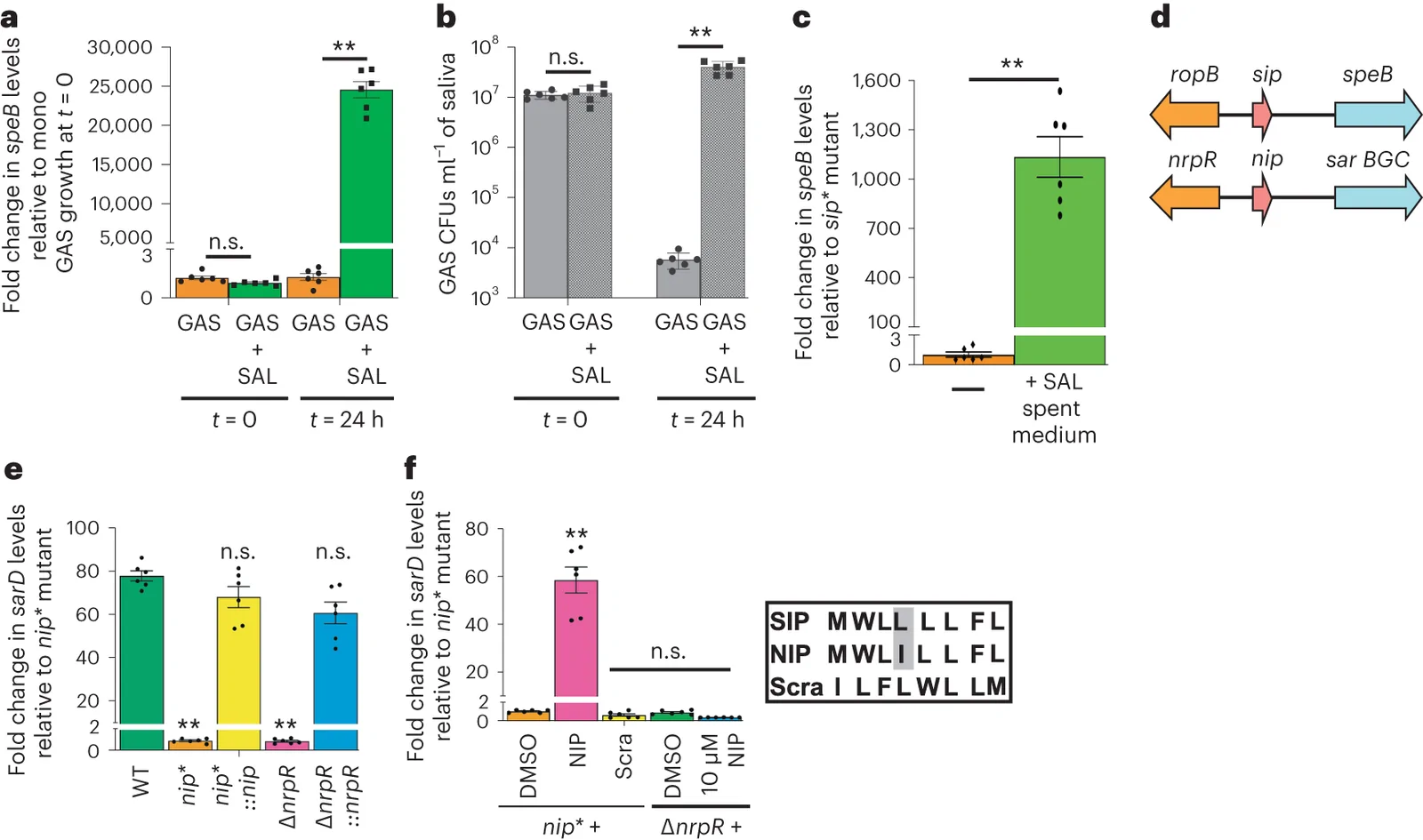
Mechanism of pathogen evasion of probiotic cytotoxicity. **a**,**b**, GAS upregulate *speB* expression (**a**) during growth with SAL that promotes GAS survival (**b**) in human saliva. Samples were collected at the indicated timepoints. *speB* expression (**a**) and GAS burden (**b**) were assessed by qRT–PCR and CFU analyses, respectively. **c**, GAS sip* mutant with a substitution of stop codon at *sip* start codon does not produce SIP and requires exogenous induction to activate *speB* expression. Incubation of sip* mutant with the cell-free culture supernatant obtained from SAL growth activates *speB* expression in GAS sip* mutant. **d**, Schematics showing the similarities between *speB*-activating *ropB-sip* quorum sensing system in GAS and the *nrpR-nip* signalling system in SAL identified in this study. **e**, The NIP and its cognate receptor NrpR controls the expression of *sar BGC*. Indicated strains were grown to late-exponential phase of growth and *sarD* transcript levels were measured by qRT–PCR. **f**, Synthetic peptide containing the amino acid sequence of NIP in native order encodes an intercellular peptide signal and activates *sarD* expression in SAL nip* mutant. In **a**–**c**, **e** and **f**, bars represent mean ± s.e.m. and statistical significance relative to reference was assessed by Mann–Whitney test. **P < 0.01; n.s., not significant. In **e**, *sarD* transcript levels in WT SAL growth were used as a reference, whereas in **f**, unsupplemented nip* mutant growth was used as a reference. Source data

Since SAL produced multiple metabolites with potent antimicrobial activity in vitro (Fig. 1a), we probed the probiotic efficacy of SAL in three host niches relevant to GAS pathogenesis, namely, human saliva where GAS and SAL could co-exist at substantial levels (~10^7^ colony-forming units (CFUs) ml^−1^) during probiotic therapy^11,23–25^, a mouse model of nasopharyngeal GAS colonization that mimics human pharyngeal GAS colonization^26–28^, and a murine model of vaginal GAS colonization, a viable alternative model to study long-term mucosal colonization of human-adapted oral streptococci that are transient colonizers of mouse nasopharynx^29^. Co-cultivation studies in human saliva ex vivo and mouse nasopharynx in vivo showed that, instead of suppressing GAS growth, SAL prevented GAS clearance and promoted GAS survival in concert with increasing SAL dosage (Fig. 2a,b and Supplementary Figs. 13 and 14). Similarly, SAL failed to inhibit GAS colonization in mouse vaginal lumen, even when SAL was given prophylactically as a single high dose (5 log-fold higher inoculum than GAS) intravaginally 24 h before GAS infection (Fig. 2c and Supplementary Fig. 14). We confirmed that *salA*, *salB* and *sarD* genes are expressed during co-infection in human saliva, mouse nasopharynx and murine vaginal lumen (Supplementary Fig. 14), suggesting that the failure of SAL in controlling GAS colonization in different host niches may not be due to the lack of anti-GAS salivabactin and lantibiotics production. Given that pathogens employ extracellular proteases to neutralize the cytotoxicity of proteinaceous antimicrobials^30,31^, we considered the alternate possibility that GAS may employ extracellular proteases during co-cultivation to negate antimicrobials and render SAL ineffective in inhibiting GAS. Consistent with this, drastic upregulation of a GAS-secreted cysteine protease SpeB, a major virulence factor^32^, was observed only during dual species growth in saliva, which also coincided with increased GAS survival (Fig. 3a,b and Supplementary Fig. 15). SAL induced *speB* expression and enhanced GAS survival ex vivo and in vivo in multiple genetically distinct GAS *emm* serotype strains, indicating that SAL–GAS interactions are conserved among GAS *emm* serotype strains (Supplementary Figs. 16 and 17).

### SAL quorum sensing peptide activates GAS protease production

The expression of *speB* is activated by a GAS quorum sensing pathway composed of a transcription activator RopB and its cognate secreted peptide signal, SpeB-inducing peptide (SIP)^33^. Intriguingly, the cell-free SAL culture supernatant was sufficient to induce *speB* expression (Fig. 3c), indicating the presence of a SIP-like secreted SAL activation factor. In accordance with this, we found that the *sarA* and *sarB* genes located immediately upstream of the *sar* BGC encode a RopB-like transcriptional activator and a SIP-like eight-amino-acid leaderless peptide signal, respectively (Fig. 3d and Supplementary Fig. 18). We thus hypothesized that SarB, termed as NRPS-inducing peptide (NIP), and SarA, termed as NRPS regulator (NrpR), constitute a secreted peptide and cognate intracellular receptor pair that activates *sar* BGC expression. Consistently, inactivation of *nip* (nip*) or *nrpR* (∆*nrpR*) abolished *sarD* expression, and both mutant strains could be genetically complemented (Fig. 3e and Supplementary Fig. 18). Furthermore, chemical complementation with synthetic NIP, but not in a scrambled order (Scra), restored *sarD* expression in nip* in an NrpR-dependent manner (Fig. 3f). Comparative transcriptome profiling of WT, ∆*nrpR* and nip* mutant strains revealed that the *sar* BGC is the major regulatory target controlled by the NIP signalling pathway in SAL (Supplementary Figs. 19 and 20).

**Fig. 4:**
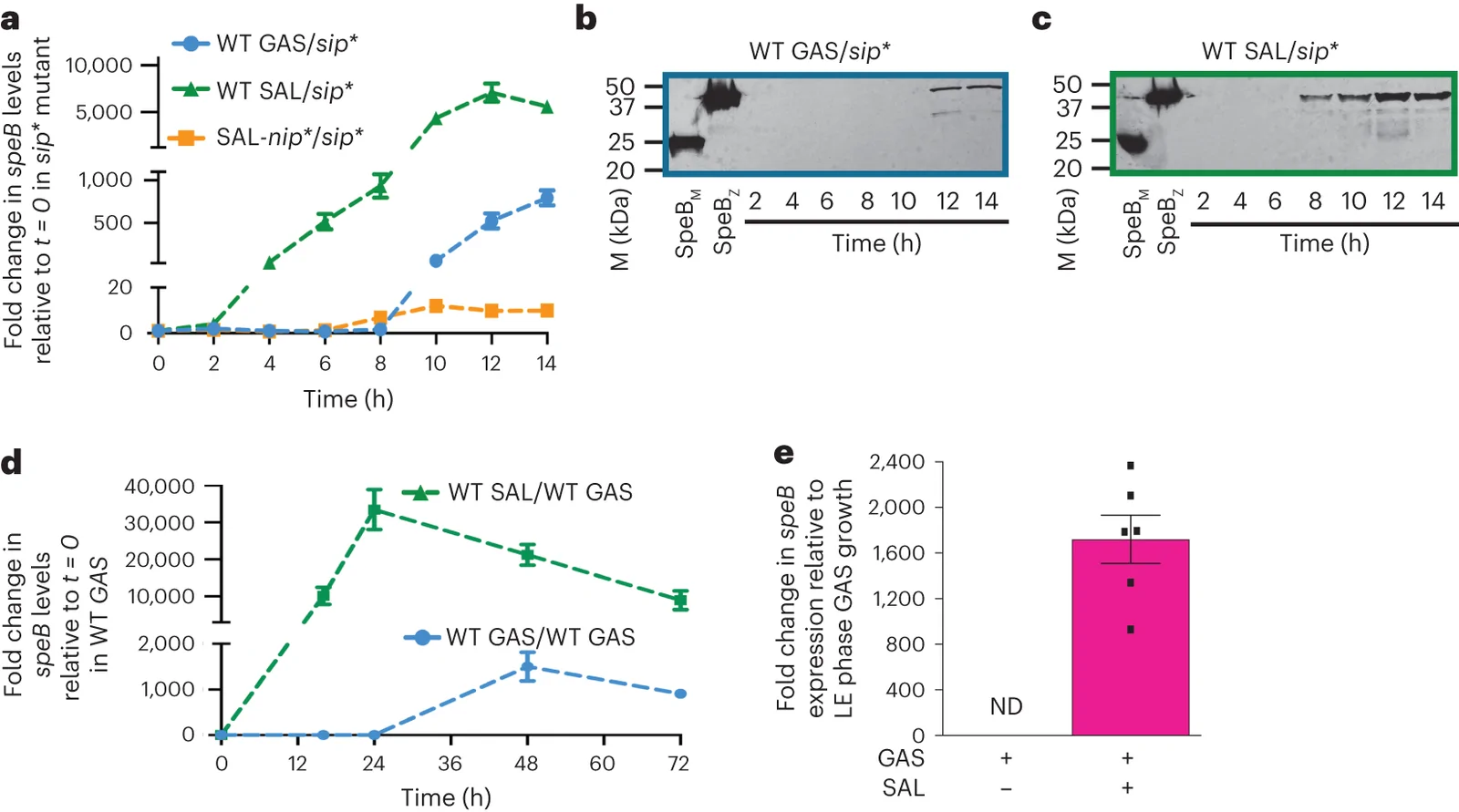
Pathogen exploits probiotic quorum sensing signal to activate secreted toxin production. **a**–**c**, SAL induces early onset and robust induction of *speB* expression (**a**) and secreted SpeB protease production (**b** and **c**) in the sip* mutant during co-cultivation in vitro in transwells. The sip* mutant in the bottom well was grown in the presence of the indicated strains in the top well. *speB* expression (**a**) and secreted SpeB protease levels (**b** and **c**) were assessed by qRT–PCR and immunoblotting, respectively. In **b** and **c**, the sip* mutant in the bottom well was grown in the presence of WT GAS and WT SAL, respectively. In **b** and **c**, SpeB_z_ indicates the enzymatically inactive higher molecular mass (~40 kDa) SpeB zymogen, whereas SpeB_M_ is the enzymatically active, lower molecular weight mature SpeB (~25 kDa). **d**, SAL induces early onset and robust induction of *speB* expression in WT GAS during co-cultivation in human saliva. Samples were collected at the indicated timepoints and analysed for *speB* expression by qRT–PCR. Data graphed are from three biological replicates that are analysed in duplicate. In **a** and **d**, data are presented as mean ± s.e.m. **e**, SAL induces *speB* expression during co-infection in mouse nasopharynx. Mice (*n* = 5 per group) were infected intranasally with GAS (10^8^ CFUs) in the presence or absence of SAL (10^8^ CFUs). The NALT was collected at 24 hpi, and fold change in *speB* transcript levels relative to late exponential (LE) phase of GAS growth in vitro is shown. ND, not detected. The low quality and reduced yield of bacterial RNA from tissues from mice infected with GAS alone prevented reliable measurement of *speB* transcript levels. Bars represent mean ± s.e.m. Source data

**Fig. 5:**
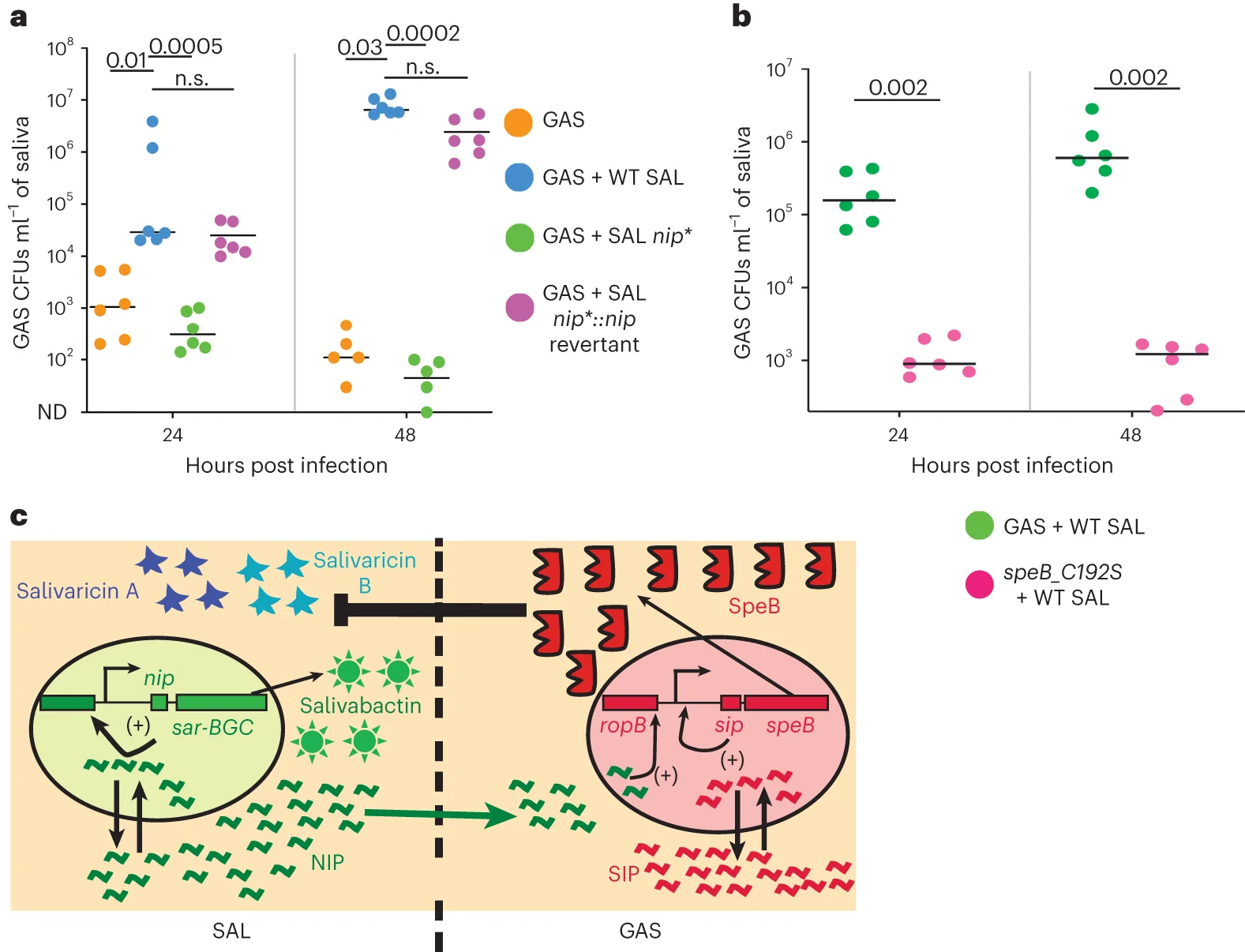
Probiotic quorum sensing signal and secreted protease are critical for pathogen survival. **a**, The NIP-producing SAL strains promote GAS survival during co-cultivation in human saliva. Equal inoculum (10^7^ CFUs ml^−1^) of GAS and indicated SAL strains was co-inoculated in saliva. **b**, Increased GAS survival in the presence of SAL requires enzymatically active SpeB. The survival of WT GAS and GAS *speB_C192S* mutant in the presence of SAL in saliva was compared. Samples were collected at the indicated timepoints, and GAS survival was assessed by CFU analyses. **c**, Model for SIP pathway activation and SpeB production by SAL. NIP produced by SAL (left) is exploited by GAS to activate endogenous SIP pathway (right). The initial activation of *sip* expression leads to robust induction of *sip* and *speB* expression by a positive feedback mechanism. SpeB proteolytically degrades SAL-derived salivaricins and promotes GAS survival during dual species growth. In **a**, GAS + WT SAL group as reference, and statistical significance was analysed by Kruskal–Wallis test. ND indicates limit of detection that was set at 10. In **b**, statistical significance was assessed by two-tailed Mann–Whitney test. n.s., not significant. Source data

Since NIP and SIP differ by one conserved amino acid change (Fig. 3f), it is likely that these leaderless peptide signals foster interspecies interactions, especially the activation of GAS *speB* expression by SAL-derived NIP. In accordance with this, genetic or biochemical complementation of GAS sip* mutant with NIP activated *speB* expression in a RopB-dependent manner in vitro and in vivo (Supplementary Figs. 21–23). Similarly, exogenous addition of SIP to SAL nip* mutant activated *sarD* expression in an NrpR-dependent manner (Supplementary Fig. 21), demonstrating the leaderless peptide-mediated crosstalk between both species. However, subsequent co-inoculation studies in vitro, in human saliva ex vivo and in mouse nasopharynx showed that the interspecies communication via leaderless peptides was unidirectional with only NIP-producing SAL activating *speB* expression in GAS, while GAS was unable to induce *sarD* expression in SAL nip* mutant (Fig. 4a–e and Supplementary Figs. 24 and 25). This phenotype was explained in part by *nip* expression during all phases of SAL growth, which is contrary to the high GAS population density-specific *sip* expression in GAS (Supplementary Fig. 26). Given that GAS exploits NIP produced by SAL, we next examined the contribution of NIP-induced *speB* expression to GAS growth augmentation by SAL in saliva. The SAL nip* mutant failed to promote GAS survival in human saliva, whereas the nip*::nip revertant and ∆*sar* strain with intact *nip* restored salivary GAS persistence similar to the WT SAL (Fig. 5a and Supplementary Fig. 27). In addition, contrary to the increased persistence of the WT GAS, no growth augmentation of GAS *speB*_*C192S* mutant encoding catalytically inactive SpeB was observed in the presence of SAL (Fig. 5b and Supplementary Fig. 27). These results implicate both NIP and SpeB as critical players in GAS defence against SAL- and host-derived antimicrobials in saliva and SAL-mediated augmentation of GAS survival during dual species growth (Fig. 5c).

To explain the contribution of SpeB, we hypothesized that the promiscuous SpeB protease aids GAS survival by degrading SAL lantibiotics and other host-derived antibacterial polypeptides. This was supported by the anti-GAS activity assays using purified SpeB and C192S mutant proteins and increased sensitivity of GAS *speB_C192S* mutant to lantibiotic extracts (Supplementary Figs. 28 and 29). As expected, SpeB did not degrade salivabactin as it lacks an amide bond based on LC–HRMS analysis (Supplementary Fig. 29c), further distinguishing this novel antibiotic from other SAL antimicrobial peptides. However, the insensitivity of salivabactin to SpeB proteolysis is probably mitigated by the relatively short-lived *sar* BGC expression and consequent transient production of salivabactin in vitro (Supplementary Figs. 14, 24 and 30), which explains the limited antagonistic effect of salivabactin towards GAS during dual species growth.

### Re-engineered SAL prevents GAS colonization

**Fig. 6:**
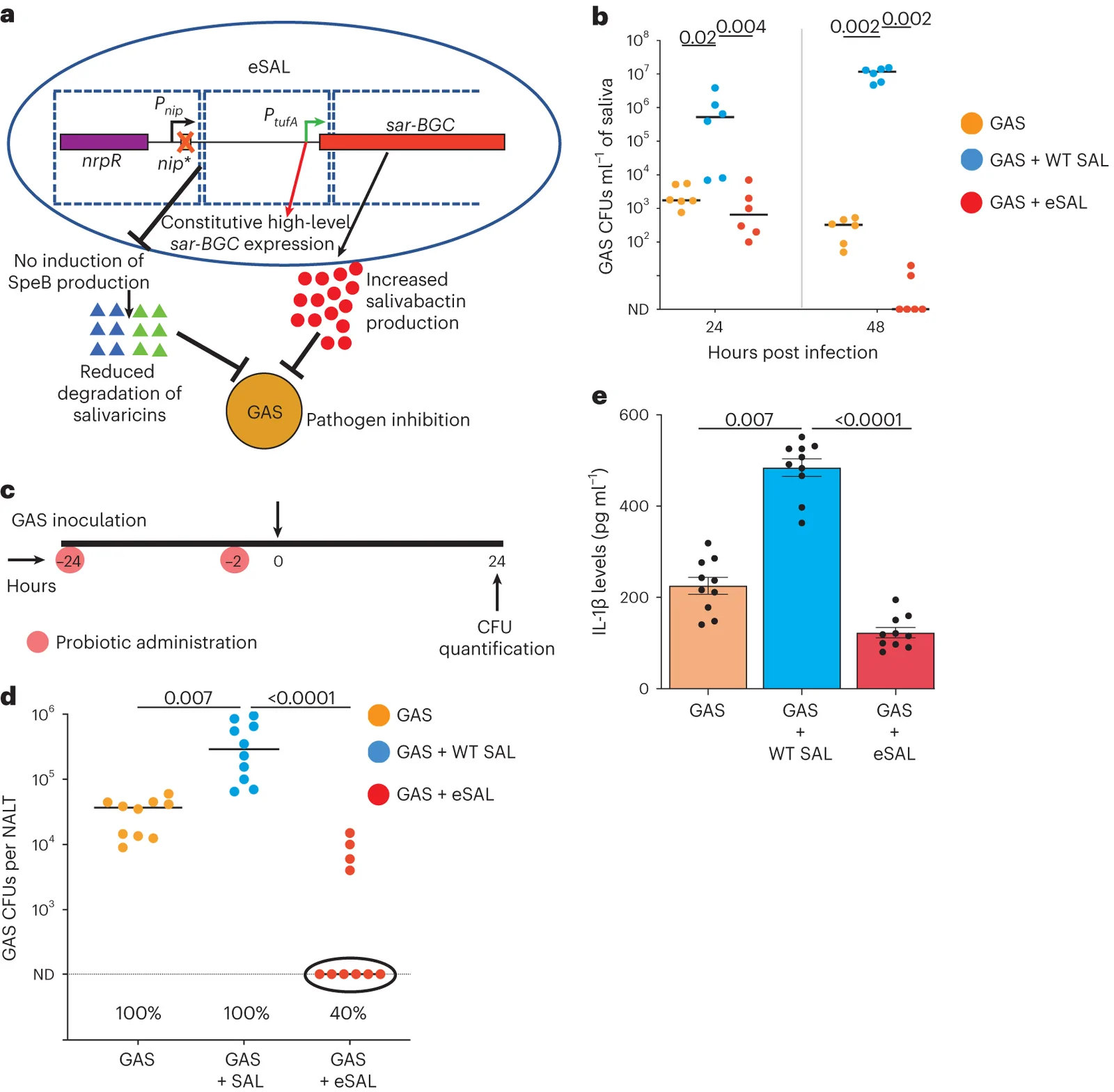
Inhibition of GAS colonization by an engineered probiotic. **a**, Schematics of genetic modifications in engineered *S. salivarius* (eSAL). The nip* mutation was introduced to abolish NIP production in *S. salivarius* and early induction of SpeB production in GAS during dual species growth. Transcription of *sar* operon was coupled with constitutively active *P*_*tufA*_ promoter that drives high-level *sar BGC* expression. Delayed SpeB protease production is likely to disarm pathogen defence and result in increased salivaricin levels due to reduced degradation by SpeB. Collectively, increased salivaricin and salivabactin levels may lead to improved pathogen inhibition and clearance of GAS in the host. **b**, GAS clearance from saliva by engineered probiotic (eSAL) as assessed by CFU analyses. **c**, Experimental design to assess the probiotic efficacy of eSAL in vivo. Each group (*n* = 10 mice per group) received 10^8^ CFUs of either WT SAL or eSAL intranasally at the indicated timepoints. One day after the first dose of probiotic administration, single dose (10^8^ CFUs) of GAS was given intranasally. GAS burden was assessed by CFU analyses. **d**, eSAL was more efficacious than WT SAL in preventing GAS colonization in mouse nasopharynx. The circles indicate the lack of detectable GAS colonies in eSAL-treated group. The numbers below indicate the percentage of animals colonized by GAS in each group. **e**, IL-1β levels in nasopharyngeal swabs as assessed by ELISA. In **b** and **d**, ND indicates limit of detection. In **b**, the detection limit was set at zero as GAS was cleared from saliva in eSAL-treated group, whereas in **d**, ND was set at 100. In **b** and **d**, data represent the geometric mean from three independent experiments that was analysed in duplicate. In **e**, data are presented as mean ± s.e.m. Statistical significance was analysed by Kruskal–Wallis test. In **b**, **d** and **e**, GAS + WT SAL group was used as a reference. Source data

Based on the characterization of the GAS defence mechanism against SAL, we hypothesized that disarming GAS defence by delayed *speB* expression via abolishing NIP production (nip*) and/or increasing salivabactin production using a constitutively active promoter (*P*_*tufA*_*-sar* BGC) may yield an engineered *S. salivarius* K12 (eSAL) with superior efficacy in controlling GAS colonization in vivo. The inactivation of *nip* alone had pronounced effect on *speB* expression and GAS growth inhibition in saliva ex vivo. However, increased salivabactin production itself did not affect enhanced GAS survival phenotype but resulted in delayed *speB* expression compared with SAL (Supplementary Fig. 31). Thus, we constructed the eSAL strain by introducing both nip* mutation as well as coupling the transcription of *sar* BGC with a NIP-independent constitutively active promoter (Fig. 6a and Supplementary Fig. 32). The genetic modifications in eSAL were stable over 100 generations in vitro and did not incur significant fitness cost on eSAL survival in multiple host niches (Supplementary Figs. 33 and 34). Consistent with the saliva studies using SAL nip* mutant (Fig. 5a), eSAL failed to induce *speB* expression in saliva and produced more salivabactin, leading to GAS clearance in saliva (Fig. 6b and Supplementary Fig. 32). The suggested dosage for SAL K12 as a prophylactic probiotic is 2.5 × 10^9^ total CFUs for every 6–12 h. Thus, to test the in vivo prophylactic efficacy of eSAL, the WT or eSAL were given at 10^8^ CFUs per dose at 24 h and 2 h before intranasal GAS infection (Fig. 6c). Contrary to WT SAL-mediated enhancement of GAS colonization, eSAL reduced GAS burden significantly and decreased GAS colonization in mouse nasopharynx (Fig. 6d). Production of the proinflammatory cytokines such as IL-1β and IL-6 levels is elevated during human GAS pharyngitis^34^ and mouse nasopharyngeal GAS colonization, correlating with disease and bacterial burden^26^. However, recent studies indicated that administration of non-K12 *S. salivarius* strains alone increases transcript levels of IL-6, but not IL-1β^26^. Functional IL-1β signalling is essential for both the induction of IL-6 and for GAS survival in the nasopharynx^26^. Thus, we measured IL-1β levels to assess the impact of eSAL on GAS pathogenesis. Consistent with the increased GAS burden in the presence of SAL, the IL-1β levels increased in the presence of SAL. However, the eSAL-treated group had significantly reduced IL-1β levels compared with GAS alone or SAL-treated groups (Fig. 6e). These findings suggest that eSAL administration reduces GAS pathogenesis in murine nasopharynx.

To test the in vivo efficacy of eSAL on prolonged mucosal GAS colonization, we administered WT or eSAL intravaginally 24 h before GAS infection, and additional doses were administered every 12 h (Supplementary Fig. 35). The *sar* BGC was expressed constitutively in eSAL during vaginal colonization compared to transient expression in WT SAL (Supplementary Fig. 35a). Unlike WT SAL, eSAL abolished GAS colonization in mouse vaginal lumen (Supplementary Fig. 35c). Salivabactin as well as eSAL strain did not cause significant alterations in salivary, murine nasopharyngeal and murine vaginal microbiome (Supplementary Figs. 36–38). These results demonstrated that inactivation of *nip* and decoupling of *sar* BGC expression from *nip* disarmed pathogen defence without causing significant microbiota dysbiosis and augmented probiotic arsenals, yielding a potent probiotic against GAS colonization both in human saliva and in vivo.

## Discussion

In this study, we discovered an antibiotic of previously undescribed chemical scaffold produced by a human probiotic that is potent against clinically significant Gram-positive pathogens. We also uncovered an innovative strategy employed by the pathogen to subvert probiotic and possibly host arsenals to promote colonization and pathogenesis. The pathogen uses a probiotic leaderless communication peptide (LCP) signal controlling antibiotic production to induce early and robust production of a key virulence factor that negates the cytotoxicity of probiotic antimicrobial peptides. Although our results demonstrate the exploitation of probiotic LCP by the pathogen, it is unlikely that GAS evolved the LCP system specifically to use SAL LCPs. We speculate that GAS may exploit similar LCPs encoded in the genomes of co-colonizing bacteria and augment its virulence potential during infection. In accordance with this, a recent study showed the prevalence of hydrophobic LCPs in a vast array of Firmicutes encompassing various human-related biomes^35^, suggesting that LCP-based interactions between GAS and members of microbiota may occur.

A recent study showed that non-K12 *S. salivarius* strains promote allergic rhinitis pathogenesis due to its increased ability to adhere to allergen-exposed host epithelial cells and trigger cytokine expression^36^. Intriguingly, the non-K12 *S. salivarius* strains also induced the expression of cytokines including IL-6 even in the absence of allergens^36^. Though *S. salivarius* is a common commensal and generally recognized as safe as a probiotic as well as anti-inflammatory in nature, the relevance of *S. salivarius*-dependent induction of cytokines to GAS pathogenesis requires further investigation. Finally, in a bench-to-bedside approach, we engineered the probiotic gene regulatory circuit to disarm the pathogen defence and augment antibiotic production, and demonstrated superior efficacy of the engineered probiotic in inhibiting pathogen colonization in vivo. Our discoveries provided unprecedented insights into the molecular interplay between a probiotic and a pathogenic bacteria in the host and enabled the development of a previously unknown small-molecule antibiotic and a more efficacious probiotic strain to control a human-associated bacterial infection.

## Methods

### Bacterial strains, plasmids and growth conditions

Bacterial strains and plasmids used in this study are listed in Supplementary Table 1. MGAS10870 is representative of serotype M3 GAS strains that cause invasive infections and has WT sequences for all known major regulatory genes^37^. *Streptococcus salivarius* K12 (SAL) is a previously described strain isolated from the oral cavity of a healthy child whose genome has been fully sequenced^38^. *Streptococcus salivarius* K12 with erythromycin resistance marker inserted at locus *RSSL_00112* was used for co-culture studies ex vivo and in vivo. *Escherichia coli* DH5α strain was used as the host for plasmid constructions, and BL21(DE3) strain was used for recombinant protein overexpression. GAS was grown routinely on Trypticase Soy agar containing 5% sheep blood (blood tryptic soy agar, BSA, Becton Dickinson) or in Todd Hewitt broth containing 0.2% (w/v) yeast extract (THY; DIFCO). When required, kanamycin or ampicillin was added to a final concentration of 50 µg ml^−1^ or 100 µg ml^−1^, respectively. Chloramphenicol was used at a final concentration of 15 µg ml^−1^. All GAS growth experiments were done in triplicate on three separate occasions for a total of nine replicates. Overnight cultures were inoculated into fresh media to achieve an initial absorption at 600 nm (*A*_600_) of 0.03. Bacterial growth was monitored by measuring the absorption at 600 nm (*A*_600_). The *E. coli* strain used for protein overexpression was grown in lysogeny broth (Fisher).

### Construction of isogenic mutant and revertant strains

Isogenic strains containing either single codon changes or inactivation of entire coding region were generated as previously described^39^. A DNA fragment with approximately 600 bp on either side of the coding region of interest was amplified using the primers listed in Supplementary Table 2 and cloned into the multi-cloning site of the temperature-sensitive plasmid pJL1055 (ref. ^40^). The resultant plasmids were introduced into SAL by competence-based DNA uptake. Briefly, overnight SAL growth was diluted in 0.3 ml of chemically defined medium^33^ and incubated at 37 °C for 75 min. Subsequently, synthetic competence-stimulating (ComS) peptide with the amino acid sequence of LPYFAGCL and plasmid was added to the cells and incubated for 3 h at 37 °C. Cells were plated on agar plates containing appropriate antibiotics. For genetic manipulation in GAS, the plasmids were electroporated into group A streptococci. The plasmid pJL1055 containing the intact genes was used to generate SAL genetic revertant strains that has the gene of interest re-introduced into its original genetic locus. Colonies with plasmid incorporated into the GAS chromosome or SAL megaplasmid were selected for subsequent plasmid curing. DNA sequencing was then performed to ensure that no spurious mutations were introduced.

### Antimicrobial activity assay

The antimicrobial activity of SAL against GAS was assessed by screening indicated SAL strains for the capacity to inhibit GAS growth. GAS grown to mid-exponential phase of growth was swabbed on THY agar plate. The agar plugs were removed, and 20 µl of each SAL strain was placed in separate wells. The plates were incubated for 16–24 h at 37 °C and zone of inhibition indicating antimicrobial activity was visualized.

### RNA-seq analysis

SAL strains were grown in THY medium to mid-exponential phase of growth (*A*_600_ ≈ 2.0) and a total of three biological replicates per strain were used. Total RNA was purified using a RNeasy (Qiagen) mini kit according to the manufacturer’s protocol. RNA was analysed for quality and concentration with an Agilent 2100 Bioanalyzer. Ribo-zero treatment kit (Epicenter) was used to remove the ribosomal RNA according to manufacturer’s protocol, and samples were further purified using the Min-Elute RNA purification kit (Qiagen). The ribosomally RNA-depleted sample was used to synthesize adaptor tagged complementary DNA libraries using the ScriptSeq V2 RNA-seq library preparation kit (Epicenter). cDNA libraries were then run on a NextSeq using the Illumina v2 reagent kit (Illumina). Approximately 10 million reads were obtained per sample, and the reads were mapped to the *S. salivarius* K12 genome^38^ using the CLC-Genomics WorkBench, version 5 (CLC Bio). For RNA sequencing (RNA-seq) analysis, the total number of reads per gene between the replicates was normalized by TPKM ((transcripts/kilobase of gene)/(million reads aligning to the genome)). Using the TPKM values, pair-wise comparisons were carried out between the two samples to identify the differentially expressed genes. Genes with twofold difference and *P* < 0.05 after applying Bonferroni’s correction were considered to be statistically significant.

### LC–HRMS-based analysis of *sar* BGC metabolites

To determine the secondary metabolite(s) whose production was associated with the *sar* gene cluster, five fermentation extracts were prepared, including WT *S. salivarius* K12, its Δ*salAB* mutant, Δ*sar* mutant, Δ*salAB*/Δ*sar* mutant and the pSsK12-cured *S. salivarius* K12. Then, the metabolite profiles of these samples were compared via LC–HRMS-based analysis, which was performed using an Agilent Technologies 6545 Accurate-Mass QTOF LC–MS instrument fitted with an Agilent Eclipse Plus C18 column (4.6 × 100 mm) by gradient elution (A, water with 0.1% formic acid; B, acetonitrile with 0.1% formic acid: 2% B over 2 min, 2% to 100% B from 2 to 53 min, 100% B from 53 to 55 min, 100% to 2% B from 55 to 55.1 min and 2% B from 55.1 to 60 min; flow rate, 0.5 ml min^−1^). The MS settings were as follows: positive ion mode, capillary voltage 3,500 V, nebulizer pressure 276 kPa (40 psi), gas temperature 320 °C, fragmentor voltage 150 V, skimmer voltage 65 V. LC–MS-measured accurate mass spectra were recorded across the range 100–1,700 *m*/*z*. All MS data were analysed using Agilent MassHunter Qualitative Analysis software. For the MS/MS analyses of targeted compounds, fragmentations were acquired with a collision energy of 15 V.

### Feeding experiments for the biosynthetic pathway study

In this assay, the seed culture of the tested strain was inoculated in fresh THY medium supplemented with 0.5 mg ml^−1^ [1-^13^C_1_]acetate or 0.5 mg ml^−1^ [1,2-^13^C_2_]acetate (Cambridge Isotope Laboratories) in a 50-ml sterile Falcon tube. After incubation, each sample was analysed as described above for LC–HRMS analysis.

### Fermentation and isolation of salivabactin

To obtain sufficient quantity of salivabactin for NMR characterization studies, the *S. salivarius* K12 mutant Δ*salAB* was carried out for the fermentation. A single colony of Δ*salAB* was picked from a freshly streaked THY (Todd Hewitt broth + 3 g l^−1^ yeast extract) agar plate and inoculated into 20 ml of THY medium at 37 °C anaerobically. After overnight culture, this culture seed was added to 2 l of fresh THY medium supplemented with 0.5 g l^−1^ cysteine. After 6 h of culture at 37 °C anaerobically, the bacterial broth was extracted with ethyl acetate twice, then the organic extracts were concentrated in vacuo. For the purification, the salivabactin crude extract was first subjected to a flash chromatography system (Sepra C18 sorbent, 250 g), and fractionated by elution with methanol:water gradient. Then, the fraction that contains the target compound was further subjected to two rounds of high-pressure LC purification with a semi-preparative C18 Phenomenex Luna column (5 μm, 250 × 10 mm inner diameter), and eluted with acetonitrile:water gradient mixtures. In total, 30 l THY broth was prepared, and 2.5 mg of salivabactin (as the mixture of salivabactin A and salivabactin B; 1:1) was isolated as a white amorphous powder.

### NMR characterization of salivabactin

The 1D and 2D NMR spectra of salivabactin, including ^1^H, ^13^C, ^1^H–^1^H COSY, ^1^H–^13^C HSQC and ^1^H–^13^C HMBC spectra, were acquired, respectively, on a Bruker Avance 900 NMR spectrometer (900 MHz for ^1^H and 225 MHz for ^13^C) equipped with a cryoprobe. For the NMR test, the sample was dissolved in dimethyl sulfoxide (DMSO)-*d*_6_ (Cambridge Isotope Laboratories). Data were collected and reported as follows: chemical shift, integration multiplicity (s, singlet; d, doublet; t, triplet; m, multiplet) and coupling constant. Chemical shifts were reported using the DMSO-*d*_6_ resonance as the internal standard for ^1^H-NMR DMSO-*d*_6_: *δ* = 2.50 p.p.m. and ^13^C-NMR DMSO-*d*_6_: *δ* = 39.5 p.p.m.

### MIC estimation

The MIC was determined by broth microdilution according to clinical and laboratory standards institute guidelines. Bacterial cultures grown to exponential phase of growth were diluted to approximately 5 × 10^5^ CFUs ml^−1^ and placed in 96-well plates. A twofold serial dilution of salivabactin was made, and 2 µl of each dilution was added to individual wells. The minimum salivabactin concentration at which no visible bacterial growth occurred after 16–24 h incubation at 37 °C and was determined as MIC. MIC assays were done in biological triplicate.

### Bactericidal assay

GAS grown to exponential phase was diluted to 10^5^ CFUs ml^−1^. Salivabactin was added at 10× MIC (20 µg ml^−1^). A 100-µl aliquot was collected at each timepoint, cells were centrifuged and pellets were resuspended in 100 µl phosphate-buffered saline (PBS). Cells were serially diluted and plated on blood agar plates. After overnight incubation at 37 °C, CFUs were enumerated and reported as CFUs ml^−1^. Penicillin G was used as the positive control in the bioassays with the same application concentration of salivabactin. Bactericidal assays were performed in biological triplicate.

### Co-cultivation studies ex vivo in human saliva

GAS and SAL colonize two distinct host compartments in human oropharynx, SAL in the tongue dorsum and GAS in posterior oropharynx^41–43^. Thus, direct community-wide interactions in vivo between the two host cell-attached streptococcal population appear unlikely. However, due to host epithelial cell shedding and other mechanisms, the sessile bacteria are continuously released into saliva. Consistent with this, the human saliva has significant load (~10^7^ CFUs ml^−1^) of both bacteria^11,23–25,44^. Thus, human saliva is a physiologically relevant host environment to investigate interspecies interactions between GAS and SAL. Saliva from adult volunteers was collected on ice under a protocol approved by the Institutional Review Board at Houston Methodist Research Institute (approval number Pro00003833) using the method described previously with minor modifications^44^. Dithiothreitol (Gold Biotechnology) was added at a final concentration of 2.5 mmol to the saliva pool, and the mixture was incubated on ice for 30 min. The saliva was clarified by centrifugation at 23,000*g* for 1 h, followed by filtration through a 0.22-μm-pore-size membrane filter (Corning). Pooled saliva was stored frozen at −20 °C. Saliva from at least four donors was pooled to minimize the potential effects of donor variation. The ability of GAS strains to grow and persist in human saliva was evaluated as described previously^25^. Briefly, human saliva was collected from healthy volunteers and pooled as described above. GAS or SAL grown to mid-exponential growth phase in THY were pelleted, washed twice with sterile PBS, and stored in PBS at −80 °C till use. GAS and/or SAL were suspended in saliva, aliquots were removed at the indicated timepoints, serially diluted and plated in duplicate on either blood agar (BD Biosciences) or THY agar supplemented with erythromycin to enumerate GAS or SAL CFUs, respectively. The plates were incubated overnight, and colonies were counted to determine the number of CFU. All incubations were at 37 °C with 5% CO_2_. Each experiment was performed in triplicate on three separate occasions.

### Spent medium supplementation assay

To assess the presence of regulatory activity in the cell-free culture supernatant of SAL that activates SIP pathway in GAS, SAL was grown to mid-exponential phase of growth (*A*_600_ ≈ 2.0). Cells were removed by centrifugation and spent medium was prepared by filtering the cell-free culture supernatants through 0.22-µm membrane filter. The GAS sip* mutant grown to late-exponential growth phase (*A*_600_ = 1.0) was resuspended in the spent medium prepared from the SAL growth and incubated at 37 °C for 1 h. Transcript level analyses was performed by quantitative reverse transcription polymerase chain reaction (qRT–PCR) as described below.

### Synthetic peptide addition assay

Synthetic peptides of high purity (>90% purity) obtained from Peptide 2.0 were suspended in 100% DMSO to prepare a 10 mM stock solution. Stock solutions were aliquoted and stored at −20 °C until use. Working stocks were prepared by diluting the stock solution in 25% DMSO.

### Transwell co-cultivation experiments

GAS or SAL strains were grown to mid-exponential phase of growth, collected, washed with PBS and stored at −80 °C. Cells were diluted in THY or human saliva to 10^7^ CFUs ml^−1^. The peptide-producing and peptide-sensing strains are placed in the top and bottom chambers of a 24-well (Thermo Scientific) transwell, respectively. Cells were collected at the indicated timepoints and characterized for bacterial burden by CFU analyses and transcript level analyses by qRT–PCR.

### Transcript level analysis

GAS strains were grown to the indicated *A*_600_ and incubated with two volumes of RNAprotect (Qiagen) for 10 min at room temperature. RNA isolation and purification were performed with RNeasy kit (Qiagen). After quality control analysis, cDNA was synthesized from the purified RNA using Superscript III (Invitrogen) and Taqman qRT–PCR was performed with an ABI 7500 Fast System (Applied Biosystems). Comparison of transcript levels was performed by the ∆*C*_T_ method of analysis using *tufA* as the endogenous control gene^45,46^. The primers used for qRT–PCR are listed in Supplementary Table 2.

### Western immunoblot analysis of SpeB

Cells were grown to indicated growth phase and collected by centrifugation. The cell-free culture supernatant was prepared by filtration with 0.22 µM membrane, and the filtrate was concentrated twofold by speed-vac drying. Equal volumes of the samples were resolved on a 15% sodium dodecyl sulfate–polyacrylamide gel electrophoresis gel, transferred to a nitrocellulose membrane, and probed with polyclonal anti-SpeB rabbit antibodies. SpeB was detected with a secondary antibody conjugated with horseradish peroxidase and visualized by chemiluminescence using the SuperSignal West Pico Rabbit IgG detection kit (Thermo Scientific).

### Recombinant protein overexpression and purification

The coding regions of the full-length *ropB* gene of strain MGAS10870 were cloned into plasmids pET-28a. RopB overexpression and purification was carried out as described previously^33^. The coding region of *speB* of strain MGAS10870 without its secretion signal sequence and autoinhibiting pro-peptide (amino acids 146–398) was cloned into plasmid pET-28a. Site-directed mutagenesis was carried out to introduce serine substitution at active site residue C192 of SpeB. The mature SpeB protease was overexpressed in *E. coli* strain BL21(DE3). Cells were grown at 37 °C till the *A*_600_ reached 0.5 and induced with 0.5 mM isopropyl β-d-1-thiogalactopyranoside at 37 °C for 3 h. Cells were resuspended in buffer A (20 mM Tris–HCl pH 8.0, 0.2 M NaCl, 5% glycerol and 1 mM tris(2-carboxyethyl)phosphine) and lysed by a cell lyser (Microfluidics). The N-terminal hexa-histidine tagged SpeB was purified by affinity chromatography using a Ni-NTA agarose column. Finally, SpeB_M_ was purified by size exclusion chromatography with Superdex 75 G column. The protein was purified to >95% homogeneity, and the sequence identity of the purified SpeB_M_ was confirmed by MS-based identification of the N-terminal amino acids. The proteins concentrated to a final concentration of ~10–20 mg ml^−1^.

### FP assay

The interactions between RopB and synthetic NIP or SIP were analysed by fluorescence polarization (FP) assay as described previously^33,47^. The polarization (*P*) of fluorescein-labelled synthetic peptides increases as a function of protein binding. The millipolarization (*P* × 10^–3^) against protein concentration was plotted and equilibrium dissociation constants were determined. The effect of synthetic SIP or NIP on RopB–promoter interactions was assessed by FP assay using 5′-fluoresceinated oligoduplex containing RopB binding site. One nanomolar of fluorescein-labelled oligoduplex in binding buffer (20 mM Tris–HCl pH 8.5, 200 mM NaCl, 1 mM tris(2-carboxyethyl)phosphine and 25% DMSO) was titrated against increasing concentrations of purified RopB, and the resulting change in polarization was measured. Samples were excited at 490 nm, and emission was measured at 530 nm. The RopB-peptide-binding studies were performed in a peptide-binding buffer composed of 20 mM potassium phosphate pH 6.0, 75 mM NaCl, 2% DMSO, 1 mM ethylenediaminetetraacetic acid and 0.0005% Tween 20. All data were plotted using KaleidaGraph, and the resulting plots were fitted with the equation *P* = ((*P*_bound_ − *P*_free_)[protein]/(*K*_D_ + [protein])) + *P*_free_ (ref. ^48^), where *P* is the polarization measured at a given protein concentration, *P*_free_ is the initial polarization of the free ligand, *P*_bound_ is the maximum polarization of specifically bound ligand and [protein] is the protein concentration. Non-linear least squares analysis was used to determine *P*_bound_ and *K*_D_. The binding constant reported is the average value from at least three independent experimental measurements.

### SpeB protease activity assay

Analysis of SpeB protease activity was assessed by casein hydrolysis and zone of clearance on skimmed milk agar plates. GAS growth was stabbed on milk agar plates, and protease activity was analysed following overnight incubation at 37 °C.

### Site-directed mutagenesis of SpeB

The quick-change site-directed mutagenesis kit (Agilent) was used to introduce single amino acid substitutions within the *ropB* coding region in plasmid pET-28a–*ropB*, and mutations were verified by DNA sequencing. The primers used to introduce the substitutions are listed in Supplementary Table 2.

### SpeB protease activity against lantibiotics and salivabactin

The lantibiotics were extracted from ∆sarC-P mutant grown on a THY agar plate. Cells were scraped off the plate and subjected to freezing and thawing, and extracts containing lantibiotics were collected by centrifugation. Subsequently, the extracts were incubated with purified recombinant WT or C192S mature SpeB for 2 h before testing the extracts for anti-GAS activity. To test salivabactin sensitivity to SpeB protease activity, salivabactin dissolved in DMSO was added into 20 mM Tris–HCl (pH 7) with 50 ng µl^−1^ of purified rSpeB_M_ in a total volume of 100 µl. Incubation was carried out at 37 °C for 6 h. Then, the sample was treated with ethyl acetate. The organic phase extract was separated by centrifugation, evaporated, and finally resuspended in methanol and injected to LC–HRMS for detecting the relative concentration of salivabactin, as described above. As the negative controls, the heat-treated SpeB or a SpeB variant (C192S) was used for the same treatment of salivabactin, separately.

### Animal infection studies

All animal infection studies were performed according to protocols approved by the Houston Methodist Hospital Research Institute or Emory University Institutional Animal Care and Use Committee. These studies were carried out in strict accordance with the recommendations in the Guide for the Care and Use of Laboratory Animals, 8th edition.

### Salivabactin in vivo efficacy studies

GAS virulence in untreated and antibiotic-treated groups was assessed using i.m. mouse model of infection (approved protocol number IS00006169). For i.m. infection, ten female 3–4-week-old CD1 mice (Harlan Laboratories) per group were used. Mice were inoculated in the right hindlimb with 10^7^ CFUs of GAS. After 1 h of GAS infection, mice were treated intramuscularly with one of the following: PBS as mock treatment, DMSO, the carrier used to dissolve salivabactin, salivabactin at 6 mg kg^−1^ dose, and penicillin G at mg kg^−1^ dose. Mice were monitored for near mortality. Results were graphically displayed as a Kaplan–Meier survival curve and analysed using the log-rank test.

### Mouse vaginal colonization studies

The human-adapted streptococci, GAS and SAL, are poor colonizers of mouse oropharynx. Thus, the murine model of oropharyngeal GAS or SAL colonization has significant limitations in studying interspecies interactions in vivo. However, the murine vaginal colonization model offers an excellent alternative to study streptococcal mucosal colonization^29^. Both GAS and SAL can attach to vaginal epithelial cells and colonize mouse vaginal mucosa^12,29^. GAS can persist asymptomatically in mouse vaginal lumen for several weeks at high levels^29^. Furthermore, host immune responses elicited in response to pathogens are similar for upper respiratory tract and vaginal lumen^49,50^. Thus, we employed mouse vaginal model of streptococcal colonization to investigate the interspecies interactions between GAS and SAL. Mice were injected intraperitoneally with 0.5 mg of β-oestradiol 17-valerate (Sigma) in 0.1 ml sterile sesame oil to synchronize oestral cycles. One day after oestrogen treatment, inoculum prepared in PBS was placed in the vaginal lumen (approved protocol number IS00006169). Subsequently, vaginal lumen was swabbed and resuspended in PBS. Swab exudates were serially diluted and plated onto blood agar plate for GAS or THY agar containing erythromycin for SAL. After overnight incubation, bacterial burden was assessed by enumerating CFUs.

### Oropharyngeal infection studies

Seven- to 8-week-old WT C57BL/6 mice of both sexes (Jackson Laboratory) were used for experiments. Mouse groups were routinely inoculated intranasally with 10^8^ CFU GAS, SAL or eSAL slowly administered via micropipette in 10 μl PBS divided between nostrils and were allowed to aspirate the inoculum via the normal breathing process as previously^26^ (approved protocol number PROTO201800227). At indicated times, the mice were killed, and nasal-associated lymphoid tissues (NALTs) collected and homogenized in PBS for CFU enumeration, or in RNAlater-ICE (ThermoFisher) for expression analysis.

### ELISA to measure IL-1β levels

Homogenates were diluted in PBS and IL-1β quantified by enzyme-linked immunosorbent assay (ELISA) using the manufacturer’s protocol and recommendations for dilutions (DY401; R&D Biosciences).

### Statistical/data analysis

GraphPad Prism (version 10.0) was used for plot generation and statistical analyses. Exact *P* values are shown in the figure legend when significant except when the *P* values are <0.0001. No data were excluded from analyses. Multiple comparison Kruskal–Wallis analyses were used for experiments containing more than two groups, whereas Mann–Whitney test was used for experiments with two groups to assess statistical significance.

### Reporting summary

Further information on research design is available in the Nature Portfolio Reporting Summary linked to this article.

## Supplementary information

## Source data

## Data Availability

Data supporting the findings of this study are available in this article and its Supplementary Information files. Genomes of *S. salivarius* K12 and MGAS10870 were sequenced previously, and sequences are publicly available. Primers used in this study are provided in Supplementary Table 2. Metabolomics data related to salivabactin can be found in Supplementary Information. RNA-seq data have been deposited in the Gene Expression Omnibus (GEO) under accession code GSE247581. Minimum datasets that are necessary to interpret, verify and extend the research in the article are provided as source data with this paper.

## Code availability

No custom code was used for data analyses.
