# Supplementary material for: Engineered probiotic overcomes pathogen defences using signal interference and antibiotic production to treat infection in mice
Source: Nat Microbiol. 2024 Jan 16;9(2):502–13. doi: 10.1038/s41564-023-01583-9 (PMC10847043; doi:10.1038/s41564-023-01583-9)
Supplement: Supplementary file 1 — Supplementary Figures 1–38 and Tables 1 and 2. [file 41564_2023_1583_MOESM1_ESM.pdf]

nature microbiology

Article

https://doi.org/10.1038/s41564-023-01583-9

Engineered probiotic overcomes pathogen defences using signal interference and antibiotic production to treat infection in mice

In the format provided by the authors and unedited

Fig S1

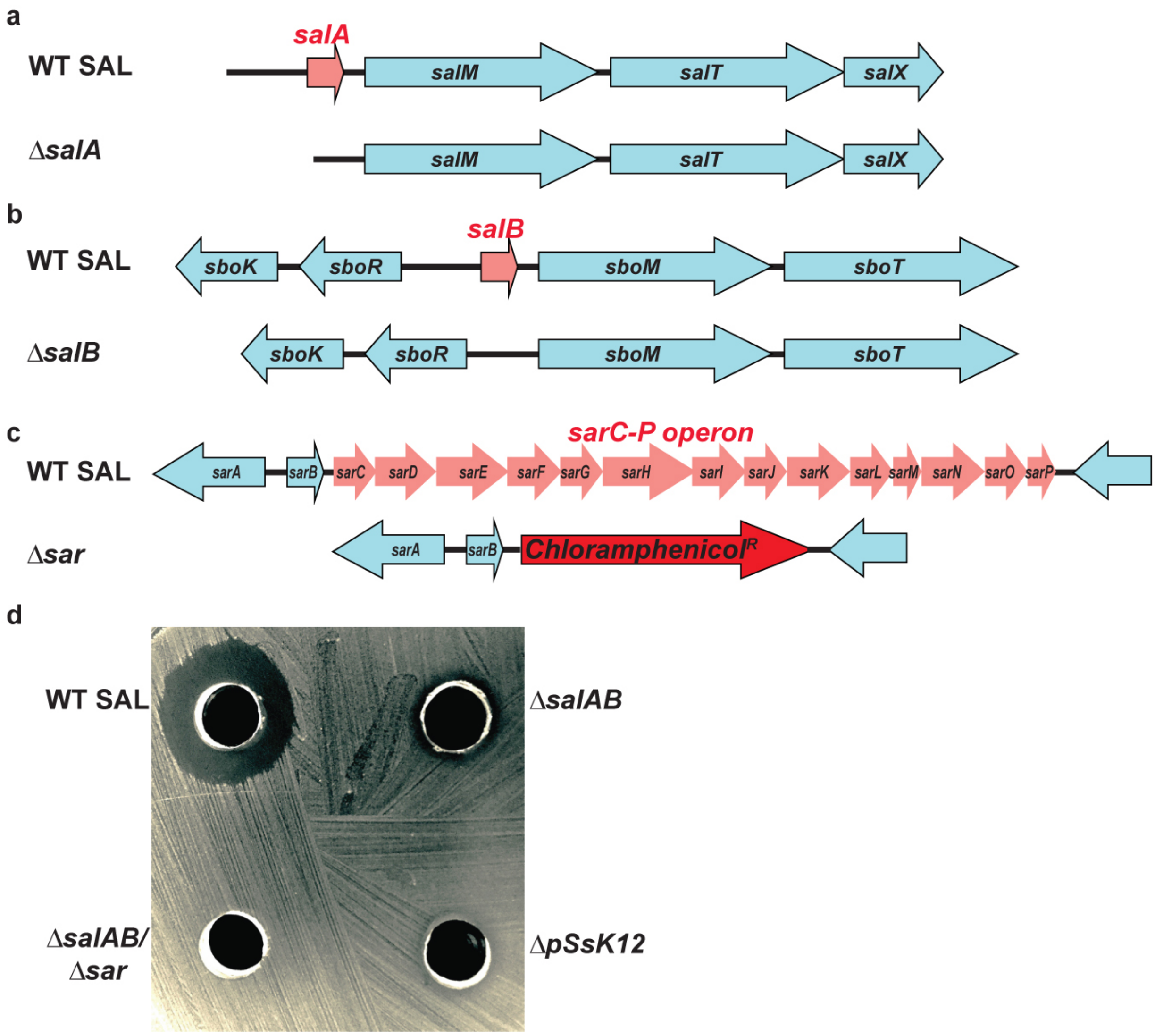

Figure S1. Schematics of the SAL mutant strains used to characterize antimicrobial activity. Organization of *salA* (**a**), *salB* (**b**), and *sarA-P* (**c**) gene regions in the megaplasmid of WT SAL (top panel) and their respective mutant strains (bottom panel) are shown. The *salA* and *salB* genes were inactivated by marker less deletion, whereas *sarC-P* deletion was achieved by replacing the entire *sar* operon with chloramphenicol resistance marker. The horizontal arrows indicate genes in the immediate vicinity of *salA*, *salB* and *sar* operon. The arrowheads indicate the predicted direction of the transcription of associated genes. **d,** A representative uncropped image of an agar plate showing the anti-GAS activity of indicated SAL strains. GAS lawn was grown on a Todd-Hewitt agar plate supplemented with yeast extract (THY). Cultures of WT SAL, the lantibiotic-deficient Δ*salAB* mutant, the lantibiotic and salivabactin-deficient (Δ*salAB/*Δ*sar*), and the megaplasmid cured (Δ*pSsK12*) strains were grown to late-exponential phase of growth (A_{600} ~ 2.0) and 20 µl of bacterial

growth was spotted in wells on the agar plate containing GAS lawn. The zone of inhibition was visualized after 16 h incubation at 37˚C.

Fig S2

Protein	Locus tag	Deduced role
SarA	*RSSL_00023*	Transcriptional regulator
SarB	N/A	Leaderless peptide
SarC	*RSSL_00024*	Putative DNA-binding protein
SarD	*RSSL_00025*	NRPS-PKS
SarE	*RSSL_00026*	NPRS-PKS
SarF	*RSSL_00027*	Dehydratase
SarG	*RSSL_00028*	Ketoreductase
SarH	*RSSL_00029*	PKS
SarI	*RSSL_00030*	Thioesterase
SarJ	*RSSL_00031*	Acyltransferase
SarK	*RSSL_00032*	4'-phosphopantetheinyltransferase
SarL	*RSSL_00033*	Putative transposase
SarM	*RSSL_00034*	Putative transposase
SarN	*RSSL_00035*	Putative integrase/recombinase
SarO	*RSSL_00036*	Hypothetical protein
SarP	*RSSL_00037*	Hypothetical protein

Figure S2. **Deduced roles of open reading frames in the *sar* BGC.**

Fig S3

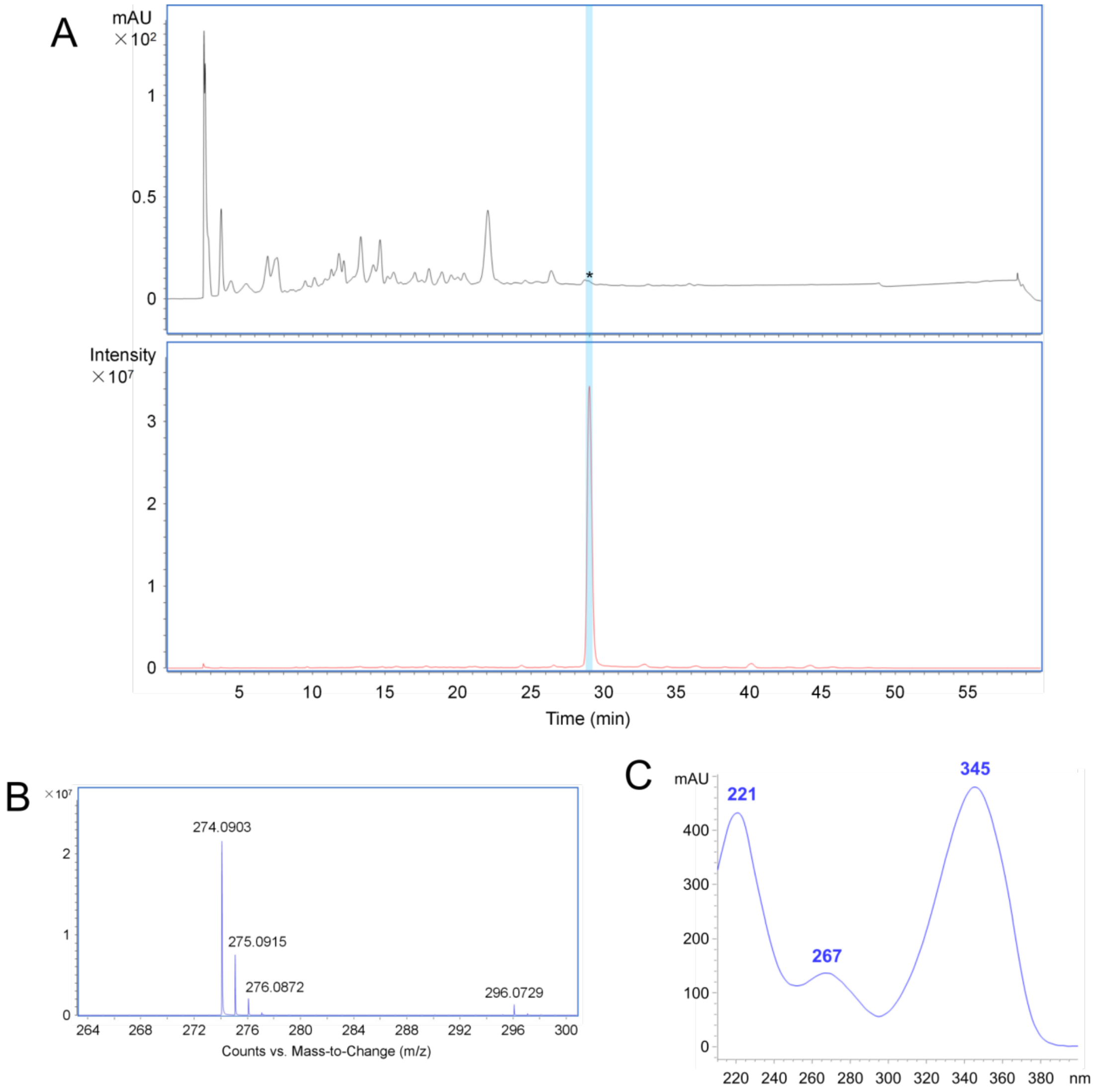

Figure S3. **HRMS and UV-vis analysis of salivabactin**. **a**, HPLC analysis of the extract from SAL. The chromatogram was monitored at 345 nm (upper panel). LC-HRMS extracted ion chromatogram trace of the same sample. EIC+ = 274. ±0.01 (bottom panel). **b,** The high-resolution ESI-MS of salivabactin; its molecular formula was determined as $C_{15}H_{15}NO_2S$ on the basis of its protonated molecular ion peak at *m*/*z* 274.0903 (calculated, 274.0896). **c,** UV spectrum of salivabactin.

Fig S4

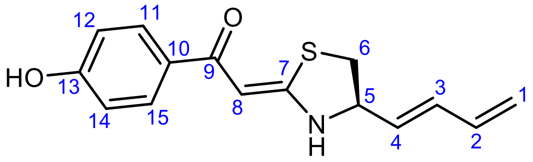

C	δ_C	δ_H (*J* in Hz)	COSY	HMBC (^{1}H to ^{13}C)
1	118.5	a 5.26 d (17.1)	1b, 2	3
		b 5.15 d (10.1)	1a, 2	3
2	136.1	6.39 ddd (17.1, 10.1, 7.6)	1a, 1b, 3	3
3	132.4	6.27 m	2, 4	1, 2, 5
4	131.9	5.86 dd (15.2, 6.9)	3, 5	2, 5, 6
5	62.6	4.80 ddd (7.3, 6.9, 6.0)	4, 6a, 6b	3, 6, 7
6	33.7	a 3.49 dd (10.9, 7.3)	5, 6b	4, 5, 7
		b 3.09 dd (10.9, 6.0)	5, 6a	4, 5, 7
7	167.0	-		
8	85.6	5.97 s		7, 9
9	184.1	-		
10	130.2	-		
11	129.1	7.73 d (8.4)	12	9, 13, 15
12	115.0	6.77 d (8.4)	11	10, 13, 14
13	160.3	-		
14	115.0	6.77 d (8.4)	15	10, 12, 13
15	129.1	7.73 d (8.4)	14	9, 11, 13

Figure S4. Structural characterization data of salivabactin A (1a).

Fig S5

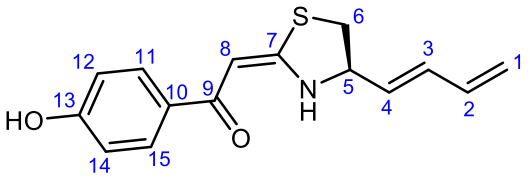

C	δ_C	**δ_H (*J* in Hz)**	**COSY**	**HMBC (^{1}H to ^{13}C)**
1	118.5	a 5.25 d (17.1)	1b, 2	3
		b 5.13 d (10.1)	1a, 2	3
2	136.2	6.39 ddd (17.1, 10.1, 7.6)	1a, 1b, 3	3
3	132.3	6.27 m	2, 4	1, 2, 5
4	132.7	5.80 dd (15.2, 7.0)	3, 5	2, 5, 6
5	59.8	4.46 ddd (7.5, 7.0, 6.9)	4, 6a, 6b	3, 6, 7
6	34.6	a 3.25 dd (11.0, 7.5)	5, 6b	4, 5, 7
		b 2.85 dd (11.0, 6.9)	5, 6a	4, 5, 7
7	167.9	-		
8	87.0	6.30 s		7, 9
9	183.4	-		
10	131.0	-		
11	128.7	7.62 d (8.4)	12	9, 13, 15
12	114.9	6.78 d (8.4)	11	10, 13, 14
13	160.0	-		
14	114.9	6.78 d (8.4)	15	10, 12, 13
15	128.7	7.62 d (8.4)	14	9, 11, 13
NH		8.46 s		5, 6, 7

Figure S5. Structural characterization data of salivabactin B (1b).

Fig S6

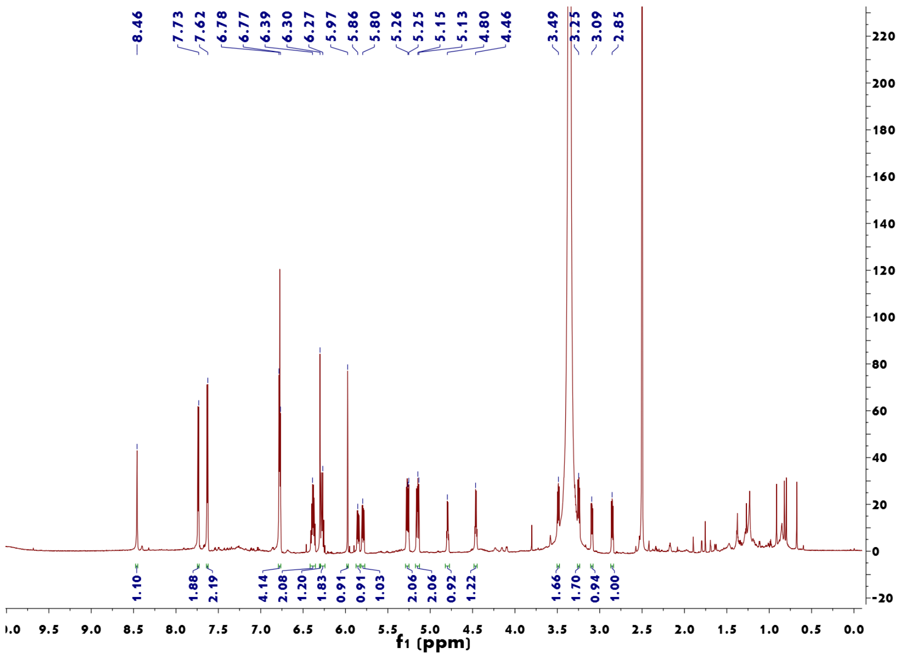

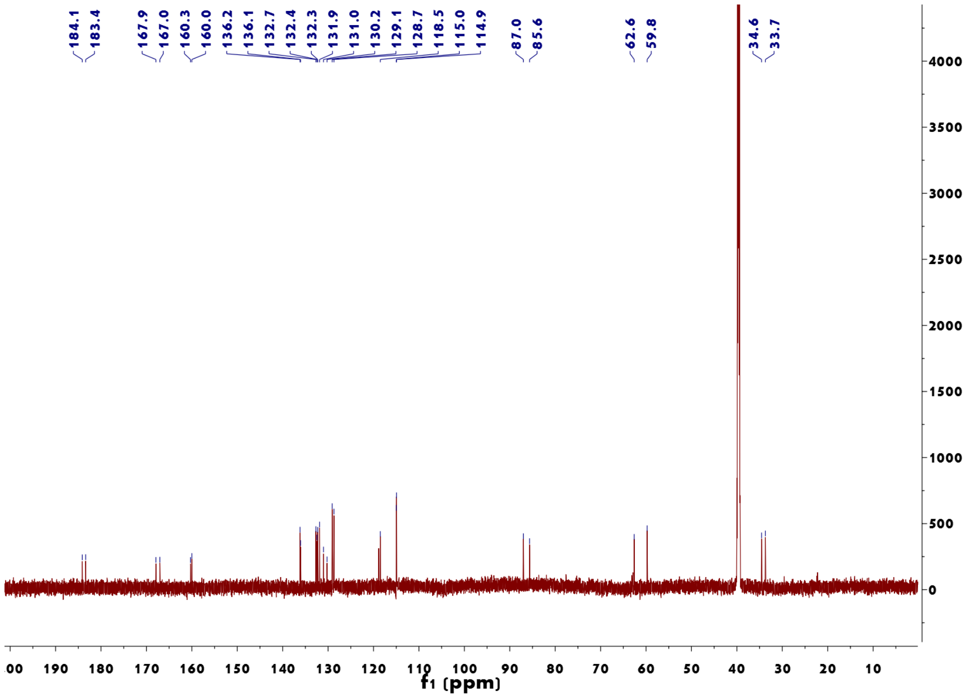

Figure S6. **NMR spectra of salivabactin.** ^{1}H NMR spectrum (top) (recorded in DMSO-d_6 at 900 MHz) and ^{13}C NMR spectrum (bottom) (recorded in DMSO-d_6 at 225 MHz).

Fig S7

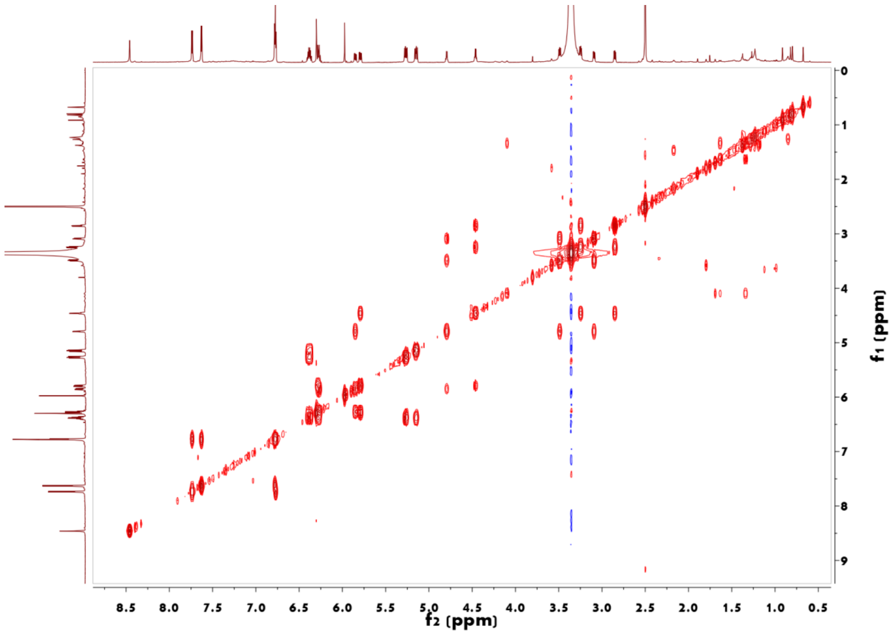

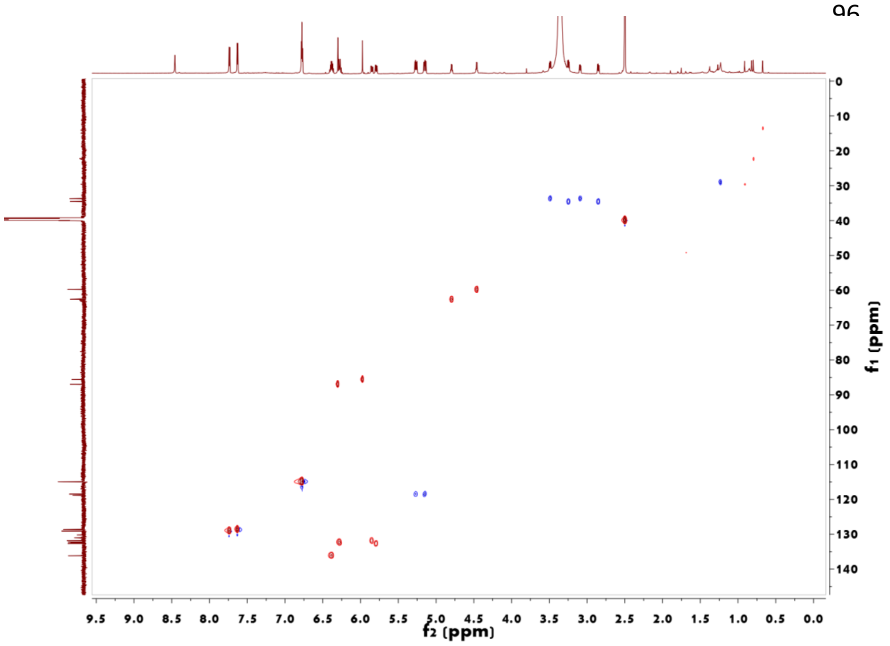

Figure S7. **NMR spectra of salivabactin.** ^{1}H–^{1}H COSY spectrum (top) (recorded in DMSO-d_6 at 900 MHz) and ^{1}H–^{13}C HSQC spectrum (bottom) (recorded in DMSO-d_6 at 900 MHz).

Fig S8

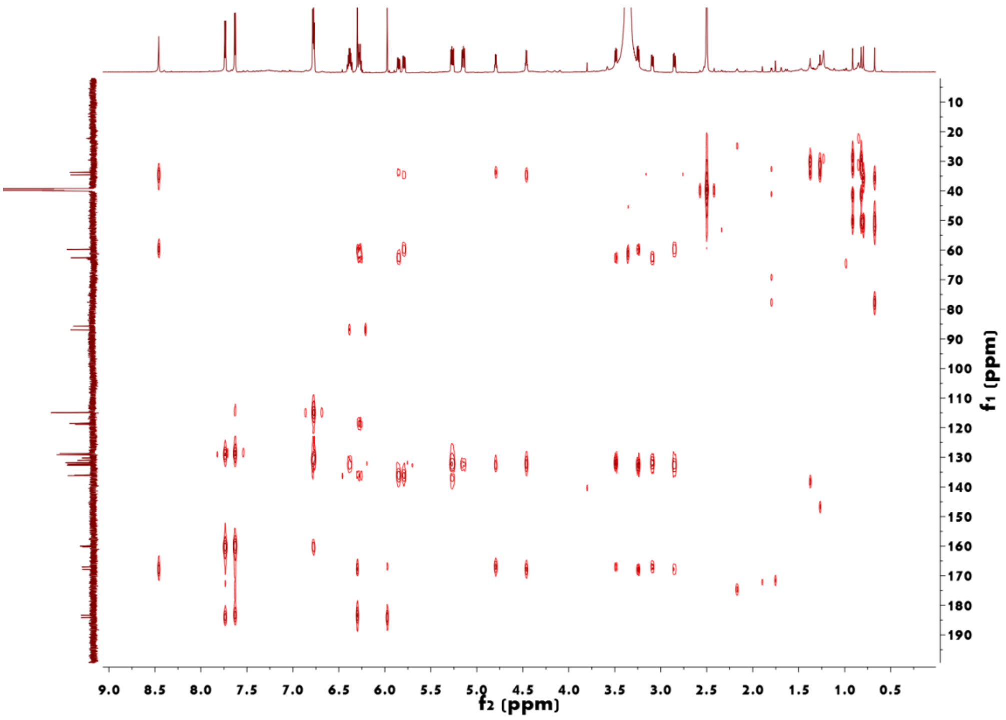

Figure S8. NMR spectra of salivabactin. ^{1}H–^{13}C HMBC spectrum (recorded in DMSO-d_6 at 900 MHz).

Supplementary note

Salivabactin A/B, a pair of geometric isomers, were isolated concomitantly as white amorphous solids. The positive ion HRESIMS data revealed a peak for a protonated molecular ion at *m/z* 274.0943 and for a sodium adduct ion at *m/z* 296.0729, both corresponding to a molecular formula of $C_{15}H_{15}O_2NS$. The isomers' ^{1}H NMR and HSQC spectra displayed signals for one hydrogen group (δ_H 8.46, NH of salivabactin B), eight aromatic methines of two individual AA'BB' *para*-aromatic spin systems [δ_H 7.73, H-11 and H-15 of salivabactin B (2H); δ_H 7.62, H-11 and H-15 of salivabactin A (2H); δ_H 6.78, H-12 and H-14 of salivabactin B (2H) and δ_H 6.77, H-12 and H-14 of salivabactin A (2H)], eight vinyl methines [δ_H 6.39, H-2 of salivabactin A and B (2H); δ_H 6.30, H-8 of salivabactin B (1H); δ_H 6.27, H-3 of salivabactin A and B (2H); δ_H 5.97, H-8 of salivabactin A (1H); δ_H 5.86, H-4 of salivabactin A (1H) and δ_H 5.80, H-4 of salivabactin B (1H)], two vinyl methylenes [δ_H 5.26, H-1a of salivabactin A (1H); δ_H 5.25, H-1a of salivabactin B (1H); δ_H 5.15, H-1b of salivabactin A (1H) and δ_H 5.13, H-1b of salivabactin B (1H)], two heteromethines [δ_H 4.80, H-5 of salivabactin A (1H) and δ_H 4.46, H-5 of salivabactin B (1H)], and two heteromethylenes [δ_H 3.49, H-6a of salivabactin A (1H); δ_H 3.25, H-6a of salivabactin B (1H); δ_H 3.09, H-6b of salivabactin A (1H) and δ_H 2.85, H-6b of salivabactin B (1H)]. Furthermore, the ^{13}C NMR and HSQC spectra

showed signals for two ketones (δ_C 184.1, C-9 of salivabactin A; δ_C 183.4, C-9 of salivabactin B), four hetero bearing carbons (δ_C 167.9, C-7 of salivabactin B; δ_C 167.0, C-7 of salivabactin A; δ_C 160.4, C-13 of salivabactin A; δ_C 160.0, C-13 of salivabactin B), sixteen methine sp^2 carbons (δ_C 136.2, C-2 of salivabactin B; δ_C 136.1, C-2 of salivabactin A; δ_C 132.7, C-4 of salivabactin B; δ_C 132.4, C-3 of salivabactin A; δ_C 132.3, C-3 of salivabactin B; δ_C 131.9, C-4 of salivabactin A; δ_C 129.1, C-11/C-15 of salivabactin A; δ_C 128.7, C-11/C-15 of salivabactin B; δ_C 115.0, C-12/C-14 of salivabactin A; δ_C 114.9, C-12/C-14 of salivabactin B; δ_C 87.0, C-8 of salivabactin B; δ_C 85.5, C-8 of salivabactin A), two normal quaternary sp^2 carbons (δ_C 131.0, C-10 of salivabactin B; δ_C 130.2, C-10 of salivabactin A), two methylene sp^2 carbons (δ_C 118.5, C-1 of salivabactin A and B), two hetero bearing methine sp^3carbons (δ_C 62.6, C-5 of salivabactin A; δ_C 59.8, C-5 of salivabactin B), and two hetero bearing methylene sp^3carbons (δ_C 34.6, C-6 of salivabactin B; δ_C 33.7, C-6 of salivabactin A).

The presence of two independent terminal diene units was proposed by their proton-proton coupling constants and supported by their COSY correlations between H-1a/1b and H-2, H-2 and H-3, and H-3 and H-4. The presence of two respective thiazolidin-ylidene sections was indicated by each compound's COSY correlation between H-5 and H-6a/6b, as well as ^3J-HMBC correlations between H-5/H-6a/6b and C-7. The nitrogen bearing methine of each thiazolidin-ylidene was specifically connected to each terminal diene through the COSY correlation between H-4 and H-5, along with ^3J-HMBC correlations between H-5 and C-3, and H-6a/6b and C-4. The ylidene carbon of each five member ring was belonged to an α,β-unsaturated ketone component which could be documented by ^2J-HMBC correlations between H-8 and C-7/C-9. The other side of each ketone was linked to an AA'BB' *para*-aromatic spin system, based on ^3J-HMBC correlations between H-11/H-15 and C-9. The *para*-substitution of each AA'BB' *p*-aromatic spin system should be a hydroxyl group due to the remaining unassigned atoms from the formula and typical oxygen bearing aromatic carbon chemical shift of C-13.

After detailed analysis of NMR data, a pair of geometric isomers were figured out through paired proton and carbon signals except the enamine proton signal. They were named as salivabactin B whose configuration of C-7 C-8 double bond was assigned as *E*, and salivabactin A that was assigned as *Z*. These two co-isolated isomers could be distinguished by the unpaired enamine proton signal (δ_H 8.46), because when the C-7 C-8 double bond was *E* configuration, the enamine proton could generate an intramolecular H-bond with ketone to present a particular signal in the ^{1}H NMR spectrum, while *Z* configuration would not show this signal. In addition, the absolute configuration of C-5 could be suggested as *R* which was relied on the biosynthesis mechanism.

Fig S9

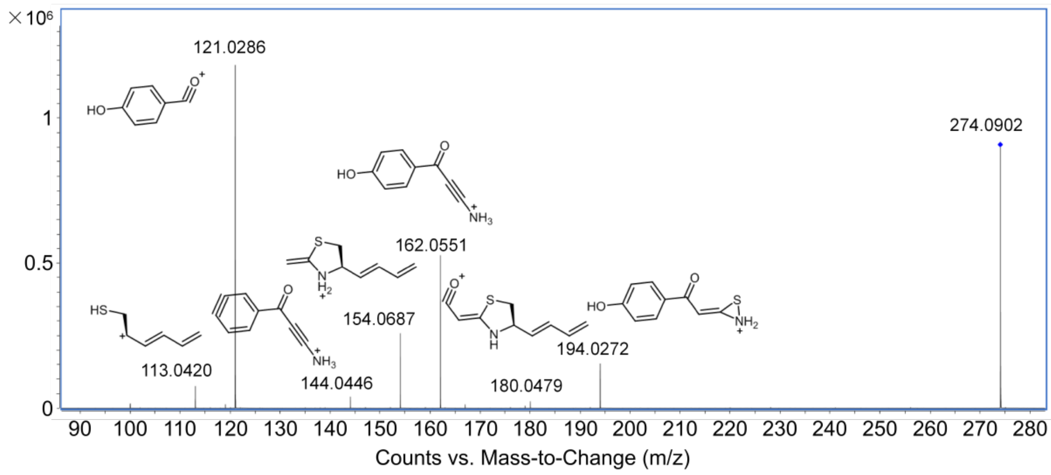

Fragmentation $[M+H]^+$	Obs. mass	Calc. mass	Δppm
F1	113.0420	113.0419	0.88
F2	121.0286	121.0284	1.65
F3	144.0446	144.0444	1.39
F4	154.0687	154.0685	1.30
F5	162.0551	162.0550	0.62
F6	180.0479	180.0478	0.56
F7	194.0272	194.0270	1.03

Figure S9. **HR-MS/MS fragmentation pattern of salivabactin and the major fragmentation species from the measurement.** Fragmentation was acquired with collision energy of 15 V. Obs. = observed; Calc. = calculated.

Fig S10

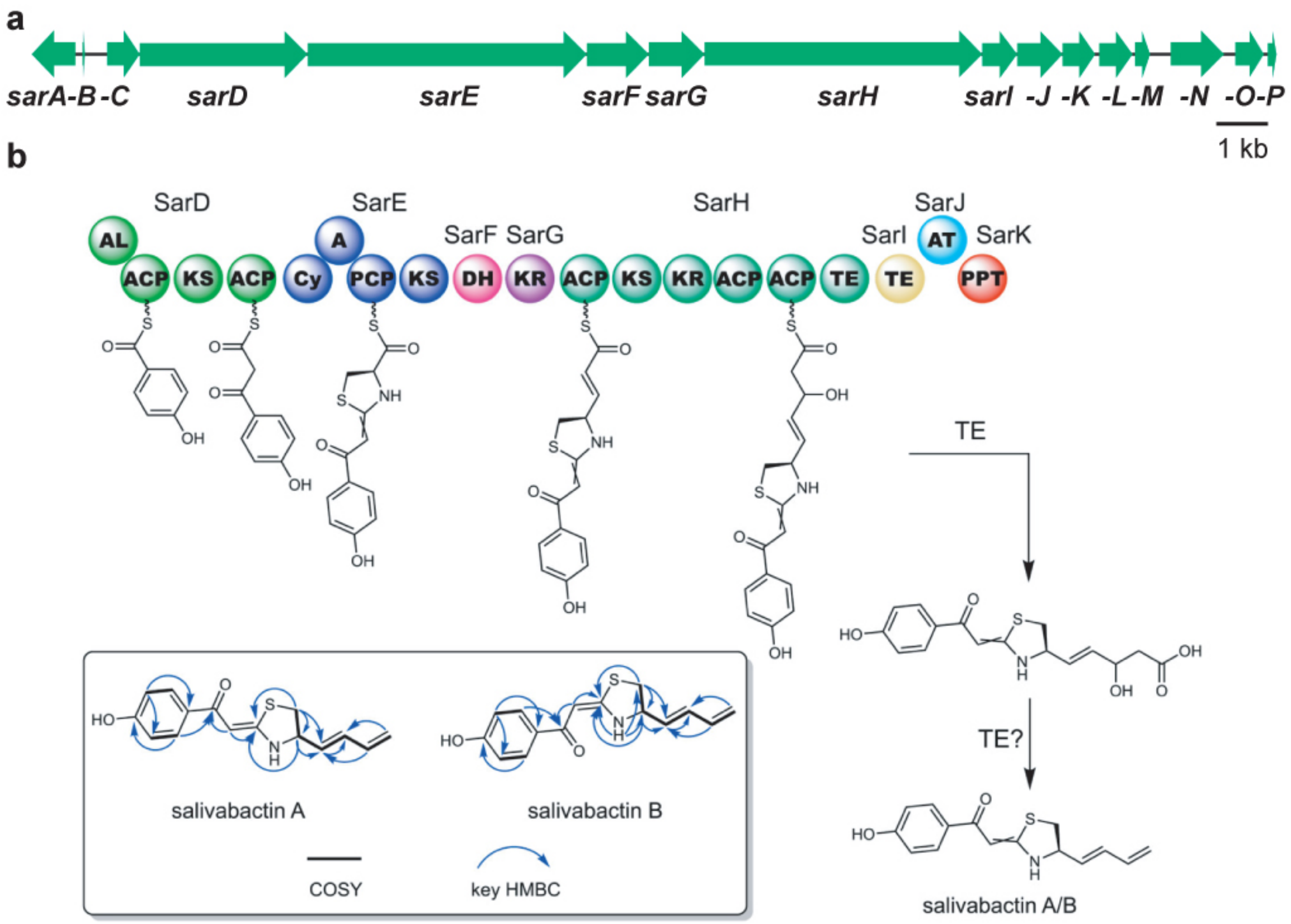

Figure S10. Proposed biosynthetic pathway for salivabactin. a, Organization of the *sar* BGC. **b**, Proposed biosynthetic pathway for salivabactin. AL: acyl-ACP ligase; ACP: acyl-carrier protein; KS, ketosynthase; Cy, cyclization; A, adenylation; PCP: peptidyl-carrier protein; DH, dehydratase; KR, ketoreductase; TE, thioesterase; AT, acyltransferase; PPT, 4'-phosphopantetheinyl transferase.

Supplementary note

A direct responsibility of *sar* for salivabactin production could be further confirmed by assessing the predicted activity of the *sar*-encoded enzymes in salivabactin biosynthesis. Specifically, *sarD-J* are predicted to encode the core *trans*-acyltransferase (AT) polyketide synthase/non-ribosomal peptide synthetase (PKS/NRPS) assembly line to generate the scaffold of salivabactin (**Fig. S10**). SarD initiates chain assembly by activating and loading *p*-hydroxybenzoic acid (*p*-HBA) followed by one round of PKS extension using malonyl-CoA. Upon activation of L-Cys by the adenylation (A) domain of SarE, the heterocyclization (Cy) domain of SarE promotes subsequent condensation and cyclodehydration to generate a five-membered heterocyclic ring. Notably, instead of a typical thiazoline ring, thiazolidine-ene is formed with a mixed configuration, possibly due to a prior unreduced ketide unit

[1,2]. The highly discrete machinery of SarE, G, F and H catalyze the next two rounds of PKS extension likely by releasing a β-hydroxyl acid intermediate that undergoes decarboxylation and dehydration to yield salivabactin, although the exact chain-releasing mechanism remains obscure. The proposed biosynthetic pathway of salivabactin was supported by stable isotope feeding results using [1-$^{13}C_1$]acetate or [1,2-$^{13}C_2$]acetate which showed the expected polyketide labeling patterns (**Fig. S11**).

Figure S11

F

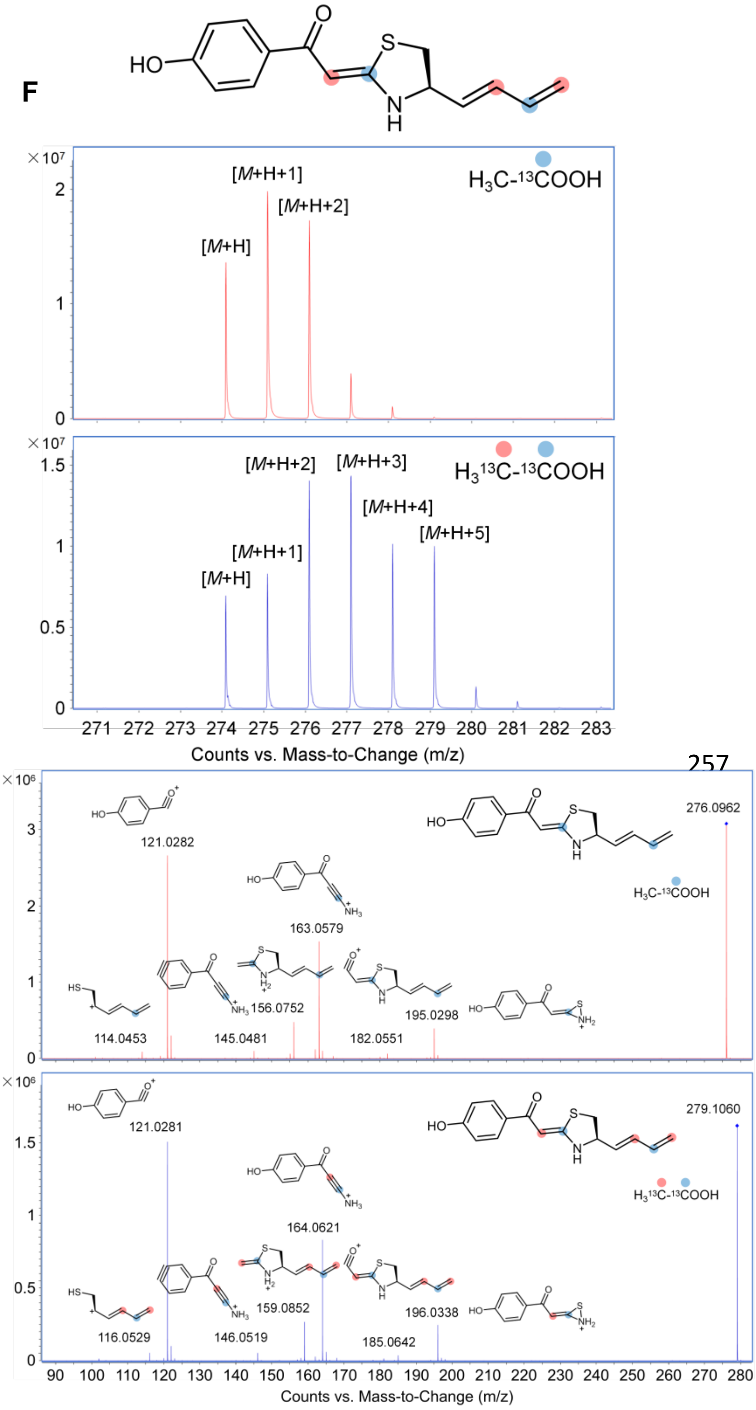

Figure S11. **Biosynthesis of labeled salivabactin upon precursor feeding.** Top panels showing the high-resolution ESI-MS of salivabactin after feeding sodium [1-$^{13}C_1$] acetate or sodium [1,2-$^{13}C_2$] acetate, respectively. Bottom panels showing the tandem MS-based measurements displaying that the feeding of sodium [1-$^{13}C_1$] acetate resulted in the expected enrichments in carbon atoms C-2 and C-7; and the feeding of sodium [1,2-$^{13}C_2$] acetate resulted in the expected enrichments in carbon atoms C-1, C-2, C-3, C-7 and C-8.

Figure S12

a

Species and Strain	Salivabactin MIC (µg/ml)
**Gram-positive pathogens**	
Streptococcus pyogenes MGAS10870	2
S. agalactiae ATCC49447	2
S. pneumoniae ATCC49619	2
S. mutans ATCC25175	2
S. dysgalactiae ATCC12394	2
L. monocytogenes ATCC19115	2
Staphylococcus aureus ATCC33591	2
Enterococcus faecalis ATCC51299	2
**Gram-negative pathogens**	
Escherichia coli ATCC25922	>8
Klebsiella pneumoniae ATCC700603	>8
Pseudomonas aeruginosa ATCC51299	>8

b

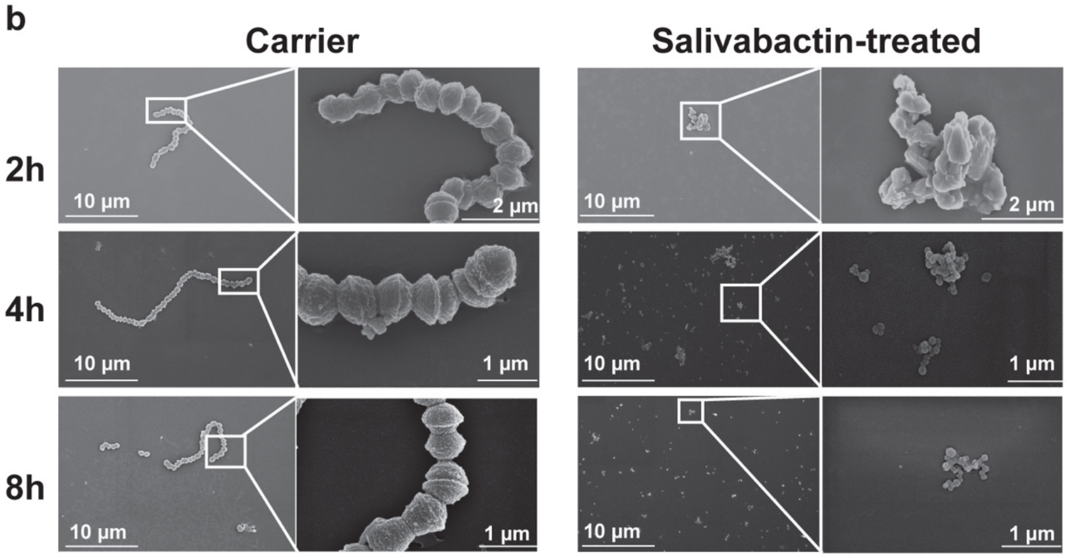

Figure S12. Salivabactin affects bacterial cell integrity. a, Minimum inhibitory concentration (MIC) of salivabactin against bacterial pathogens. **b,** GAS (10^5 CFUs/ml) was incubated either with carrier (DMSO) (left) or 2X MIC of salivabactin (right). Cells were collected at the indicated time points post incubation and visualized by scanning electron microscopy. Three independent experiments were performed and representative images are shown.

Figure S13

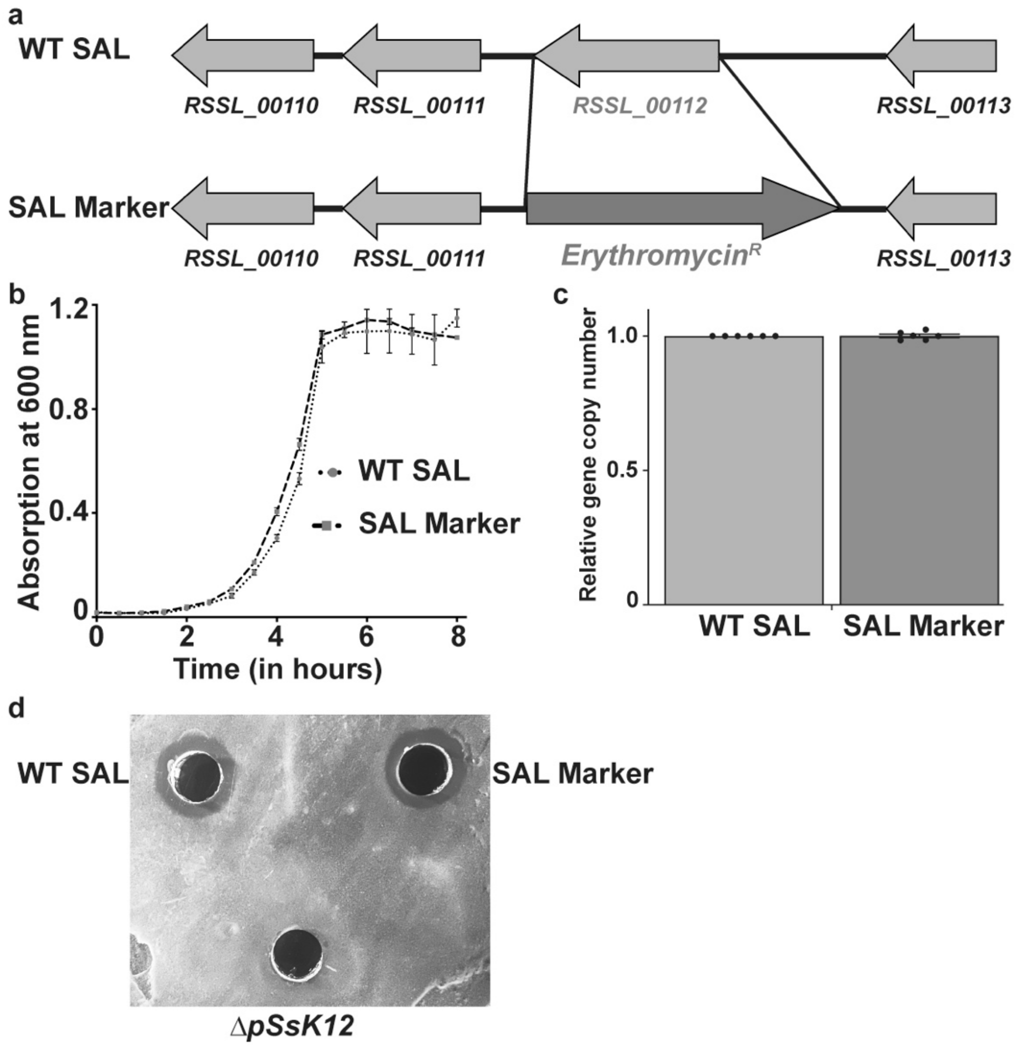

Figure S13. Schematics of marker strain used in the co-infection studies. For isolation and precise assessment of SAL levels during dual species competition studies, an erythromycin resistance marker (Erm^R) was introduced at locus RSSL_00112 (**a**). The SAL marker strain was used for co-cultivation studies with GAS *in vitro, ex vivo and in vivo* instead of unmodified WT SAL. The introduction of Erm^R did not affect the growth kinetics (**b**), megaplasmid copy number (**c**), and antimicrobial activity (**d**) of the SAL marker strain. We used RNAse-treated cell lysates from either WT SAL or SAL marker to deduce the gene copy number of RSSL_00025 encoded in the megaplasmid, which was used to compare the megaplasmid copy number in the two strains. Growth experiments in panel **b**, and gene copy number estimation assays in panel **c** were derived from three independent biological replicates that were analyzed in duplicates. In both panels **b** and **c**, data were presented as mean ± s.e.m. and no statistically significant differences were observed between WT SAL and SAL marker strains.

Fig S14

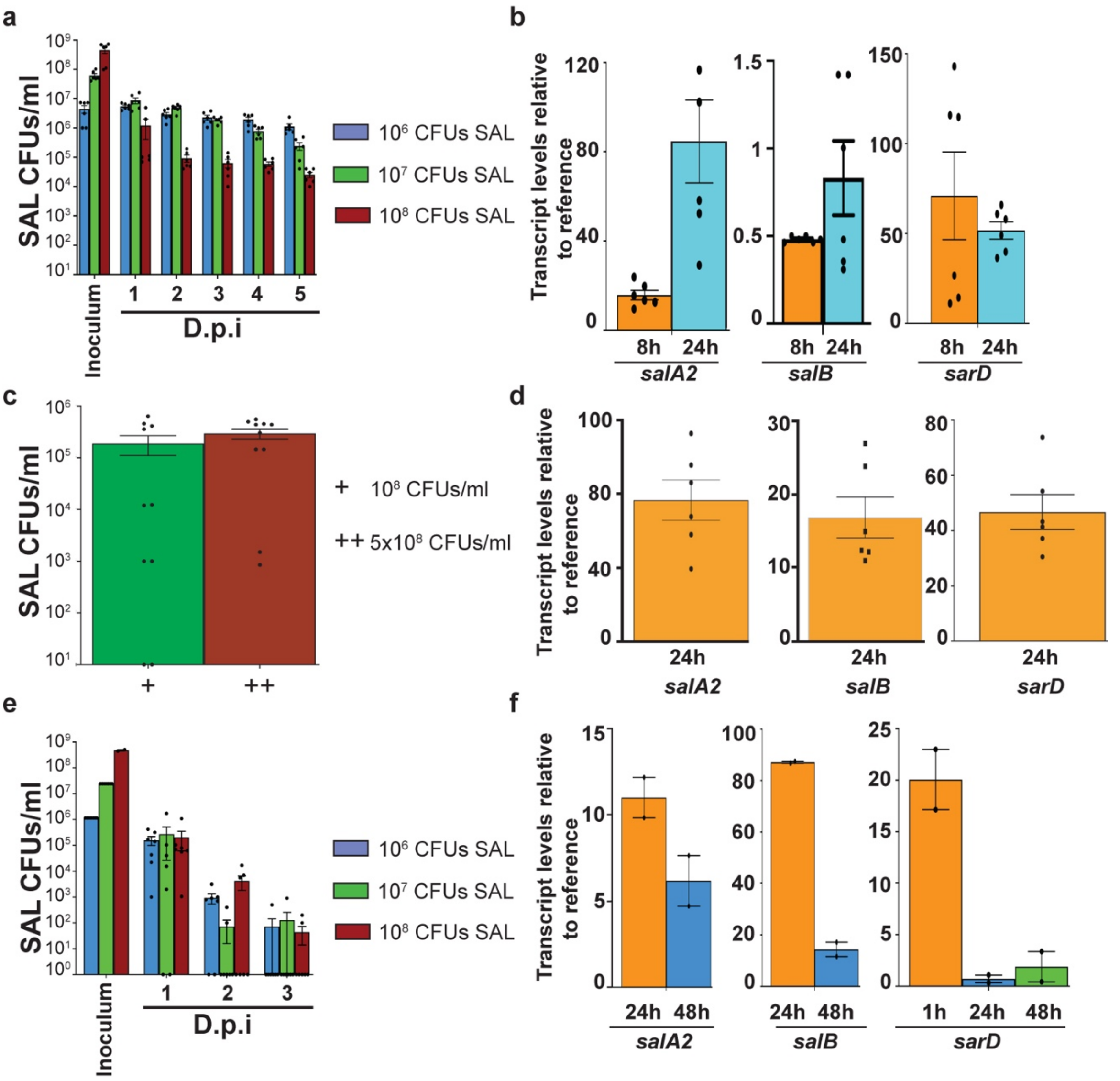

Figure S14. SAL produce antimicrobials during co-cultivation with GAS *ex vivo* and *in vivo*. SAL was grown in the presence of GAS *ex vivo* in sterile human saliva. Samples were collected at the indicated time points (days post infection, D.p.i) and SAL (**a**) levels were assessed by enumerating colony-forming units (CFUs) per ml of saliva. **b**, Transcript levels of salivaricin A (*salA2*), salivaricin B (*salB*), and *sarD* during dual species growth in human saliva as assessed by qRT-PCR are shown. **c**, SAL were injected intranasally with 10^8 CFUs of GAS in mice (*n=10* per group). Samples were collected at 1 d.p.i and SAL (**c**) levels were assessed by enumerating CFUs per ml of swab eluate. **d**, Transcript levels of *salA2*, *salB*, and *sarD* during co-colonization in mouse nasopharynx as assessed by qRT-PCR. Individual samples from each group (*n=6* per group) were used for RNA extraction and transcript level analyses. Indicated doses of SAL were injected intravaginally in mice (*n=5* per group) and 24 h later, mice

were inoculated intravaginally with 10^3 CFUs of GAS. Samples were collected at the indicated time points and SAL (**e**) levels were assessed by enumerating CFUs per ml of swab eluate. **f**) Transcript levels of *salA2*, *salB*, and *sarD* during co-colonization in mouse vaginal lumen as assessed by qRT-PCR. Pooled samples from each group (*n*=5 per group) were used for RNA extraction and transcript level analyses. Samples were analyzed in duplicate. Transcript levels of respective genes in SAL grown in laboratory medium to early exponential phase of growth (A_{600} ~ 0.5) were used as a reference. Fold change differences in transcript levels relative to reference (reference transcript level=1) is shown. The reference growth was performed in 3 independent occasions and analyzed in duplicate. Bars represent mean ± s.e.m.

Fig S15

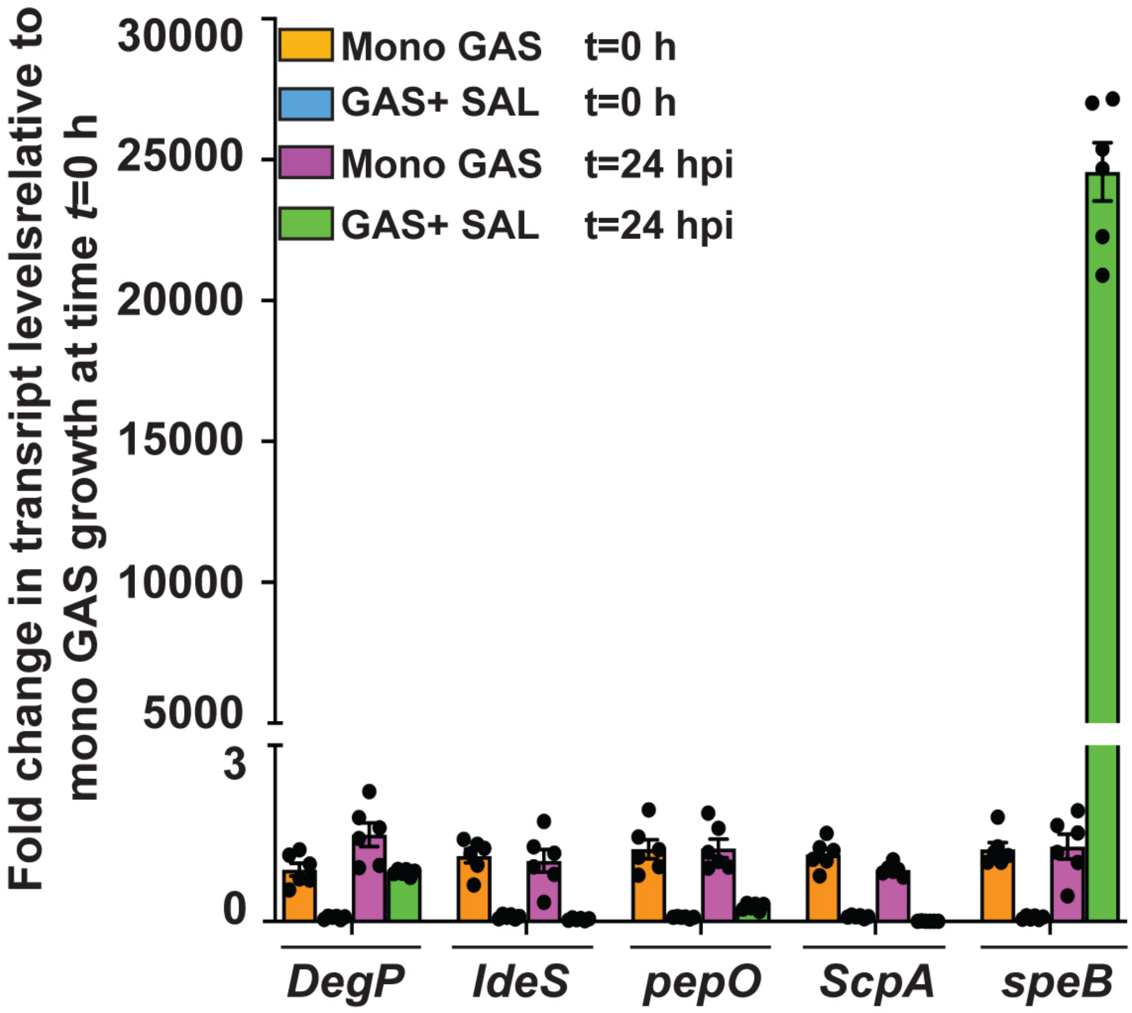

Figure S15. **Expression of *speB* encoding secreted GAS cysteine protease is specifically upregulated during co-cultivation with SAL in human saliva.** GAS was grown in the absence (Mono GAS) or presence of 10^7 CFUs/ml of SAL (GAS+SAL) in human saliva *ex vivo*. Samples were collected at the indicated time points and assessed for the expression of genes encoding GAS extracellular proteases by qRT-PCR. Experiment was performed as three biological replicates and analyzed in duplicate. Data graphed were mean ± s.e.m.

Fig S16

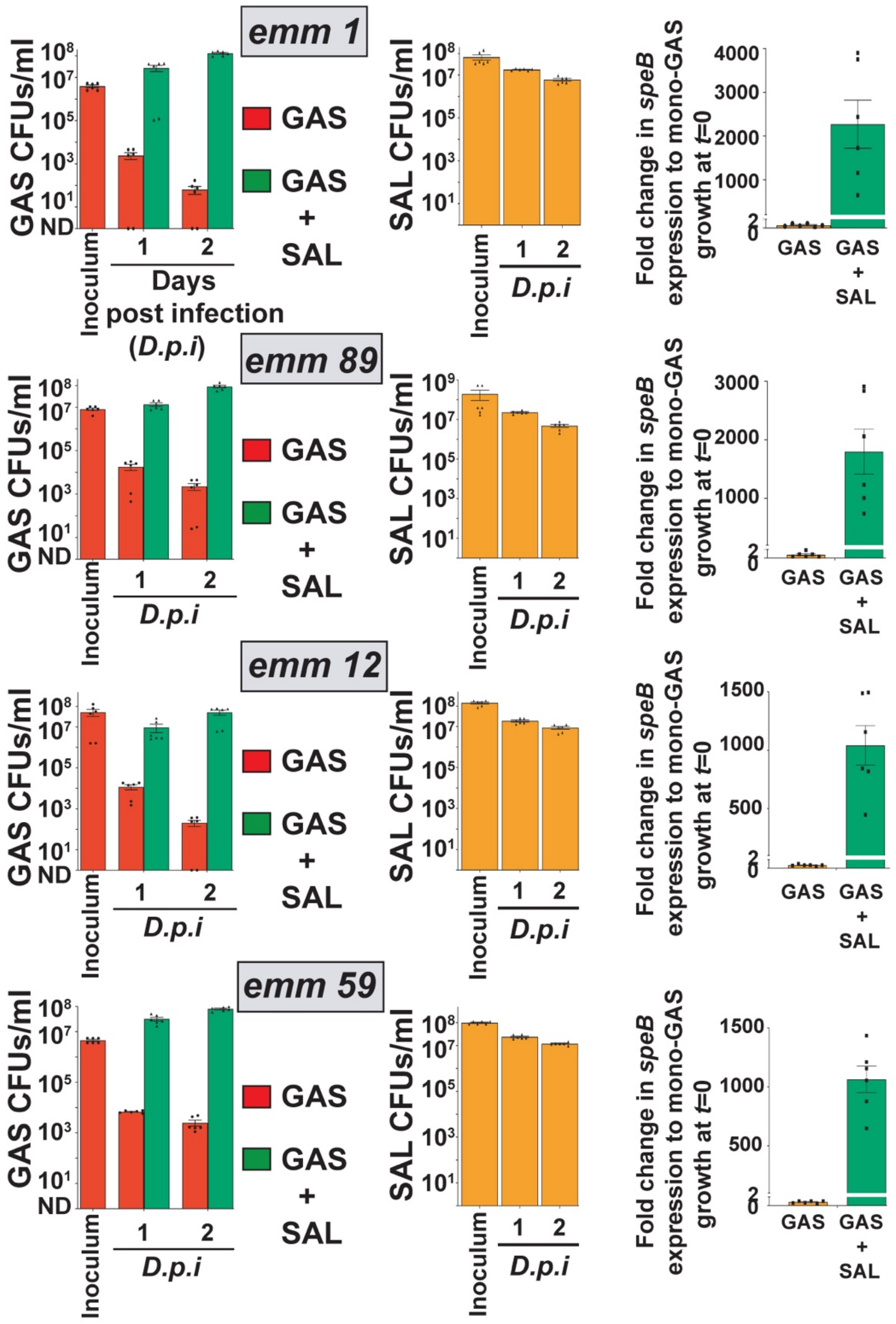

Figure S16. SAL K12-GAS interactions are not strain specific. GAS strains belong to 4 distinct *emm* serotypes, *emm1, emm89, emm12*, and *emm59*, were grown in the absence (GAS) or presence of SAL (GAS+SAL) in human saliva *ex vivo*. Samples were collected at either 1 or 2 days post infection and bacterial burden was assessed

by enumerating colony-forming units (CFUs) per ml of saliva. GAS CFUs (left) and SAL CFUs (center) are shown. GAS was grown in the absence (GAS) or presence of 10^7 CFUs/ml of SAL (GAS+SAL) in human saliva *ex vivo*. Samples were collected at the 24 hpi and assessed for *speB* expression by qRT-PCR (right). Experiment was performed as three biological replicates and analyzed in duplicate. Data graphed were mean ± s.e.m.

Fig S17

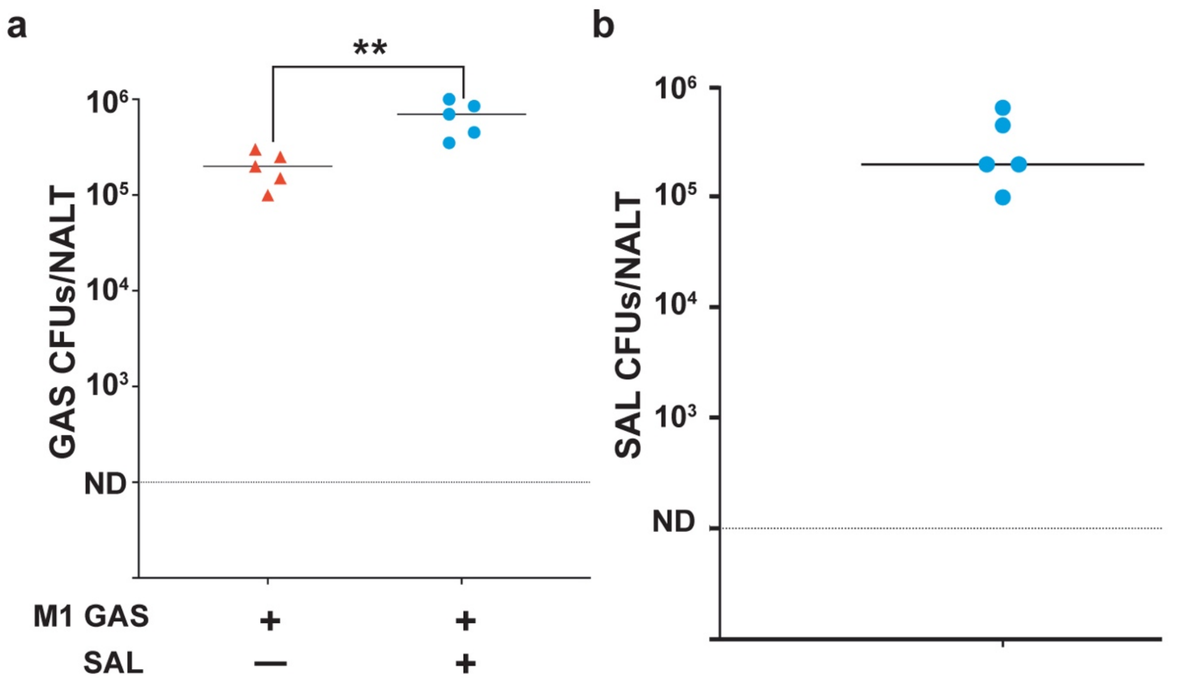

Figure S17. C57Bl/6 mice were inoculated intranasally with 10^8 CFUs of GAS and/or SAL. Samples were collected at 24 h post-infection and CFUs of GAS (**a**) and SAL (**b**) were enumerated. Data are pooled from two independent experiments and are presented as the median. (*n*=5). *P* values were calculated by two-tailed Mann-Whitney test. **, *P* = 0.008.

Fig S18

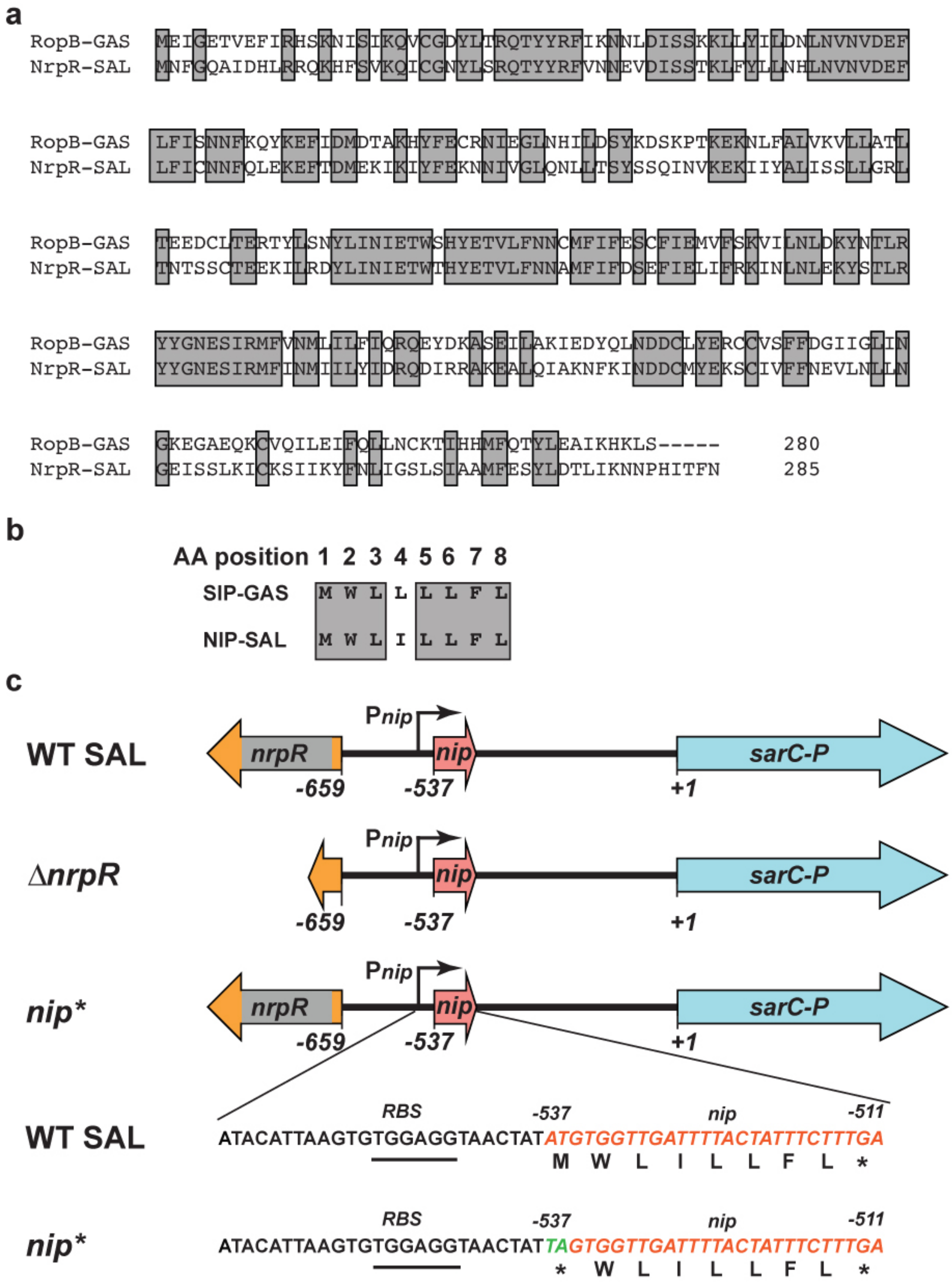

Figure S18. **Identification of NrpR-NIP signaling pathway in SAL that controls *sar-BGC* expression. a**, Amino acid sequence alignment of RopB from GAS with NrpR (SarA) from SAL. **b**, Amino acid sequence alignment of the leaderless intercellular peptide signal SIP from GAS with NIP (SarB) from SAL. The identical

residues between aligned molecules are highlighted in shaded boxes. **c**, Schematics of the mutant strains used to characterize NrpR-NIP signaling pathway in SAL. Organization of genes encoding *nrpR* and *nip* located upstream of *sarC-P* coding region is shown. The numbers below the line indicate the nucleotide positions relative to the translation start site (+1) of *sarC*. Since *nip* is encoded as part of the promoter for *sarC-P* operon, a *nip** mutation with the *nip* start codon (ATG) changed to stop codon (TAG) was constructed to disrupt *nip* translation but minimize the disruption *of sip*/*sarC-P* promoter (P_{sip}) (bottom panel). The nucleotide sequences underlined indicate the putative ribosomal binding site for NIP.

Fig S19

**Locus tag**	**Predicted function**	**Fold change in *nip** mutant**	**Fold change in *ΔnrpR mutant***	**Protein ID**
RSSL_00024	putative cytosolic protein	- 65.3015	- 70.24239	EJO15269.1
RSSL_00025	NRPS-PKS	- 66.32337	- 74.78175	EJO15270.1
RSSL_00026	NRPS-PKS	- 63.6474	- 66.95553	EJO15271.1
RSSL_00027	Dehydratase	- 55.13018	- 57.42981	EJO15272.1
RSSL_00028	Ketoreductase	- 56.32262	- 57.08425	EJO15273.1
RSSL_00029	PKS	- 43.61117	- 42.41811	EJO152741
RSSL_00030	Thioesterase	- 29.43982	- 27.61122	EJO15275.1
RSSL_00031	Malonyl-CoA-(acyl-carrier protein) transacylase	- 36.27568	- 33.15989	EJO15276.1
RSSL_00032	PPant transferase	- 29.88388	- 25.27362	EJO15277.1
RSSL_00033	Transposase	- 26.2434	- 26.95386	EJO152781
RSSL_00034	Transposase	- 25.36269	- 27.41807	EJO15279.1
RSSL_00035	Integrase/recombinase xerD	- 20.23268	- 18.24736	EJO15281.1
RSSL_00036	Hypothetical protein	- 18.24373	- 18.24736	EJO15281.1
RSSL_00037	Hypothetical protein	- 18.09474	- 16.05297	EJO15282.1

RSSL_00918	hypothetical protein	- 1.055825	- 9.106906	EJO15254.1
RSSL_01466	BioY protein	- 3.344894	- 3.373269	EJO16816.1
RSSL_02192	fructose-specific IIABC component	- 4.01602	- 1.308663	EJO16462.1
RSSL_02193	1-phosphofructokinase	- 3.077222	- 1.636629	EJO16461.1

Figure S19. **Genes downregulated in Δ*nrpR* or *nip** mutant strain compared to WT SAL**.

Fig S20

**Locus tag**	**Products**	**Fold change in *nip** mutant**	**Fold change in *ΔnrpR* mutant**	**Protein ID**
RSSL_00915	*Phage protein*	*1.75507*	*8.903858*	*EJO15251.1*

Figure S20. Genes upregulated in Δ*nrpR* or *nip mutant strain compared to WT SAL.**

Fig S21

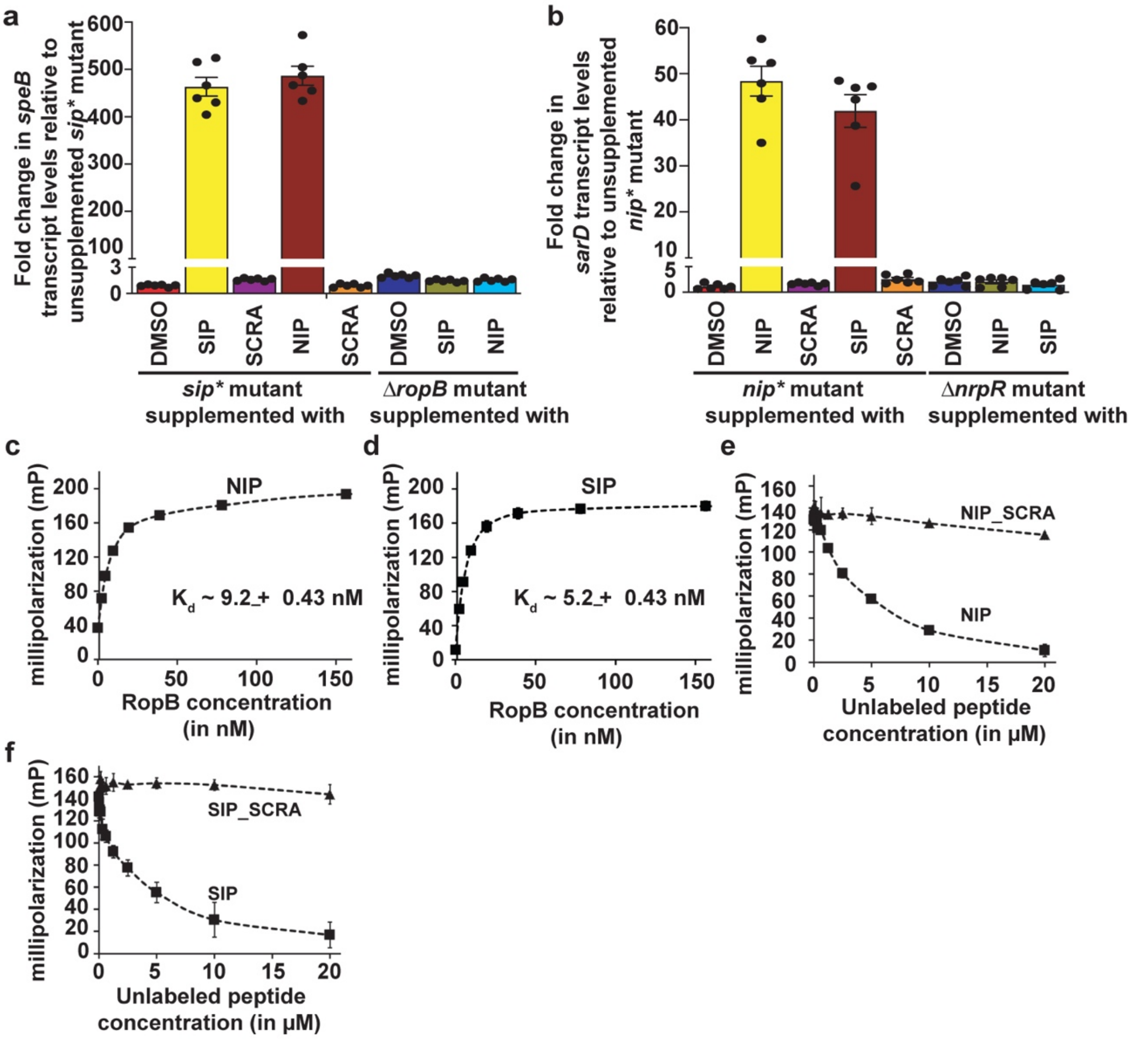

Figure S21. **Cross activation of heterologous SIP signaling pathway in GAS by SAL-NIP.** Supplementation of either GAS-*sip** (**a**) or SAL-*nip** (**b**) mutant with synthetic NIP or SIP, respectively, activate heterologous signaling pathways. **a**, The GAS-*sip** or GAS-Δr*opB* mutant was grown to late-exponential phase of growth (A_{600} ~ 1.5) and supplemented with 100 nM of the indicated synthetic peptides. **b**, The SAL-*nip** or Δ*nrpR* mutant was grown to mid-exponential phase of growth (A_{600} ~ 2.0) and supplemented with 100 nM of the indicated synthetic peptides. After 1 h incubation, cells were collected and transcript levels of *speB* or *sarD* were assessed by qRT-PCR. Cells supplemented with the carrier, DMSO, that was used to dissolve peptides were included as a control. Experiment was performed as three biological replicates and analyzed in duplicate. Data graphed were mean ± s.e.m. **c**, The affinity and direct interactions between NIP and RopB was assessed by fluorescence polarization (FP) assay and compared with RopB-SIP interactions (**d**). **e**, Ability of synthetic NIP or SCRA peptide to disrupt pre-formed RopB-SIP complex was assessed by FP assay

and compared to that of synthetic SIP (**f**). Binding assays were done in three independent occasions. Data graphed were mean ± s.e.m.

Fig S22

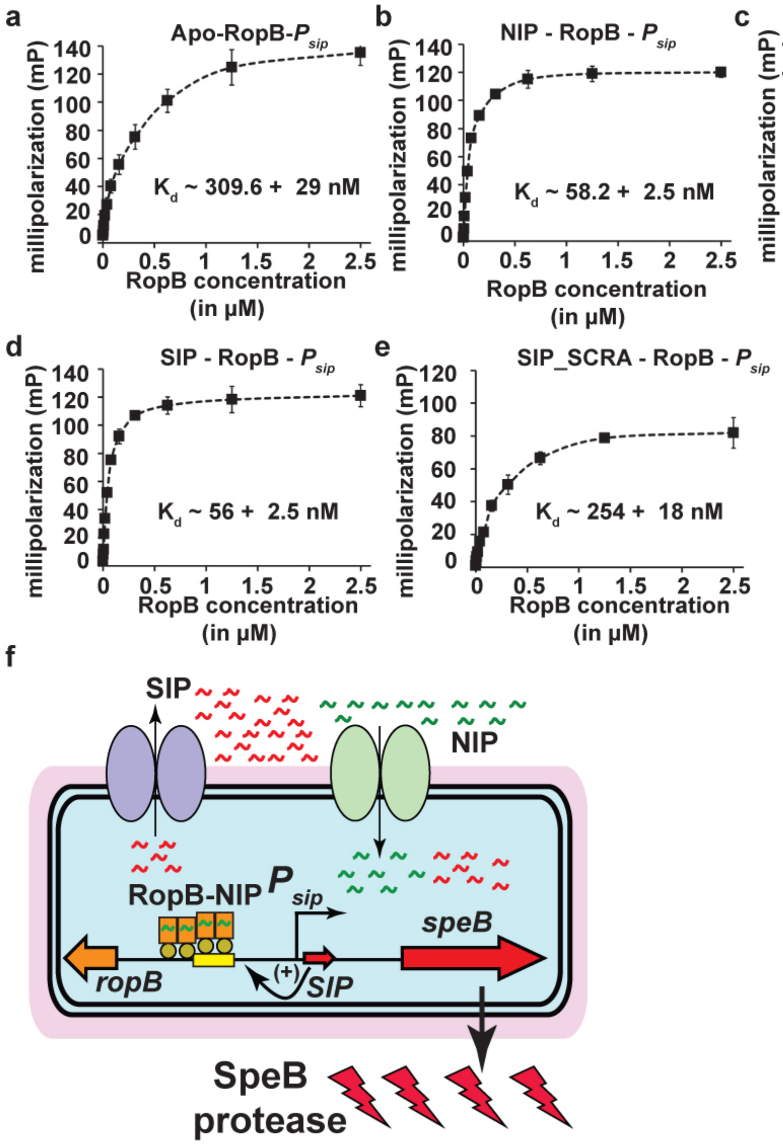

Figure S22. NIP activates *speB* expression by promoting high affinity interactions between RopB binding site in *sip* promoter (*P*$_{sip}$) and RopB. Analyses of interactions between FITC-labeled oligoduplex containing RopB-binding site and apo- (**a**) or NIP-bound (**b**) RopB by FP assay. The NIP-induced high affinity interactions between RopB and *P*$_{sip}$ were compared with scrambled NIP peptide (NIP-SCRA) (**c**), SIP (**d**), and SIP-SCRA (**e**). Binding assays were done in three independent occasions. Data graphed were mean ± s.e.m. **f**, The proposed model for *speB* upregulation by NIP via RopB-NIP interactions. NIP produced by SAL is hijacked by GAS, which causes initial induction of *sip* and *speB* expression by facilitating high affinity RopB- *P*$_{sip}$ interactions. Subsequently, upregulation of endogenous *sip* expression results in robust induction of *sip* and *speB* expression by a positive feedback mechanism.

Fig S23

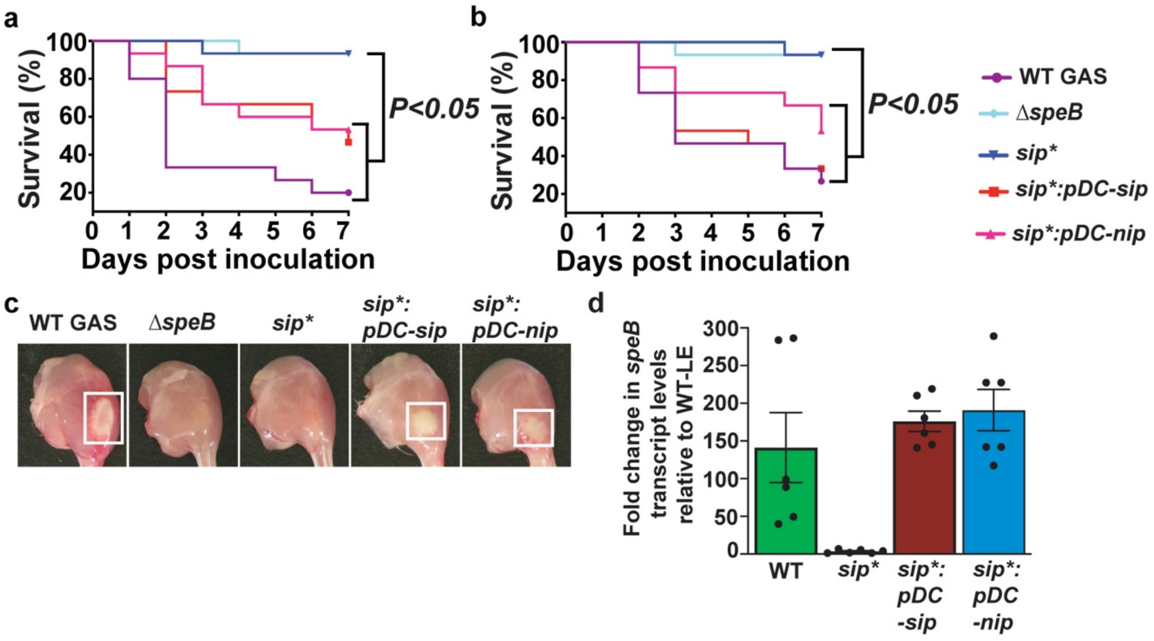

Figure S23. **NIP activates *speB* expression *in vivo* and contributes to GAS virulence in mouse models of infection. a**, Fifteen outbred CD-1 mice were inoculated intraperitoneally with each indicated strain. Kaplan-Meier survival curves with P values derived by the log-rank test are shown. The P value between the groups was < 0.0001. **b**, Fifteen outbred CD-1 mice per strain were injected intramuscularly with each indicated strain. Kaplan-Meier survival curves with P values derived by the log-rank test are shown. The P value between the groups was < 0.0001. **c**, Analyses of gross hindlimb lesions from mice infected with each indicated strain. Larger lesions with extensive tissue damage in mice infected with *speB*-expressing strains are boxed (white box). **d**, Analyses of *speB* expression in intramuscular lesions isolated from mice infected with the indicated strains. Samples were collected 48 h post infection from five mice per strain and analyzed in triplicate. Data graphed as mean ± s.e.m.

Fig S24

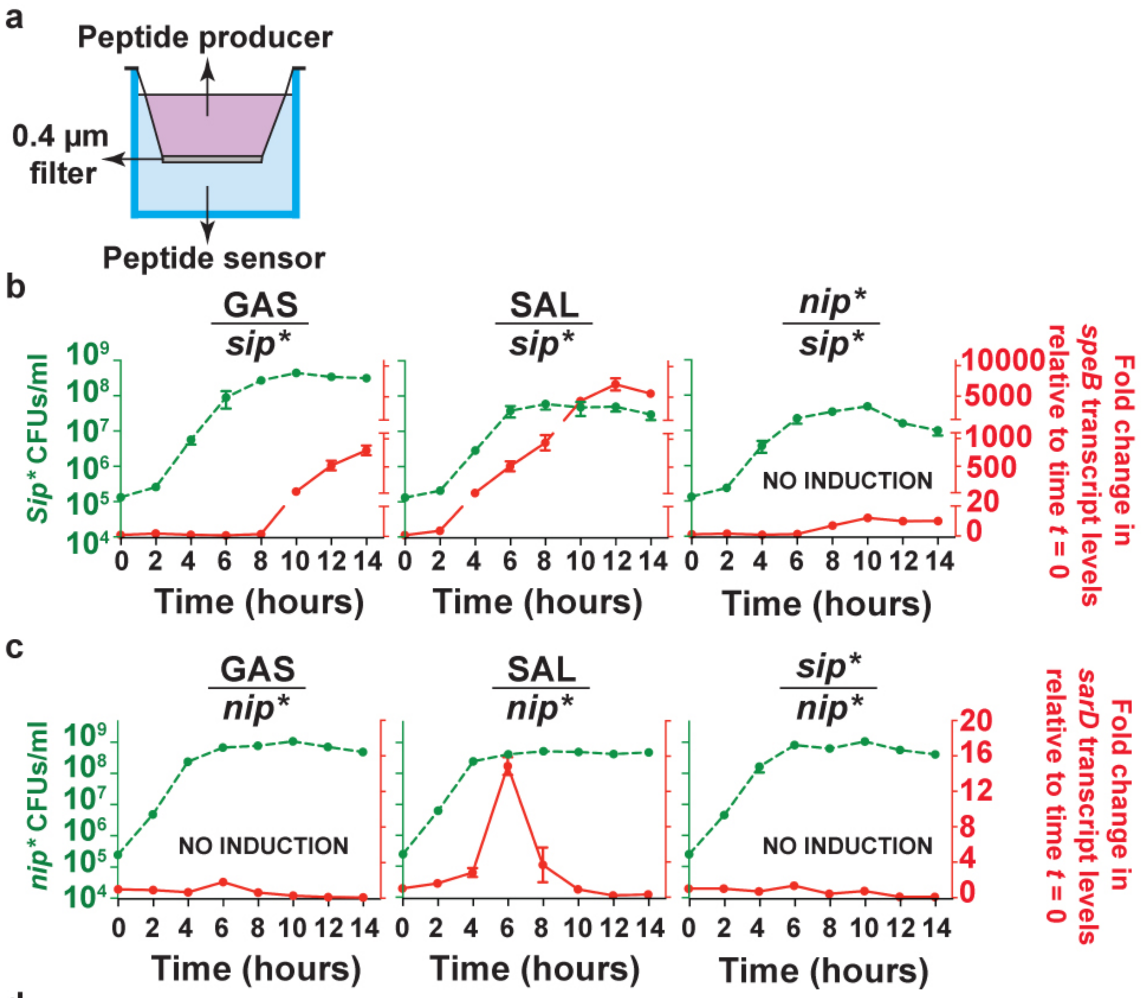

d

Co -inoculation with	Hours post inoculation at which onset of *speB* expression occurs	CFUs/ml of *sip** mutant at which onset of *speB* expression occurs	Fold change increase in *speB* expression at the onset in *sip** mutant	Maximum fold change in *speB* expression in *sip** mutant
WT GAS	10 h	4 x 10^8	60 X	795 X
WT SAL	4 h	3 x 10^6	45 X	7096 X

Figure S24. **GAS growth and *speB* transcript level kinetics during growth *in vitro* in the presence of GAS or SAL. a**, Schematic of trans-well is shown. **b**, The ability of WT GAS or SAL to activate *speB* expression in the *sip** mutant strain during co-cultivation was assessed. The *sip** mutant strain was inoculated at 10^5 CFUs/ml in the bottom well. One of the following strains (WT GAS or WT SAL or SAL *nip** mutant) was inoculated at 10^7 CFUs/ml in the top well. **c**, The ability of WT GAS or SAL to activate *sarD* expression in the *nip** mutant strain during co-cultivation was assessed. The *nip** mutant (10^5 CFUs/ml) strain was inoculated in the bottom well. One of the

following strains (WT GAS or WT SAL or GAS *sip** mutant) was inoculated in the top well at 10^7 CFUs/ml. The left y-axis represents the growth curve as determined by CFU analyses at the indicated time points. The right y-axis represents the fold change in *speB* or *sarD* transcript levels at the indicated time points, as measured by qRT-PCR. The fold change in transcript levels relative to the level in the starting culture (time point = 0 h) is shown. In. panels **b** and **c**, data from three biological replicates are shown and data graphed as mean ± s.e.m. **d**, Summary of data in panel B showing *speB* induction kinetics in the *sip** mutant strain grown in the presence of WT GAS or WT SAL.

Fig S25

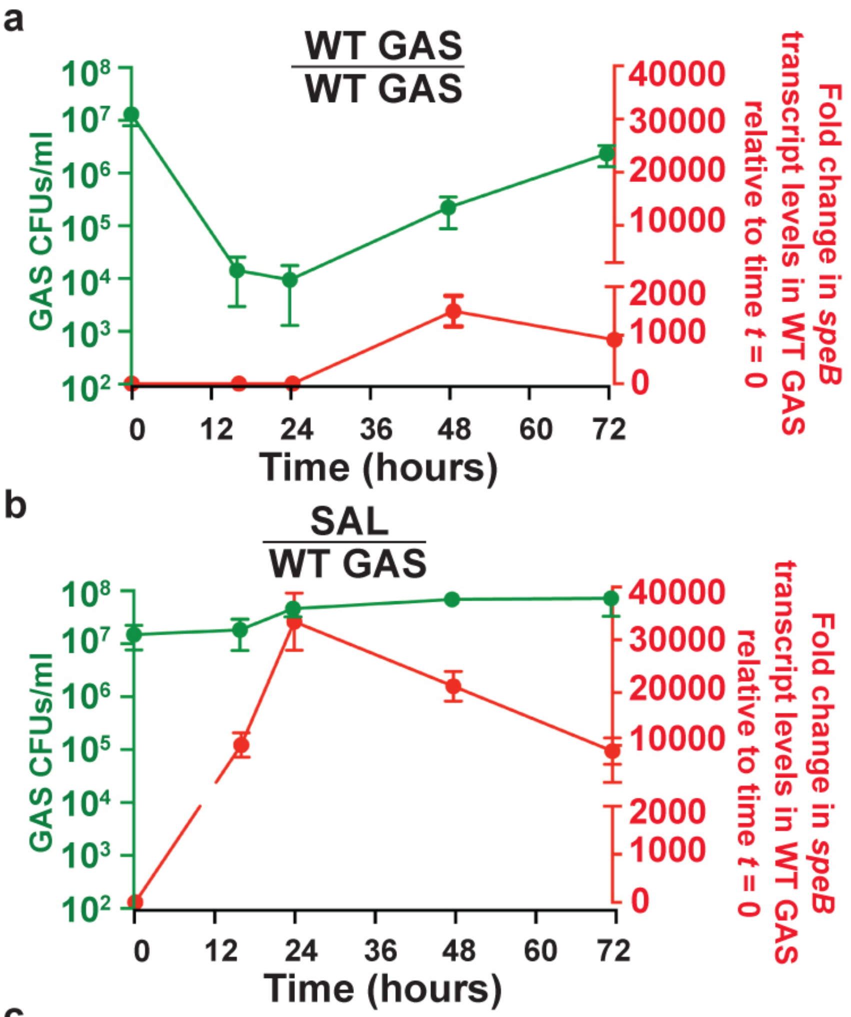

c

Co -inoculation with	Hours post inoculation at which onset of *speB* expression occurs	GAS CFUs/ml at which onset of *speB* expression occurs	Fold change increase in *speB* expression at the onset	Maximum fold change in *speB* expression
WT GAS	48 h	2.2×10^5	1500 X	1500 X
WT SAL	16 h	1.8×10^7	10000 X	33472 X

Figure S25. **GAS growth and *speB* transcript level kinetics during growth *ex vivo* in the presence of GAS or SAL in human saliva.** The ability of WT GAS (**a**) or SAL (**b**) to activate *speB* expression in WT GAS during co-cultivation in human saliva was assessed. Since *speB*-negative GAS strains are defective in survival in human saliva, we used WT GAS as the indicator strain in saliva studies. The WT GAS was inoculated at 10^7 CFUs/ml in the bottom well. WT GAS (**a**) or SAL (**b**) was inoculated in the top

well at 10^7 CFUs/ml. The left y-axis represents the growth curve as determined by CFU analyses at the indicated time points. The right y-axis represents the fold change in *speB* transcript levels at the indicated time points, as measured by qRT-PCR. The fold change in transcript levels relative to the level in the starting culture (time point = 0 h) is shown. Data from three biological replicates are shown. Data graphed as mean ± s.e.m. **c**, Summary of data in panel A and B showing *speB* induction kinetics in WT GAS grown in the presence of WT GAS or WT SAL.

Fig S26

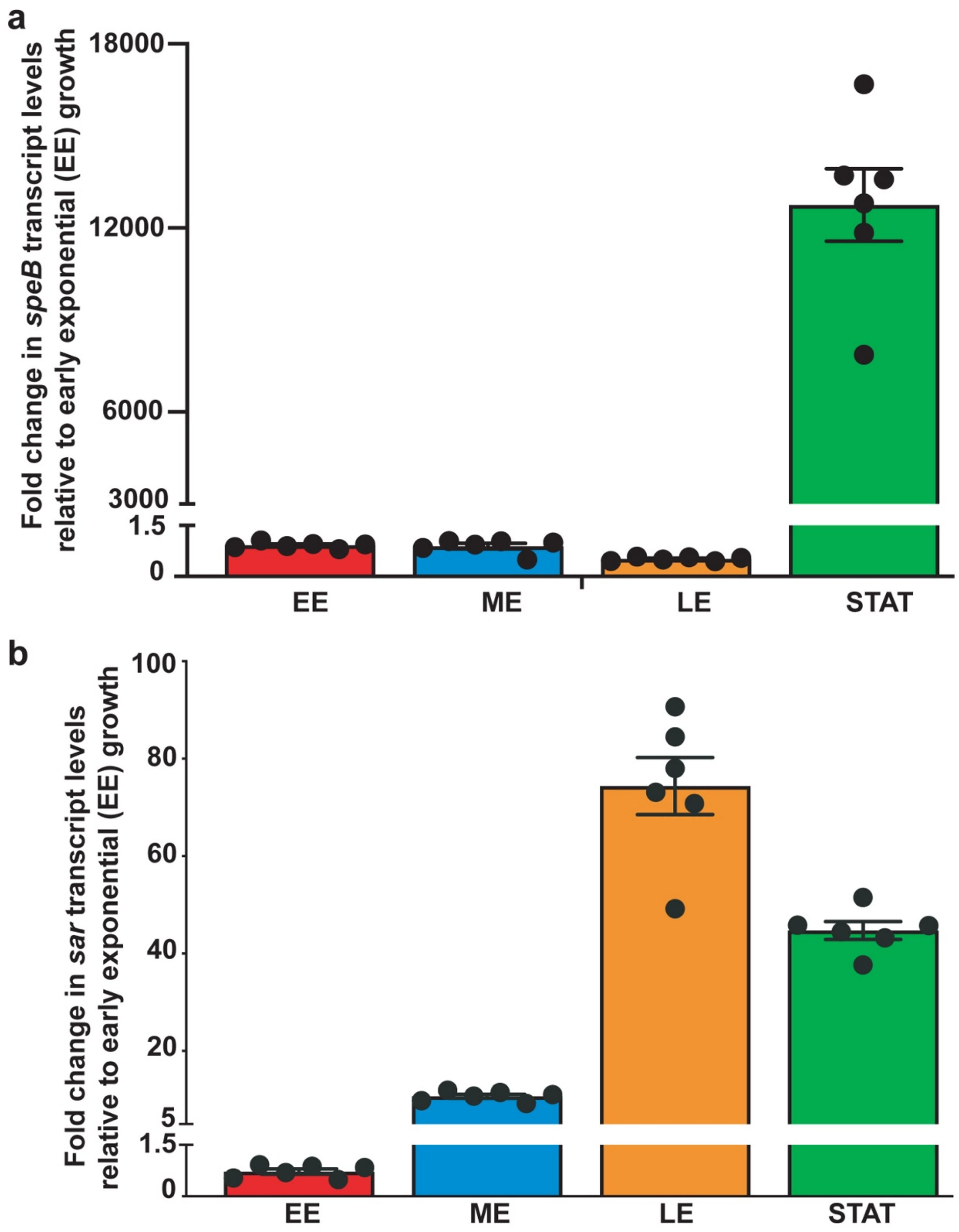

Figure S26. **Growth phase-dependent and -independent expression of *speB* (a) and *sarD* (b) in GAS and SAL, respectively.** Three biological replicates were grown on separate occasions and samples were collected at the indicated phases of bacterial growth. Transcript levels were assessed by qRT-PCR and analyzed in duplicate. Fold change in transcript levels relative to early exponential phase (EE) phase of growth is shown. EE – early exponential, ME – mid exponential, LE – late

exponential, and STAT – stationary phase of bacterial growth. Data graphed as mean ± s.e.m.

Fig S27

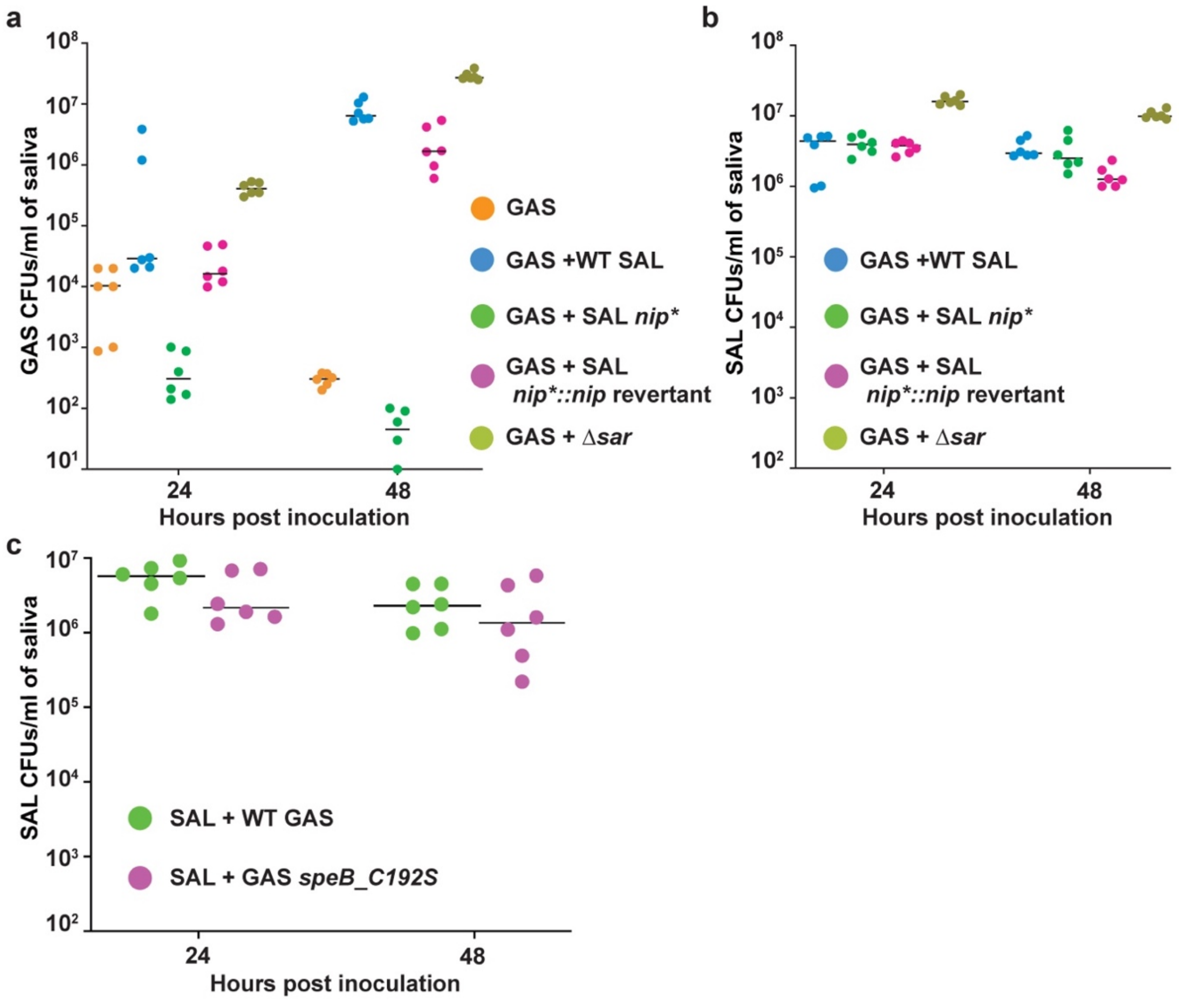

Figure S27. **GAS and SAL levels in saliva during co-cultivation with GAS**. **a,** The levels of indicated GAS strains in human saliva during co-cultivation with SAL *ex vivo*. **b,** The levels of indicated SAL strains in human saliva during dual species growth *ex vivo* with GAS. **c,** SAL levels in human saliva during dual species growth *ex vivo* with either WT GAS or GAS *speB_C192S* mutant strain. Bacterial burden was assessed by CFU analyses. Experiments were performed as biological triplicates and analyzed in duplicate. No statistical significance between groups were detected by Kruskal-Wallis analyses.

Fig S28

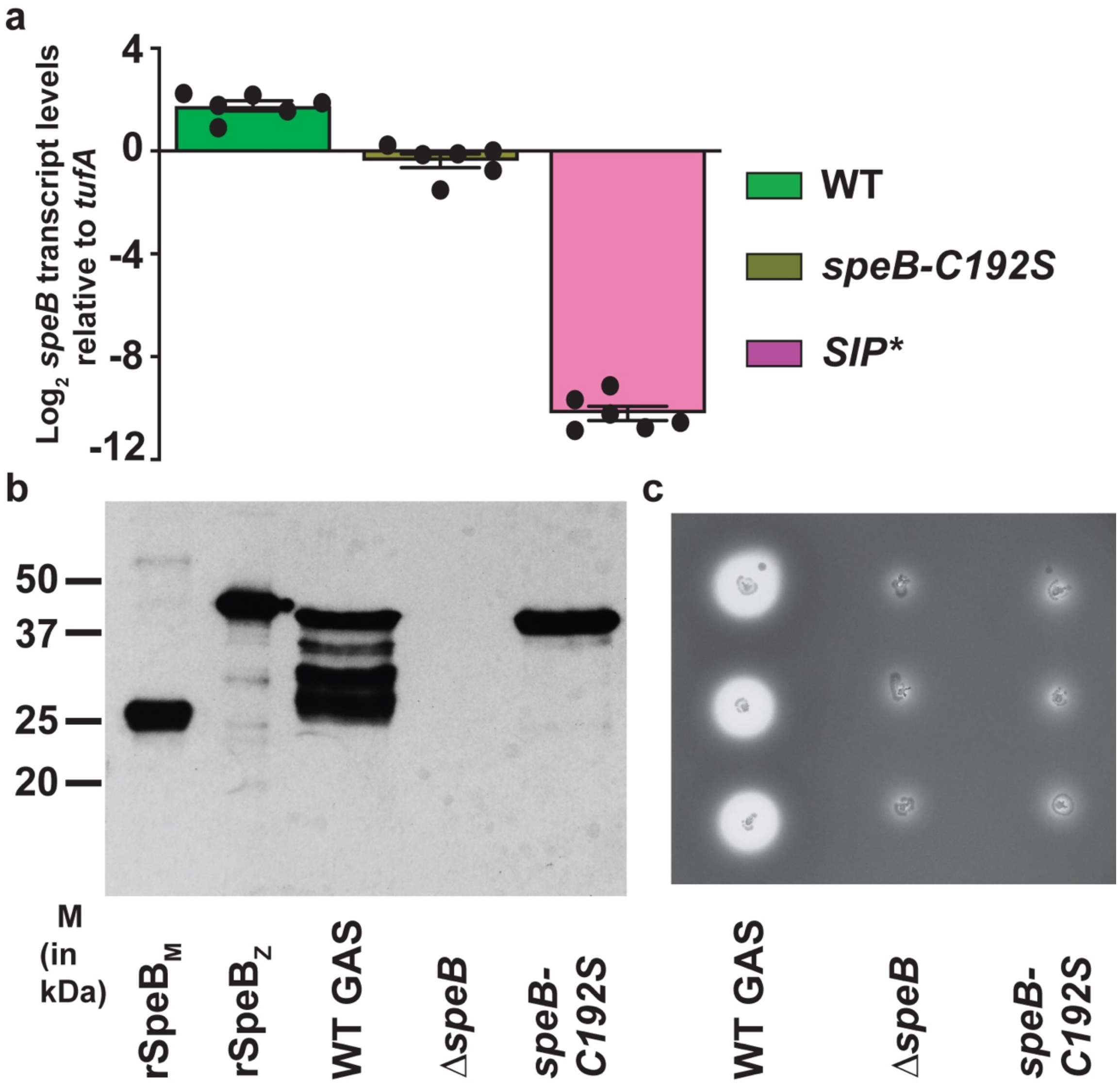

Figure S28. The expression of *speB* was unaffected in the GAS mutant strain encoding catalytically inactive *speB-C192S* mutant (**a**) but extracellular autocatalytic processing of SpeB zymogen to mature SpeB in the secretome (**b**), and corresponding SpeB protease activity (c) were affected. **a)** Cells were grown in laboratory medium to stationary phase of growth. Samples were collected and *speB* transcript levels were assessed by qRT-PCR. The log-fold changes in *speB* transcript levels relative to house-keeping gene *tufA* are shown. Experiments were performed as biological triplicates and analyzed in duplicate and data were presented as mean ± s.e.m. **b**, Immunoblot analyses of cell-free culture supernatant obtained from the indicated strains for the presence of secreted SpeB. rSpeB$_M$ – purified recombinant mature SpeB; rSpeB$_Z$ – purified recombinant zymogen form of SpeB. Multiple bands in the

culture supernatant from WT GAS indicate the intermediate forms of SpeB arising from autocatalytic maturation process from zymogen to mature form. The catalytically inactive *speB-C192S* mutant does not undergo autocatalytic maturation steps, which is supported by the presence of only the zymogen form of SpeB in the culture supernatant from the *speB-C192S* mutant strain. Three independent experiments were performed and representative blot is shown. **c**, Milk plate clearing assay to demonstrate lack of SpeB protease activity in *speB-C192S* mutant strain. Protease activity was determined by the presence of a clear zone around the bacterial growth.

Fig S29

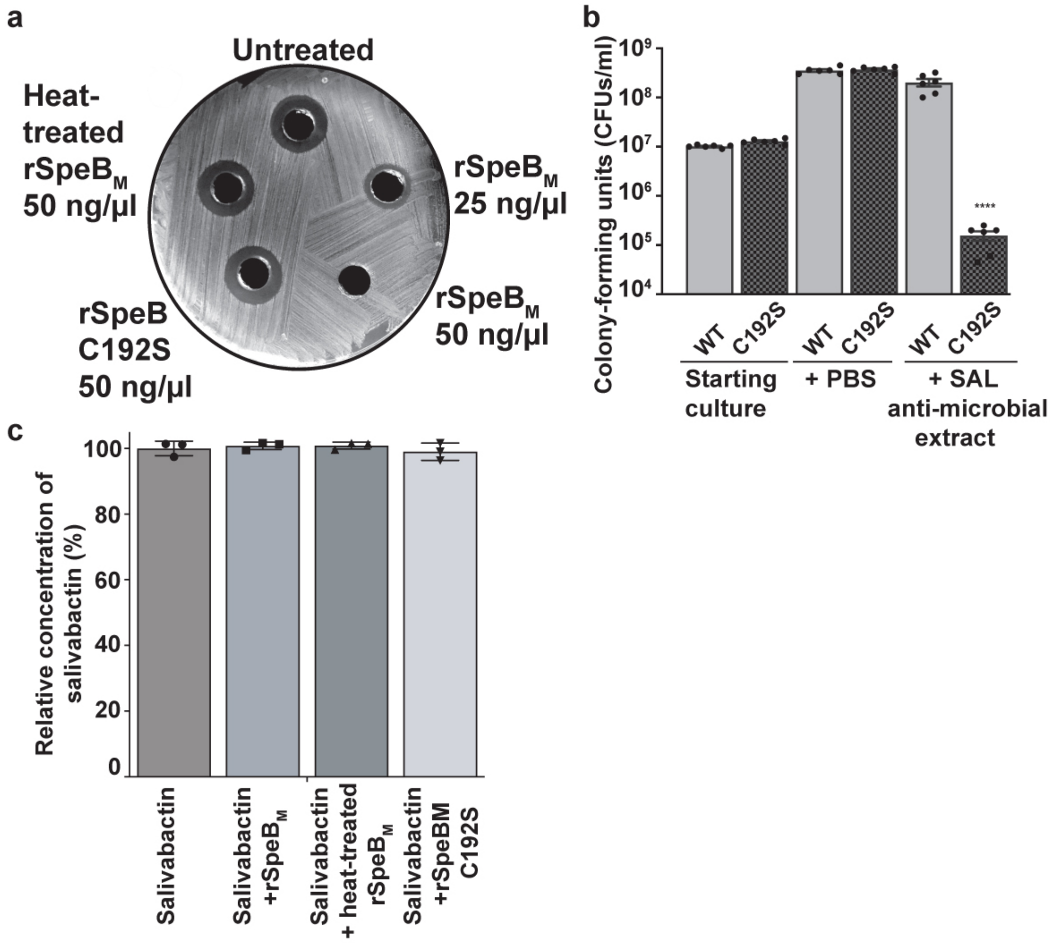

F

igure S29. **SpeB degrades SAL lantibiotics and aids GAS survival but unable to degrade salivabactin. a,** Incubation of SAL lantibiotics with enzymatically active SpeB protease resulted in loss of anti-GAS activity. SAL was grown on agar plates overnight at 37˚C and SAL growth was scraped off the plate surface. The SAL growth was frozen and thawed at room temperature. The supernatant containing lantibiotic extracts was separated by centrifugation at 3000 rpm for 10 minutes and used to test the antimicrobial activity. The clarified supernatant was incubated with indicated concentrations of purified recombinant WT SpeB$_M$ or C192S$_M$ at 37˚C for 2 h. GAS lawn was swabbed on an agar plate and SpeB-treated or untreated SAL lantibiotic extracts were placed in wells in the agar plate. Heat-treated rSpeB$_M$ was prepared by heating rSpeB$_M$ at 90˚C for 5 minutes. The presence of zone of clearance around the wells indicates the presence of antimicrobial activity. **b,** The enzymatically inactive *speB*-C192S mutant strain is more sensitive to SAL lantibiotic extracts compared to WT GAS. The WT GAS or *speB_C192S* mutant strain was either mock treated with

PBS or treated with SAL lantibiotic extracts for 2 h at 37˚C. Cells were collected, serially diluted and plated. The sensitivity to lantibiotic extracts was assessed by enumerating colony-forming units. The experiment was performed in triplicates and analyzed in duplicate. Data graphed were mean ± s.e.m. *P* value (**** - $P < 0.0001$) was determined by two-tailed Mann Whitney test. **c**, Salivabactin is insensitive to the protease activity of recombinant WT SpeB. Salivabactin was incubated with $rSpeB_M$, heat-treated $rSpeB_M$, or enzymatically inactive $rC192S_M$**.** The relative abundance of salivabactin from different treatments were compared, based on the extracted ion chromatograms. Data are shown as mean ± s.d. ($n = 3$).

Fig S30

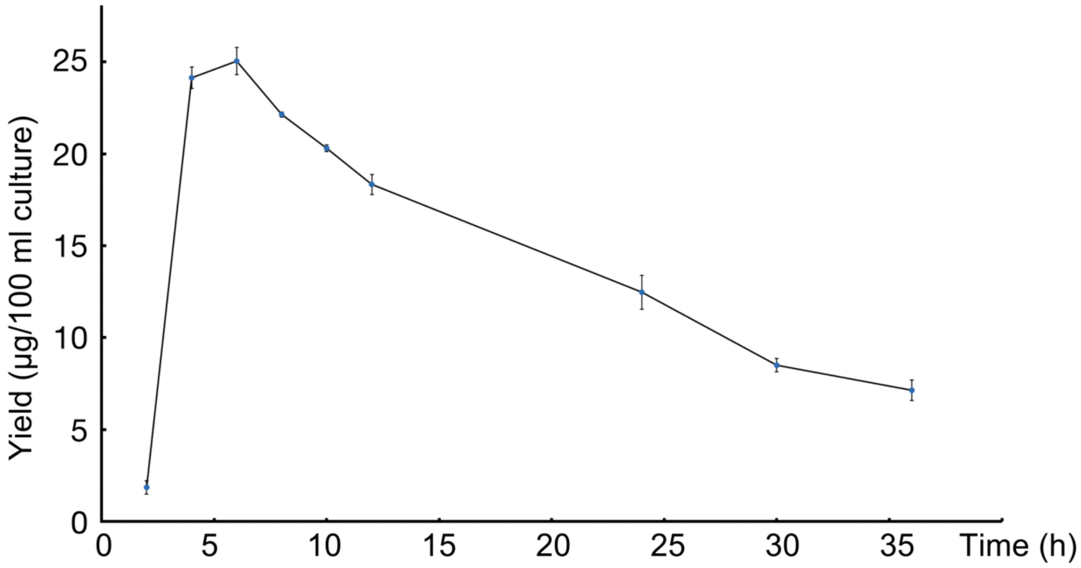

Figure S30. **Time course for the production of salivabactin by SAL during growth *in vitro*.** The WT SAL was grown in THY broth (Todd-Hewitt broth + 3 g/L yeast extract) at 37 °C and the cultures were extracted by organic solvent and analyzed by HPLC MS. Experiments were performed as biological triplicates and data are shown as mean ± s.d.

Fig S31

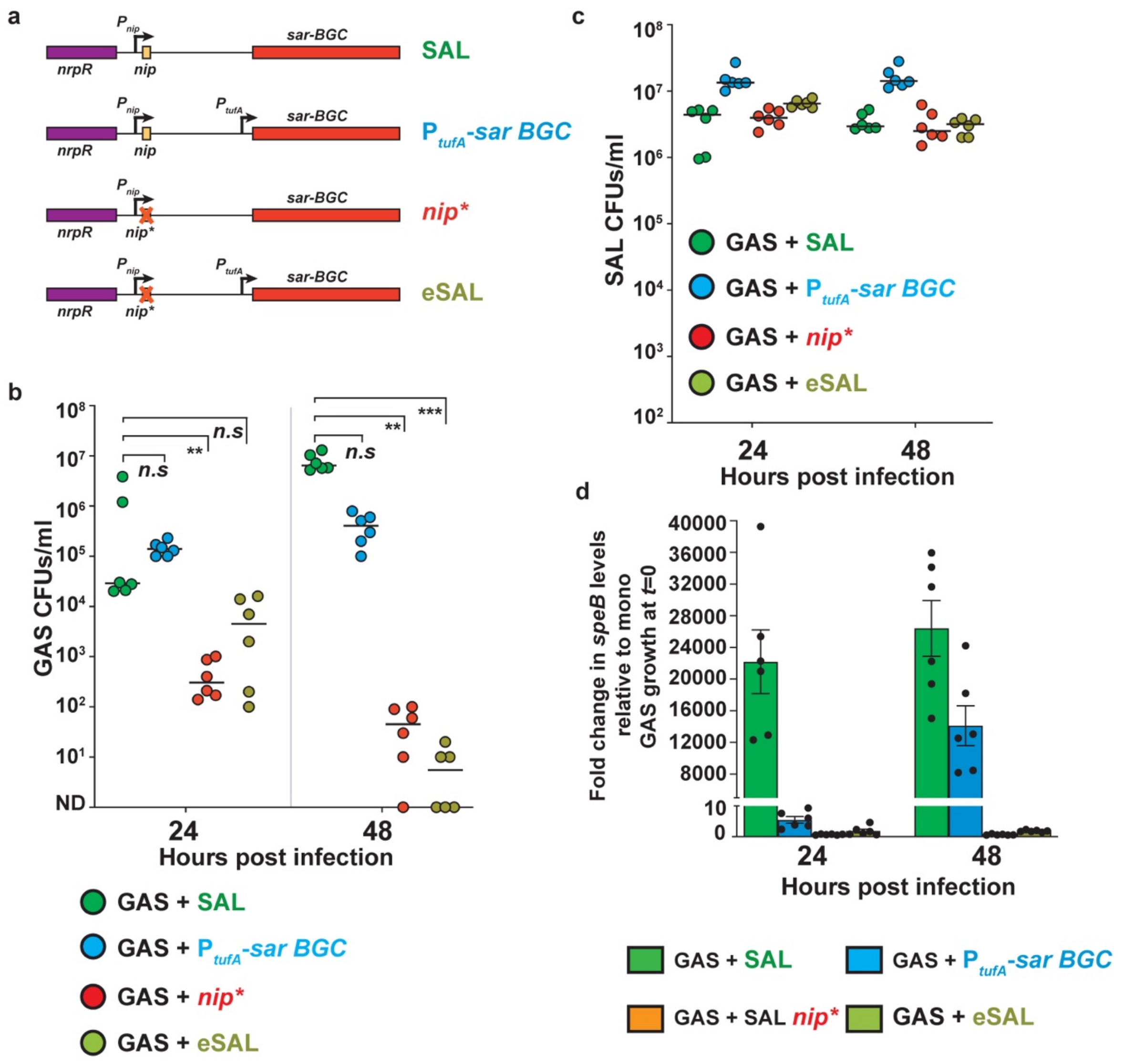

Figure S31. The *nip mutation has more pronounced role in GAS growth inhibition than constitutive expression of *sar-BGC*.** Characterization of the two single modifications in eSAL **(a)** for GAS growth promotion (**b**), SAL survival (**c**), and *speB* expression (**d**) in human saliva *ex vivo*. Experiments were performed as biological triplicates and analyzed in duplicate. In panels **b** and **d,** GAS + WT SAL group as reference, and statistical significance was analyzed by multiple comparison Kruskal-Wallis test. At 24 hpi, **, $P = 0.008$, whereas at 48 hpi, **, $P = 0.002$, and ***, $P = 0.0002$, *n.s*, not significant. In panel **d**, data were presented as mean ± s.e.m. In panel **c**, a multiple comparison Kruskal-Wallis test was performed to determine statistically significant differences compared to GAS infected with WT SAL as reference group. No statistically significant differences in GAS CFUs between individual groups and reference group were observed.

Fig S32

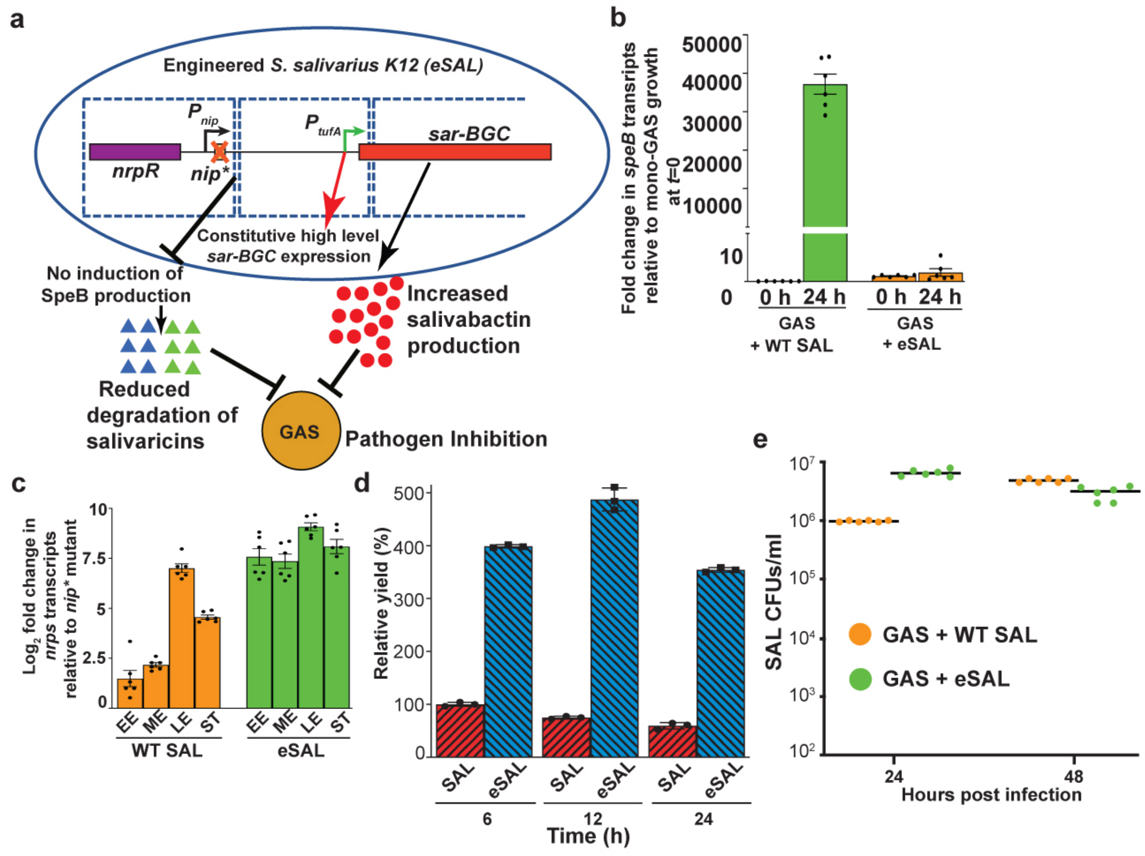

Figure S32. **a,** Schematics of genetic modifications in engineered *S. salivarius* (eSAL). The *nip** mutation was introduced to abolish NIP production in *S. salivarius* and early induction of SpeB production in GAS during dual species growth. Transcription of *sar* operon was coupled with constitutionally active P_{tufA} promoter that drives high level growth phase-independent *sar-BGC* expression. Delayed SpeB protease production is likely to disarm pathogen defense and result in increased salivaricin levels due to reduced degradation by SpeB. Collectively, increased salivaricin and salivabactin levels may lead to improved pathogen inhibition and clearance of GAS in the host. **b,** The eSAL failed to induce *speB* expression during dual species growth in human saliva. Transcript levels of *speB* in GAS grown in human saliva in the presence of SAL or eSAL were compared. Fold change in *speB* transcript levels relative to mono species GAS growth at *t*=0 h is shown. **c,** Comparison of *sarD* expression profile between WT *S. salivarius* and eSAL during different phases of bacterial growth in laboratory medium. EE – early exponential phase (A_{600} ~0.5); ME – mid-exponential phase (A_{600} ~1.0); LE -late-exponential phase (A_{600} ~2.0); and ST – stationary phase of growth (A_{600} ~3.0). In panels **b** and **c**, data were presented as mean ± s.e.m. **d,** Production of salivabactin by WT SAL (red) and eSAL (blue). The WT SAL and eSAL were grown in THY broth (Todd-Hewitt broth + 3 g/L yeast extract) at 37°C. Three

independent cultures collected at indicated time points were extracted by organic solvent and analyzed by HPLC–MS. Data are shown as mean ± s.d. ($n = 3$). **e,** The levels of SAL or eSAL during growth in the presence GAS in saliva, as assessed by CFU analyses. In panels **b, c**, and **e**, data were derived from biological triplicates.

Figure S33

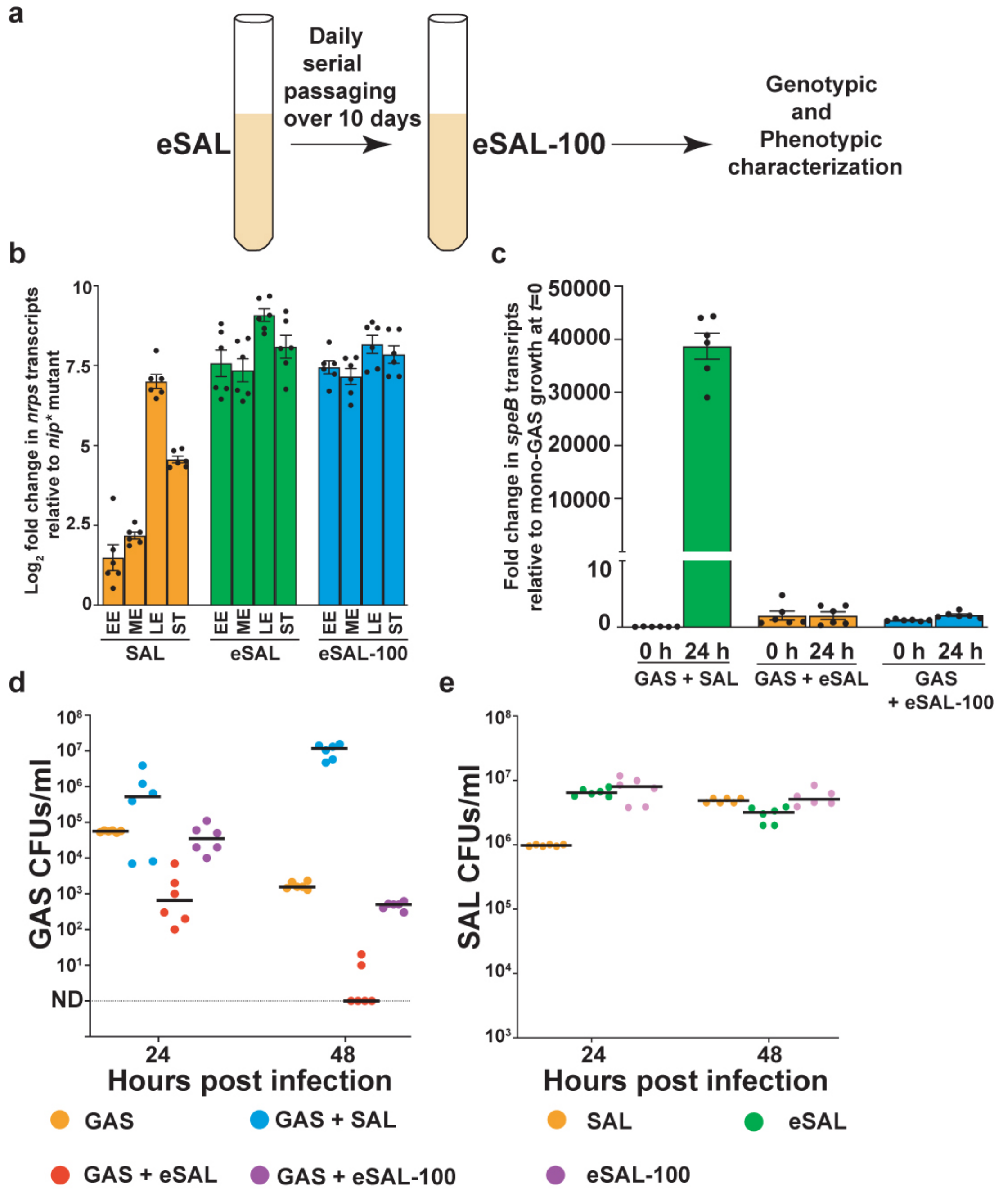

Figure S33. Genetic modifications introduced in engineered SAL (eSAL) are stable and functional phenotypes associated with eSAL remain unaltered. **a** eSAL was serially passaged daily for 10 days and the 100th generation (eSAL-100) was characterized for **b)** constitutive expression of *sar-BGC*, **c)** cross activation of *speB* expression in GAS and GAS growth promotion (**d**) during dual species growth in human saliva *ex vivo*, and **e)** SAL survival in human saliva. In panels **b, c**, **d**, and **e**, data were derived from biological triplicates. In panels **b** and **c**, data were presented as mean ± s.e.m.

Figure S34

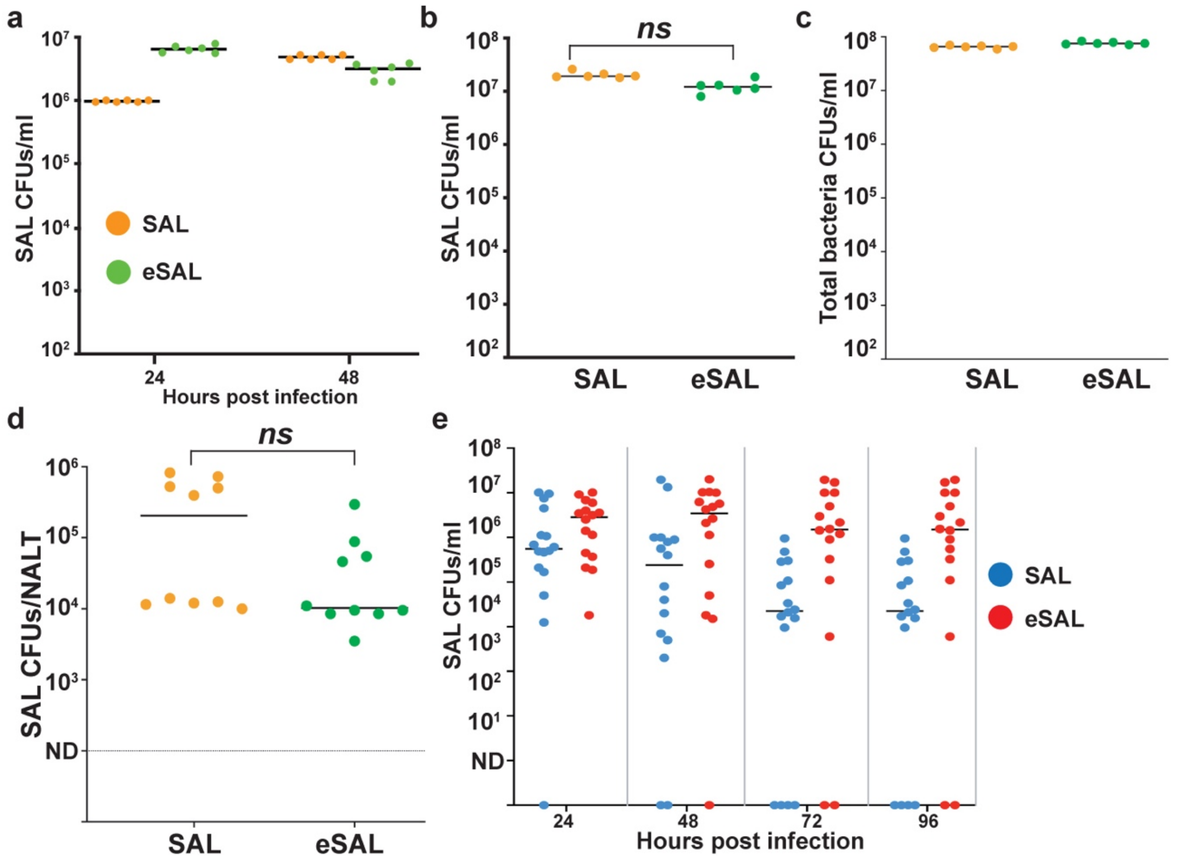

Figure S34. The engineered modifications did not affect the fitness of eSAL. SAL or eSAL was inoculated at 10^7 CFUs/ml in sterile (**a**) or non-sterile (**b**) human saliva with intact salivary microbiome and SAL levels were assessed by enumerating CFUs in samples collected at the indicated time points. Total bacterial count in non-sterile saliva (**c**) is shown. Three independent growth collected at indicated time points were used. Mice (*n* = 10 per group) were injected with 10^8 CFUs/ml of SAL or eSAL either intranasally (**d**) or intravaginally (**e**). The NALTs were collected at 24 hpi from mice infected intranasally, whereas the vaginal lumen was swabbed at the indicated time points in mice infected intravaginally. SAL burden was assessed by CFU analyses. Data were presented as mean ± s.e.m. In all panels, a two-tailed Mann Whitney test was performed to determine statistically significant differences between samples. No statistically significant differences in CFUs between groups were observed.

Figure S35

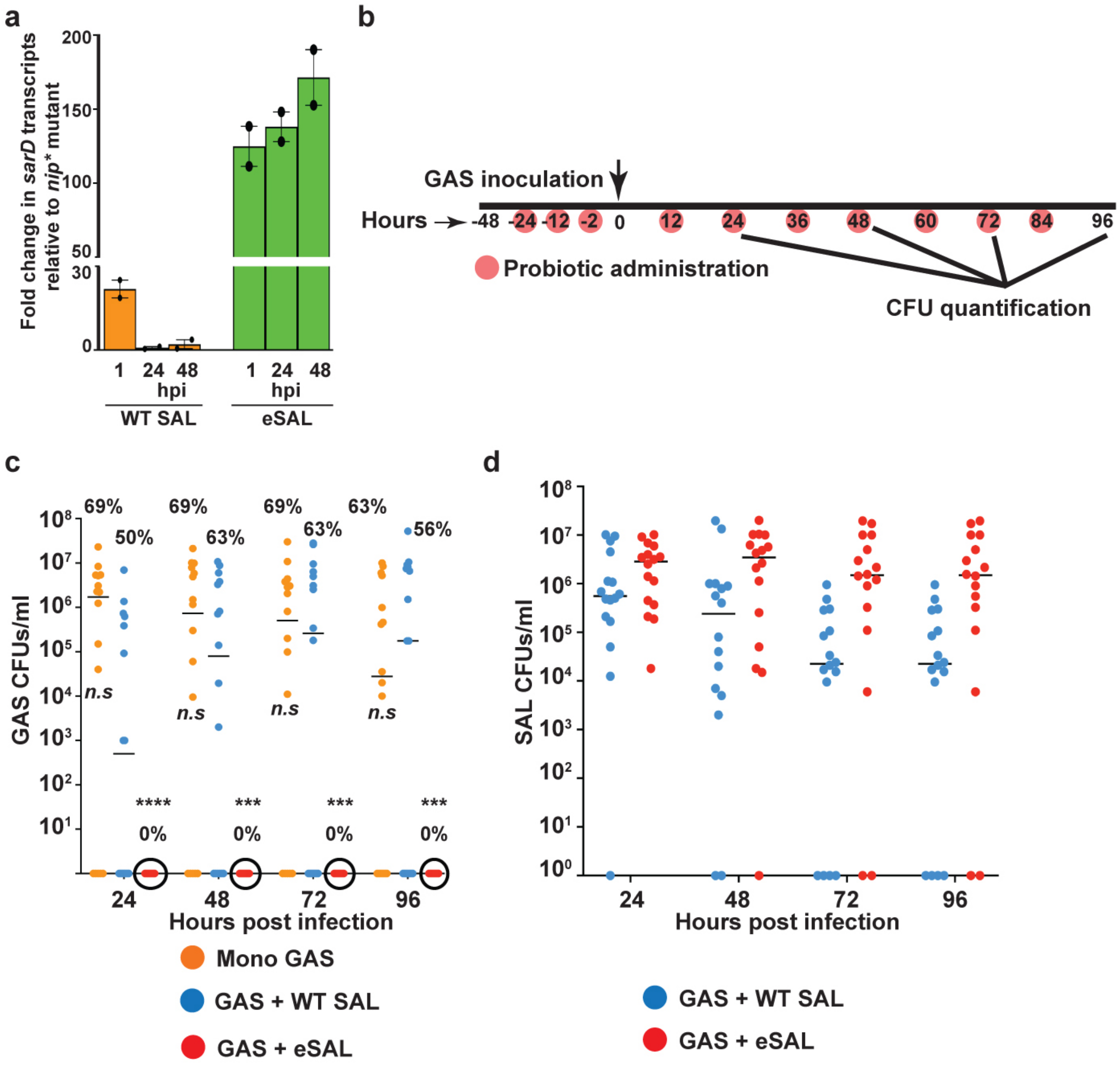

Figure S35. The eSAL is effective in preventing long term vaginal mucosal GAS colonization. **a,** Mice (*n*=10 mice/group) were given either WT SAL or eSAL intravaginally and *sarD* levels were assessed at the indicated time points by qRT-PCR. Pooled samples from each group (*n*=10 per group) were used for RNA extraction and transcript level analyses. Samples were analyzed in duplicate and data were presented as mean ± s.e.m. **b**, Experimental design to assess the probiotic efficacy of eSAL. Mice (*n*=16 mice/group) were given either WT SAL or eSAL at 12 h intervals. eSAL was more efficacious than WT SAL in preventing GAS colonization in mouse vaginal lumen. Each group (*n*=16 mice/group) received 10^8 CFUs of either WT SAL or eSAL intravaginally at the indicated time points. One day after the first dose of probiotic administration, single dose (10^3 CFUs) of GAS was given intravaginally. Swabs were collected at the indicated time points. GAS (**c**) and SAL (**d**) burden was

assessed by CFU analyses. The circles indicate the lack of detectable GAS colonies in eSAL-treated group. The numbers on the top indicate the percentage of animals colonized by GAS in each group. In panel **c**, statistical significance was analyzed by two-tailed Mann-Whitney test. **, $P<0.01$, ****, $P<0.0001$, *n.s,* not significant.

Figure S36

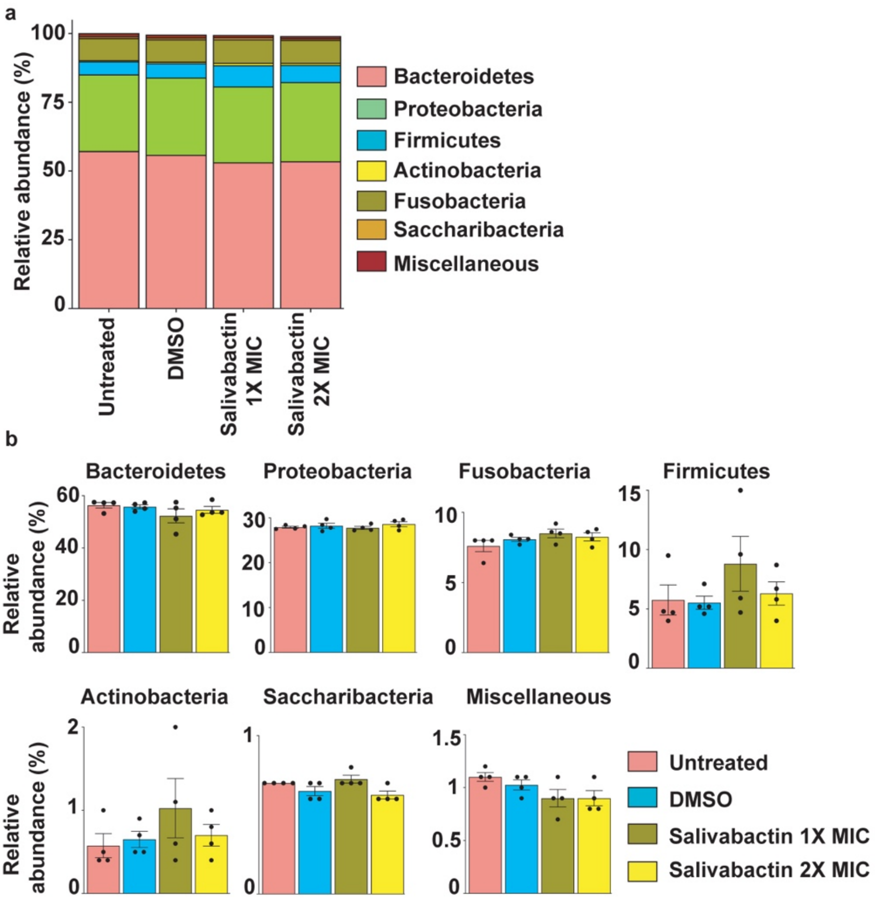

Figure S36. Salivabactin treatment did not cause dysbiosis of salivary microbiome. a, Saliva was collected from 4 healthy donors. Each sample was treated with one of the following for 16 h: DMSO, purified salivabactin at 1X MIC, and salivabactin at 2X MIC. Relative abundance of bacteria taxa at phylum level is shown. **b**, Relative abundance of individual phylum among the samples is shown. Samples collected from 4 individuals were treated as biological replicates and each replicate was analyzed in triplicate. Data were presented as mean ± s.e.m. No statistical significance was found among the samples. A multiple comparison Kruskal-Wallis test was performed to determine statistically significant differences compared to untreated group.

Figure S37

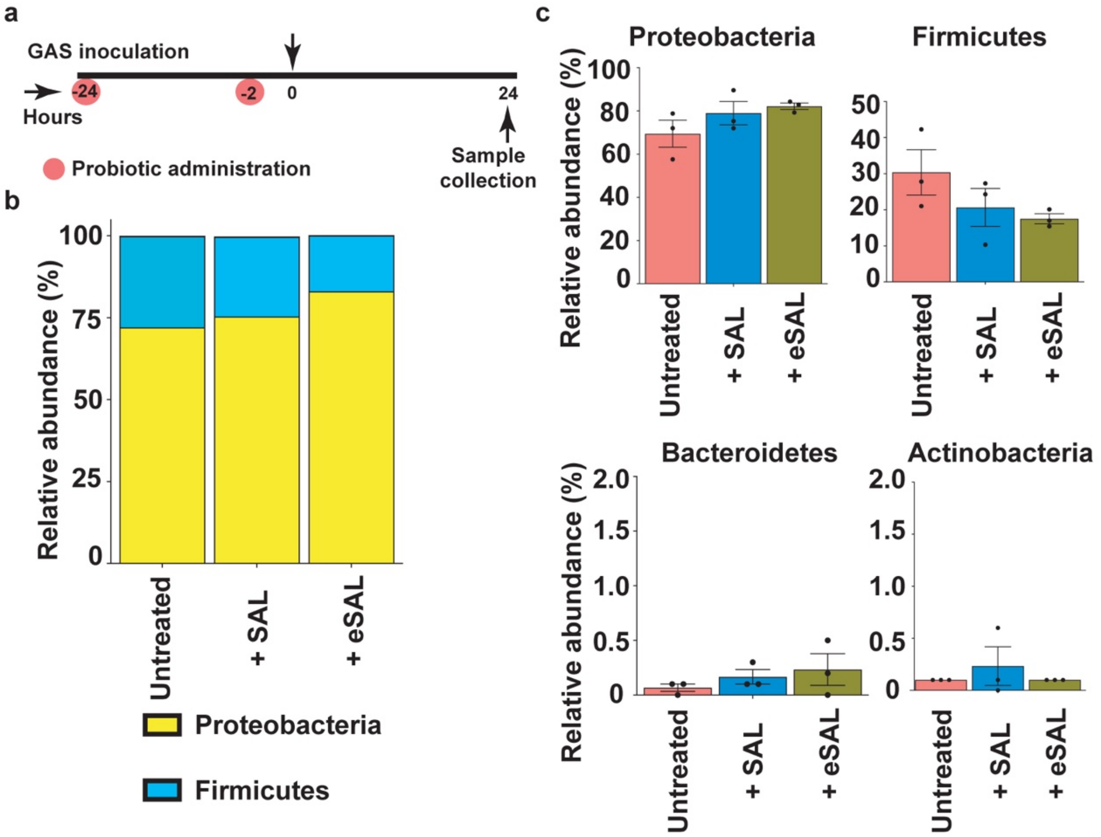

Figure S37. Probiotic treatment with SAL or eSAL did not cause dysbiosis of murine nasopharyngeal microbiome. a, Experimental design for the administration of SAL or eSAL. Mice (*n*=3 mice/group) were given 10^8 CFUs of either SAL or eSAL intranasally at the indicated time points. The nasopharyngeal swabs were collected at the indicated time points and processed for microbiota composition by 16S rRNA analyses. **b**, Relative abundance of bacteria taxa at phylum level is shown. **c**, Relative abundance of individual phylum among the samples is shown. Data were presented as mean ± s.e.m. A multiple comparison Kruskal-Wallis test was performed to determine statistically significant differences compared to untreated group. No statistically significant differences were found among the samples.

Figure S38

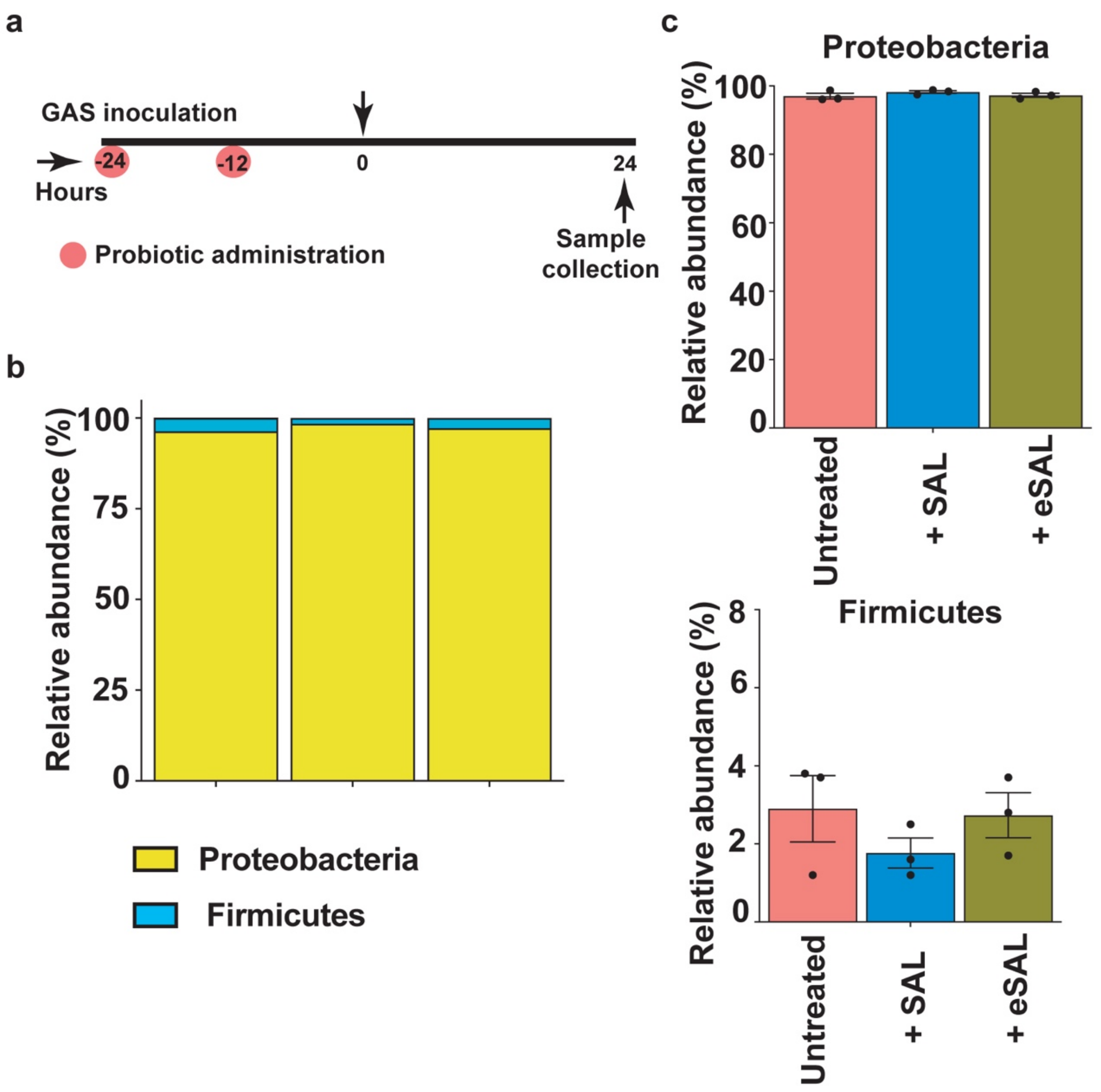

Figure S38. Probiotic treatment with SAL or eSAL did not cause dysbiosis of murine vaginal microbiome. a, Experimental design for the administration of SAL or eSAL. Mice (*n*=3 mice/group) were given 10^8 CFUs of either SAL or eSAL intravaginally at the indicated time points. The vaginal swabs were collected at the indicated time points and processed for microbiota composition by 16S rRNA analyses. **b**, Relative abundance of bacteria taxa at phylum level is shown. **c**, Relative abundance of individual phylum among the samples is shown. Data were presented as mean ± s.e.m. A multiple comparison Kruskal-Wallis test was performed to determine statistically significant differences compared to untreated group. No statistically significant differences were found among the samples.

Table S1. Bacterial strains and plasmids used in this study

Strain or Plasmid	Description	Reference
**Strains**		
WT GAS	Invasive isolate MGAS10870, serotype M3	3
MGAS2221	Invasive isolate, serotype M1	4
MGAS15249	Invasive isolate, serotype M59	5
MGAS26844	Pharyngeal isolate, M89	6
MGAS9429	Pharyngeal isolate, M12	7
Δ*ropB*	MGAS10870Δ*ropB::aad9*	8
Δ*speB*	MGAS10870Δ*speB::aad9*	8
*Sip**	Mutant strain that has the start codon of *sip* changed to stop codon in MGAS10870	9
speB_C192S	Mutant strain that has the catalytic residue Cys192 replaced with catalytically inactive Cys192Ser in MGAS10870	This study
WT SAL	*Streptococcus salivarius* K12 ATCC strain number BAA-1024	10

SAL marker	WT SAL containing erythromycin resistance marker at locus RSSL_00112 encoding a hypothetical protein of unknown function	This study
*nip**	Mutant strain that has the start codon of *nip* (*sarB*) changed to stop codon in WT SAL	This study
nip::nip*	Revertant strain in which the stop codon introduced in the *nip** mutant strain was reversed to start codon	This study
Δ*nrpR*	Mutant strain that has the *nrpR* (*sarA*) coding region inactivated by marker less deletion in WT SAL	This study
Δ*nrpR::nrpR*	Revertant strain in which the *nrpR* coding region was reintroduced in the Δ*nrpR* mutant strain	This study
Δ*salAB*	Mutant strain that has *salA* and *salB* genes inactivated by marker less deletion	This study
Δ*sar*	Mutant strain that has the entire *sarC-P* BGC inactivated by replacement with chloramphenicol resistance marker	This study
Δ*salAB*/Δ*sar*	Mutant strain that has the deletion of *salA, salB*, and *sarC-P* genes	This study
E. coli DH5α	Host strain for cloning purposes	
E. coli BL21(DE3)	Host strain for protein overexpression, *F-, ompT, hsdSB(rB-mB-), gal (λ c I 857, ind1, Sam7, nin5,*	

	lacUV-T7 gene1), dcm(DE3)	
**Plasmids**		
pJL	Low-copy number plasmid capable of replication in *Escherichia coli*, but a suicide vector in GAS. Chloramphenicol resistant. Used to generate isoallelic GAS or SAL mutants	[11]
pET-28a	Overexpression vector for N-terminally hexahistidine tagged recombinant proteins, Km^R	Novagen

Table S2. Primers used in this study

Primer	Sequence 5' – 3'	Purpose
pJL_marker A	GTATCGATAAGCTTGATATCGAATTCCTGCAGCCCGGGGGATCTGCTTAGACTGATCTTAGCTTTTCC	5' primer for 5' region of RSSL_00112 to replace RSSL_00112 with erythromycin resistance marker
pJL_marker B	CATAACTTCTTTTACGTTTCCGCCCGTAAATTTAATCACTTTATTAG	3' primer for 5' region of RSSL_00112 with erm sequence overlap
pJL_marker C	CTAATAAAGTGATTAAATTTACGGGCGGAAACGTAAAAGAAGTTATG	5' primer of erm sequence with RSSL_00112 overlap
pJL_marker D	GCCGAACCAGTTCTCACTATCTTCCCTTTAGTAACGTGTAAC	3' primer of erm sequence with RSSL_00112 overlap
pJL_marker E	GTTACACGTTACTAAAGGGAAGATAGTGAGAACTGGTTCGGC	5' primer for 3' region of RSSL_00112 to replace RSSL_00112 with erm sequence overlap
pJL_marker F	CTCACGTTAAGGGATTTTGGTCATGAGATTATCAAAAAGGATCAGTCCCTCACATATCATAGTATGGCATTGT	3' primer for 3' region of RSSL_00112 to replace RSSL_00112

	C	with erythromycin
pJL_Δ*nrpR* A	GTATCGATAAGCTTGATATCGAATTCCTGCAGCCCGGGGGATCTCCCATAGCTTTATTAAACGAAAATTTCTTTAC	5' primer for 5' region of *nrpR* to delete *nrpR*
pJL_Δ*nrpR* B	GGATGAAATTAATGCATAAATGAAGATGATCGATTGCTTGTCC	3' primer for 5' region of *nrpR* to delete *nrpR*
pJL_Δ*nrpR* C	GGACAAGCAATCGATCATCTTCATTTATGCATTAATTTCATCC	5' primer for 3' region of *nrpR* to delete *nrpR*
pJL_Δ*nrpR* D	GGGATTTTGGTCATGAGATTATCAAAAAGGATCGATATGTCAGGAGCCTATATGCCACTAG	3' primer for 3' region of *nrpR* to delete *nrpR*
pJL *nip** A	CAATACATTAAGTGTGGAGGTAACTATTAGTGGTTGATTTTACTATTTCTTTGA	5' primer of *nip* to change the *nip* start codon to stop codon
pJL *nip** B	TCAAAGAAATAGTAAAATCAACCACTAATAGTTACCTCCACACTTAATGTATTG	3' primer of *nip* to change the *nip* start codon to stop codon
delsalA2 A	GTATCGATAAGCTTGATATCGAATTCCTGCAGCCCGGGGGATCTCAATCACCGAGGAATATAGTGCTAG	5' primer for 5' region of *salA2* to delete *salA2*
	GTCCACTTATCAAGATTGTTTTGAT	3' primer for 5' region of *salA2* to delete

delsalA2 B	CTCCCTTCTGTTAGTATGTAG	*salA2*
delsalA2 C	CTACATACTAACAGAAGGGAGATC AAAACAATCTTGATAAGTGGAC	5' primer for 3' region of *salA2* to delete *salA2*
delsalA2 D	CTCACGTTAAGGGATTTTGGTCAT GAGATTATCAAAAAGGATCTTTCCA TATGTAAGTCCGTAATGG	3' primer for 3' region of *salA2* to delete *salA2*
*delsalA2*_seq_F	CTCGGCAAGAAACAAGAATAAGAG	5' primer for 5' region of *salA2* to sequence the region
*delsalA2*_seq_R	CTAAAACTAGCGTTTTCATTGAAAG GAAC	3' primer for 3' region of *salA2* to sequence the region
delsalB A	GTATCGATAAGCTTGATATCGAATT CCTGCAGCCCGGGGGATC GAGGTTTTGAAGGATTTTCTTCTAT	5' primer for 5' region of *salB* to delete *salB*
delsalB B	AATTTAGGCTTATAGAACGTAATTG GAAATACCTCACTGATTAA	3' primer for 5' region of *salB* to delete *salB*
delsalB C	TTAATCAGTGAGGTATTTCCAATTA CGTTCTATAAGCCTAAATT	5' primer for 3' region of *salB* to delete *salB*
delsalB D	CTCACGTTAAGGGATTTTGGTCAT GAGATTATCAAAAAGGATCCAATAA TAATCACCACAATCAATA	3' primer for 3' region of *salB* to delete *salB*
	GGCTGATGTTAGGTTGGAAAATCC	5' primer for 5' region

delsalB _seq_F	G	of *salB* to sequence the region
delsalB _seq_R	CTATCTCACAGTTTTGCATATCA	3’ primer for 3’ region of *salB* to sequence the region
Δ*sar* A	CATGATTACGCCAAGCTTGGTACCGAGCTCGGATCCTTCTGTACAAGAGCTTGTATTGG	5’ primer for 5’ region of *sarC* to delete *PK/NRPs* BGC
Δ*sar* B	GTACTTAATTCAACTTCCATTCAATACTACTACCATTATATATT	3’ primer for 5’ region of *sarC* to delete *PK/NRPs* BGC
Δ*sar* C	AATATATAATGGTAGTAGTATTGAATGGAAGTTGAATTAAGTAC	5’ primer of Chloramphenicol to delete *PK/NRPs* BGC
Δ*sar* D	GTTGACAGTTGAGTAATAAAAGACATTAGAAAACCGACTGTAAAAAG	3’ primer of Chloramphenicol to delete *PK/NRPs* BGC
Δ*sar* E	CTTTTTACAGTCGGTTTTCTAATGTCTTTTATTACTCAACTGTCAAC	5’ primer for 3’ region of *sarP* to delete *PK/NRPs* BGC
Δ*sar* F	CGAATTCCACACACTGGCGGCCGTTACTAGTGGATCGGTCTAGGCAACTCCCAGGTTCTTC	3’ primer for 3’ region of *sarP* to delete *PK/NRPs* BGC
speB C192S F	CAACATGCAGCTACAGGAAGTGTTGCTACTGCAACTGC	5’ primer to introduce C192S mutation in

		speB CDS
speB C192S R	GCAGTTGCAGTAGCAACACTTCCT GTAGCTGCATGTTG	3’ primer to introduce C192S mutation in *speB* CDS
tufA top	CAATCGGACACGTTGACCACGG	5’ primer to check the megaplasmid copy number
tufA bottom	CTGAAAGAAGGTCACGGATTTCC	3’ primer to check the megaplasmid copy number
tufA qRTFwd	CAACTCGTCACTATGCGCACAT	5’ primer for GAS *tufA* qRT-PCR
tufA qRTRev	GAGCGGCACCAGTGATCAT	3’ primer for GAS *tufA* qRT-PCR
tufA qRTFwd	AAAAACGTCACTACGCTCACAT	5’ primer for SAL *tufA* qRT-PCR
tufA qRTRev	GAGCGGCACCAGTGATCAT	3’ primer for SAL *tufA* qRT-PCR
*sarD*_qRT Fwd	GATGGCAGTTCAAATGGGATTAC	5’ primer for *nrps* qRT-PCR

*sarD*_qRT Rev	CTGTACCATGACACTCCACAG	3’ primer for *nrps* qRT-PCR
*speB*_qRT Fwd	ACTCTACCAGCGGATCATTTG	5’ primer for *speB* qRT-PCR
*speB*_qRT Rev	CAGCGGTACCAGCATAAGTAG	3’ primer for *speB* qRT-PCR

1 Bloudoff, K., Fage, C. D., Marahiel, M. A. & Schmeing, T. M. Structural and mutational analysis of the nonribosomal peptide synthetase heterocyclization domain provides insight into catalysis. *Proc. Natl. Acad. Sci. USA* **114**, 95-100 (2017).

2 Zhang, F. *et al.* MS-Derived Isotopic Fine Structure Reveals Forazoline A as a Thioketone-Containing Marine-Derived Natural Product. *Org. Lett.* **22**, 1275-1279 (2020).

3 Beres, S. B. *et al.* Molecular complexity of successive bacterial epidemics deconvoluted by comparative pathogenomics. *Proc. Natl. Acad. Sci. USA* **107**, 4371-4376 (2010).

4 Sumby, P. *et al.* Evolutionary origin and emergence of a highly successful clone of serotype M1 group A Streptococcus involved multiple horizontal gene transfer events. *J. Infect. Dis.* **192**, 771-782 (2005).

5 Fittipaldi, N. *et al.* Full-genome dissection of an epidemic of severe invasive disease caused by a hypervirulent, recently emerged clone of group A Streptococcus. *Am. J. Pathol.* **180**, 1522-1534 (2012).

6 Beres, S. B. *et al.* Transcriptome remodeling contributes to epidemic disease caused by the human pathogen *Streptococcus pyogenes*. *MBio* **7**, e00403-00416 (2016).

7 Beres, S. B. *et al.* Molecular genetic anatomy of inter-and intraserotype variation in the human bacterial pathogen group A Streptococcus. *Proc. Natl. Acad. Sci. USA* **103**, 7059-7064 (2006).

8 Makthal, N. *et al.* Structural and functional analysis of RopB: a major virulence regulator in *Streptococcus pyogenes*. *Mol. Microbiol.* **99**, 1119-1133 (2016).

9 Do, H. *et al.* Leaderless secreted peptide signaling molecule alters global gene expression and increases virulence of a human bacterial pathogen. *Proc. Natl. Acad. Sci. USA* **114**, E8498-E8507, doi:10.1073/pnas.1705972114 (2017).

10 Barretto, C., Alvarez-Martin, P., Foata, F., Renault, P. & Berger, B. Genome sequence of the lantibiotic bacteriocin producer *Streptococcus salivarius* strain K12. *J. Bacteriol.* **194**, 5959-5960 (2012).

11 Li, J., Kasper, D. L., Ausubel, F. M., Rosner, B. & Michel, J. L. Inactivation of the α C protein antigen gene, *bca*, by a novel shuttle/suicide vector results in attenuation of virulence and immunity in group B Streptococcus. *Proc. Natl. Acad. Sci. USA* **94**, 13251-13256 (1997).
