# Supplementary material for: Engineered probiotic overcomes pathogen defences using signal interference and antibiotic production to treat infection in mice
Source: Nat Microbiol. 2024 Jan 16;9(2):502–13. doi: 10.1038/s41564-023-01583-9 (PMC10847043; doi:10.1038/s41564-023-01583-9)
Supplement: Supplementary file 2 — Reporting Summary [file 41564_2023_1583_MOESM2_ESM.pdf]

nature portfolio

Corresponding author(s): Muthiah Kumaraswami

Last updated by author(s): Sep 3, 2023

Reporting Summary

Nature Portfolio wishes to improve the reproducibility of the work that we publish. This form provides structure for consistency and transparency in reporting. For further information on Nature Portfolio policies, see our Editorial Policies and the Editorial Policy Checklist.

Statistics

For all statistical analyses, confirm that the following items are present in the figure legend, table legend, main text, or Methods section.

n/a	Confirmed	
☐	☒	The exact sample size (*n*) for each experimental group/condition, given as a discrete number and unit of measurement
☐	☒	A statement on whether measurements were taken from distinct samples or whether the same sample was measured repeatedly
☐	☒	The statistical test(s) used AND whether they are one- or two-sided<br>*Only common tests should be described solely by name; describe more complex techniques in the Methods section.*
☒	☐	A description of all covariates tested
☒	☐	A description of any assumptions or corrections, such as tests of normality and adjustment for multiple comparisons
☐	☒	A full description of the statistical parameters including central tendency (e.g. means) or other basic estimates (e.g. regression coefficient) AND variation (e.g. standard deviation) or associated estimates of uncertainty (e.g. confidence intervals)
☐	☒	For null hypothesis testing, the test statistic (e.g. *F*, *t*, *r*) with confidence intervals, effect sizes, degrees of freedom and *P* value noted<br>*Give P values as exact values whenever suitable.*
☒	☐	For Bayesian analysis, information on the choice of priors and Markov chain Monte Carlo settings
☒	☐	For hierarchical and complex designs, identification of the appropriate level for tests and full reporting of outcomes
☒	☐	Estimates of effect sizes (e.g. Cohen's *d*, Pearson's *r*), indicating how they were calculated

Our web collection on statistics for biologists contains articles on many of the points above.

Software and code

Policy information about availability of computer code

Data collection	No custom software was used for data collection.
Data analysis	No custom software or algorithms were used for data analysis. Graphpad prism was used for data analysis and statistical significance among groups.

For manuscripts utilizing custom algorithms or software that are central to the research but not yet described in published literature, software must be made available to editors and reviewers. We strongly encourage code deposition in a community repository (e.g. GitHub). See the Nature Portfolio guidelines for submitting code & software for further information.

Data

Policy information about availability of data

All manuscripts must include a data availability statement. This statement should provide the following information, where applicable:

- Accession codes, unique identifiers, or web links for publicly available datasets
- A description of any restrictions on data availability
- For clinical datasets or third party data, please ensure that the statement adheres to our policy

Data supporting the findings of this study are available in this article and its Supplementary Information files, or from the corresponding author upon request. Genomes of S. salivarius K12 and MGAS10870 were sequenced previously, and sequences are publicly available. Primers used in this study are provided in Supplementary Table 2. Minimum datasets that are necessary to interpret, verify, and extend the research in the article are provided as source data with this paper.

Field-specific reporting

Please select the one below that is the best fit for your research. If you are not sure, read the appropriate sections before making your selection.

☒ Life sciences ☐ Behavioural & social sciences ☐ Ecological, evolutionary & environmental sciences

For a reference copy of the document with all sections, see nature.com/documents/nr-reporting-summary-flat.pdf

Life sciences study design

All studies must disclose on these points even when the disclosure is negative.

Sample size	For in vitro and ex vivo experiments, 3 biological replicates were analyzed in duplicates.<br>For in vivo experiments, 10 or more animals were used for each group. No statistical method was used to predetermine sample size.
Data exclusions	No data exclusions.
Replication	Experiments were performed using 3 biological replicates that were analyzed in duplicates and data from biological replicates were consistent with each other.
Randomization	Animals were assigned randomly to different experimental groups. Random allocation was not relevant for in vitro experiments as vast majority of the experiments compare the effects of different mutant strains.
Blinding	Animal infection experiments were performed in a blinded fashion. The investigators were blinded for the group assignment in animal infection studies. All biological samples were processed and analyzed blindly regarding the experimental groups.

Reporting for specific materials, systems and methods

We require information from authors about some types of materials, experimental systems and methods used in many studies. Here, indicate whether each material, system or method listed is relevant to your study. If you are not sure if a list item applies to your research, read the appropriate section before selecting a response.

Materials & experimental systems

n/a	Involved in the study	
☐	☒	Antibodies
☒	☐	Eukaryotic cell lines
☒	☐	Palaeontology and archaeology
☐	☒	Animals and other organisms
☐	☒	Human research participants
☒	☐	Clinical data
☒	☐	Dual use research of concern

Methods

n/a	Involved in the study	
☒	☐	ChIP-seq
☒	☐	Flow cytometry
☒	☐	MRI-based neuroimaging

Antibodies

Antibodies used	Antibodies raised against recombinant SpeB protease was used to detect secreted SpeB levels. Anti SpeB primary antibodies were used at 1:100,000 dilution, whereas the secondary antibodies were used at 1: 50,000 dilution. Antirabbit HRP-conjugated secondary antibodies were purchased from Thermo Scientific (Catalog no. SA1-9510).
Validation	The antibody was used in several previously published studies and validated for its specific binding to SpeB. SpeB-specific reactivity and lack of cross reactivity of anti SpeB antibodies with other GAS proteins were verified in previous published studies.

Animals and other organisms

Policy information about studies involving animals; ARRIVE guidelines recommended for reporting animal research

Laboratory animals	CD-1 mice, 3-10 weeks old, female mice/ C57BL/6 mice, 4-6 weeks old, female mice
Wild animals	None
Field-collected samples	None
Ethics oversight	Houston Methodist Research Institute IACUC, and Emory School of Medicine IACUC<br>Full information of the committee, study protocol, and approved protocol numbers are provided in the manuscript.

Note that full information on the approval of the study protocol must also be provided in the manuscript.

Human research participants

Policy information about studies involving human research participants

Population characteristics	*Describe the covariate-relevant population characteristics of the human research participants (e.g. age, gender, genotypic information, past and current diagnosis and treatment categories). If you filled out the behavioural & social sciences study design questions and have nothing to add here, write "See above."*
Recruitment	*Describe how participants were recruited. Outline any potential self-selection bias or other biases that may be present and how these are likely to impact results.*
Ethics oversight	*Identify the organization(s) that approved the study protocol.*

Note that full information on the approval of the study protocol must also be provided in the manuscript.
